# In the Multi-domain Protein Adenylate Kinase, Domain Insertion Facilitates Cooperative Folding while Accommodating Function at Domain Interfaces

**DOI:** 10.1371/journal.pcbi.1003938

**Published:** 2014-11-13

**Authors:** V. V. Hemanth Giri Rao, Shachi Gosavi

**Affiliations:** National Centre for Biological Sciences, Tata Institute of Fundamental Research, Bangalore, India; Icahn School of Medicine at Mount Sinai, United States of America

## Abstract

Having multiple domains in proteins can lead to partial folding and increased aggregation. Folding cooperativity, the all or nothing folding of a protein, can reduce this aggregation propensity. In agreement with bulk experiments, a coarse-grained structure-based model of the three-domain protein, *E. coli* Adenylate kinase (AKE), folds cooperatively. Domain interfaces have previously been implicated in the cooperative folding of multi-domain proteins. To understand their role in AKE folding, we computationally create mutants with deleted inter-domain interfaces and simulate their folding. We find that inter-domain interfaces play a minor role in the folding cooperativity of AKE. On further analysis, we find that unlike other multi-domain proteins whose folding has been studied, the domains of AKE are not singly-linked. Two of its domains have two linkers to the third one, i.e., they are inserted into the third one. We use circular permutation to modify AKE chain-connectivity and convert inserted-domains into singly-linked domains. We find that domain insertion in AKE achieves the following: (1) It facilitates folding cooperativity even when domains have different stabilities. Insertion constrains the N- and C-termini of inserted domains and stabilizes their folded states. Therefore, domains that perform conformational transitions can be smaller with fewer stabilizing interactions. (2) Inter-domain interactions are not needed to promote folding cooperativity and can be tuned for function. In AKE, these interactions help promote conformational dynamics limited catalysis. Finally, using structural bioinformatics, we suggest that domain insertion may also facilitate the cooperative folding of other multi-domain proteins.

## Author Summary

Most individual protein domains fold in an all or nothing fashion. This cooperative folding is important because it reduces the existence of partially folded proteins which can stick to each other and create disease causing aggregates. However, numerous proteins have multiple domains, independent units of folding, stability and/or function. Several such proteins also fold cooperatively. It is thought that strong interactions between individual domains allow the folding to propagate from a nucleating domain to neighbouring ones and this enables cooperative folding in multi-domain proteins. Here, we computationally study the folding of the three-domain protein AKE and find instead that the topology of the protein, wherein the two less stable domains are inserted into the more stable one, promotes folding cooperativity. When the more stable domain is folded, the ends of the inserted domains are constrained and this allows them to fold easily. In such a protein topology, strong inter-domain interactions are not needed to promote folding cooperativity. Interface amino acids which would have been involved in ensuring that the domains fit together correctly can now be tuned for binding or catalysis or conformational transitions. Thus, inserted domains may be present in multi-domain proteins to promote both function and folding.

## Introduction

**Figure 1:**
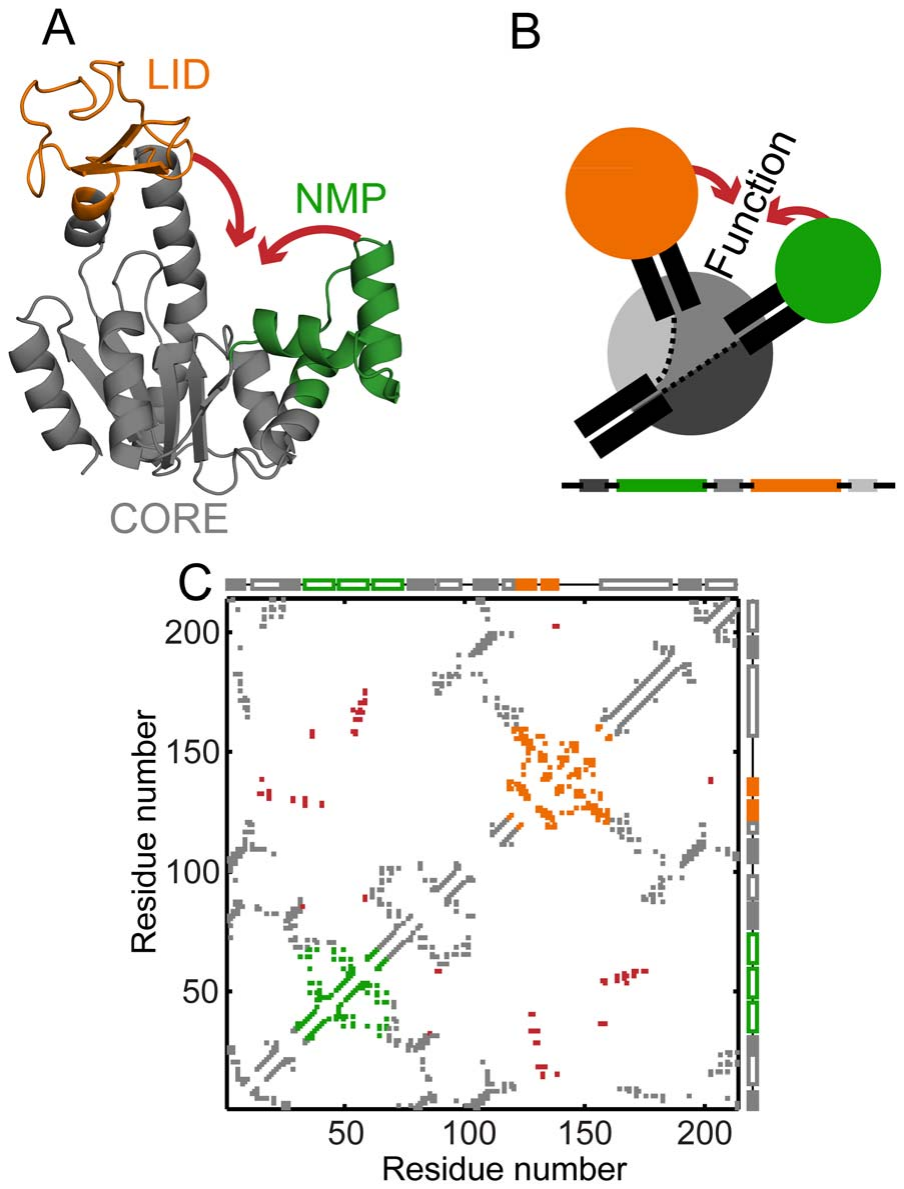
Structure, cartoon and contact map of WT AKE. (A) WT AKE (open state, PDB: 4AKE, chain A) colored by domain: CORE (grey; residues 1–29, 68–117 (CORE-N) and 161–214 (CORE-C)), NMP (green; residues 30–67) and LID (orange; residues 118–160). The conformational transitions of LID and NMP are indicated by red arrows. All structures in this article were drawn using the PyMOL Molecular Graphics System (Version 1.4.1 Schrödinger, LLC). (B) Cartoon showing the domain organization of AKE on the folded structure (top) and the sequence (bottom) colored as in A. The CORE domain is split into three grey regions because of domain insertion. (C) The C-α contact map of WT AKE. X and Y axes represent residue number. Secondary structure is shown along the axes: α helices are empty boxes and β strands are filled boxes. The contacts are colored according to their location (CORE: grey, NMP: green, LID: orange). The absolute contact order of these regions is: LID: 15.27, NMP: 10.42, CORE: 62.87, and the entire WT AKE: 46.04. The red contacts are closed state specific contacts that drive the conformational transitions.

The presence of multiple domains in proteins can lead to interactions between partially folded domains and in turn to increased misfolding and aggregation [1]. Nevertheless, several multi-domain proteins fold reversibly in vitro [2], [3]. Cooperative folding, the all or nothing folding of a protein with the population of few intermediates, reduces partially folded states [4]. It has been hypothesized that folding cooperativity has evolved in proteins to avoid misfolding and decrease aggregation propensity [5]. Strong inter-domain interactions have been implicated in the cooperative folding of multi-domain proteins [1], [6]–[10]. Here, we computationally investigate the role of inter-domain interactions in the folding of the three-domain protein *E. coli* Adenylate kinase (AKE) (Fig. 1) and find that an altogether different method, domain insertion, promotes folding cooperativity. Domain insertion is the presence of the amino acid sequence of one domain (the inserted domain) within the sequence of another domain (the discontinuous domain) (Fig. 1B). In the three-dimensional structure, the discontinuous (along the protein chain) amino acid stretches of the discontinuous domain (Fig. 1B, bottom, grey segments) fold together into a single domain (Fig. 1B, top, grey regions).

Domain insertion makes protein topology more complex and this will likely slow folding [11]. Why then is domain insertion preferred over tuning the strength of inter-domain interactions as a method to promote folding cooperativity? It has been shown that slow and complex folding arises in order to accommodate function [12]. We hypothesize that in AKE too, domain insertion and the potential slow folding are tolerated for functional reasons. Domain interfaces in many multi-domain proteins are involved in function, i.e., they take part in binding or catalysis or act as hinges to facilitate conformational transitions [13] and it may not be possible to tune such functional interfaces to promote folding [12]. Here, we confirm our hypothesis by computationally studying the mechanism of conformational transitions of AKE.

The 214 residue enzyme, AKE, has become an important model for the study of both protein folding and conformational transitions [14]–[32]. AKE has three domains termed CORE, LID and NMP (Fig. 1A). Both the N- and C-termini of AKE are present in CORE, which is split into three discontinuous polypeptide segments by the two inserted domains, LID and NMP (Fig. 1B). In experiment, AKE folds cooperatively with two-state thermodynamics [23]. AKE reversibly catalyzes the reaction ATP+AMP = 2ADP [14]. The substrates ATP and AMP preferentially bind at the CORE-LID and CORE-NMP interfaces respectively [15]. LID and NMP then close over CORE and this extensively studied conformational transition [16]–[23] achieves the right geometry for catalysis [14], [15]. The closure of LID before NMP is hypothesized to prevent misligation of the substrates [19].

**Figure 2:**
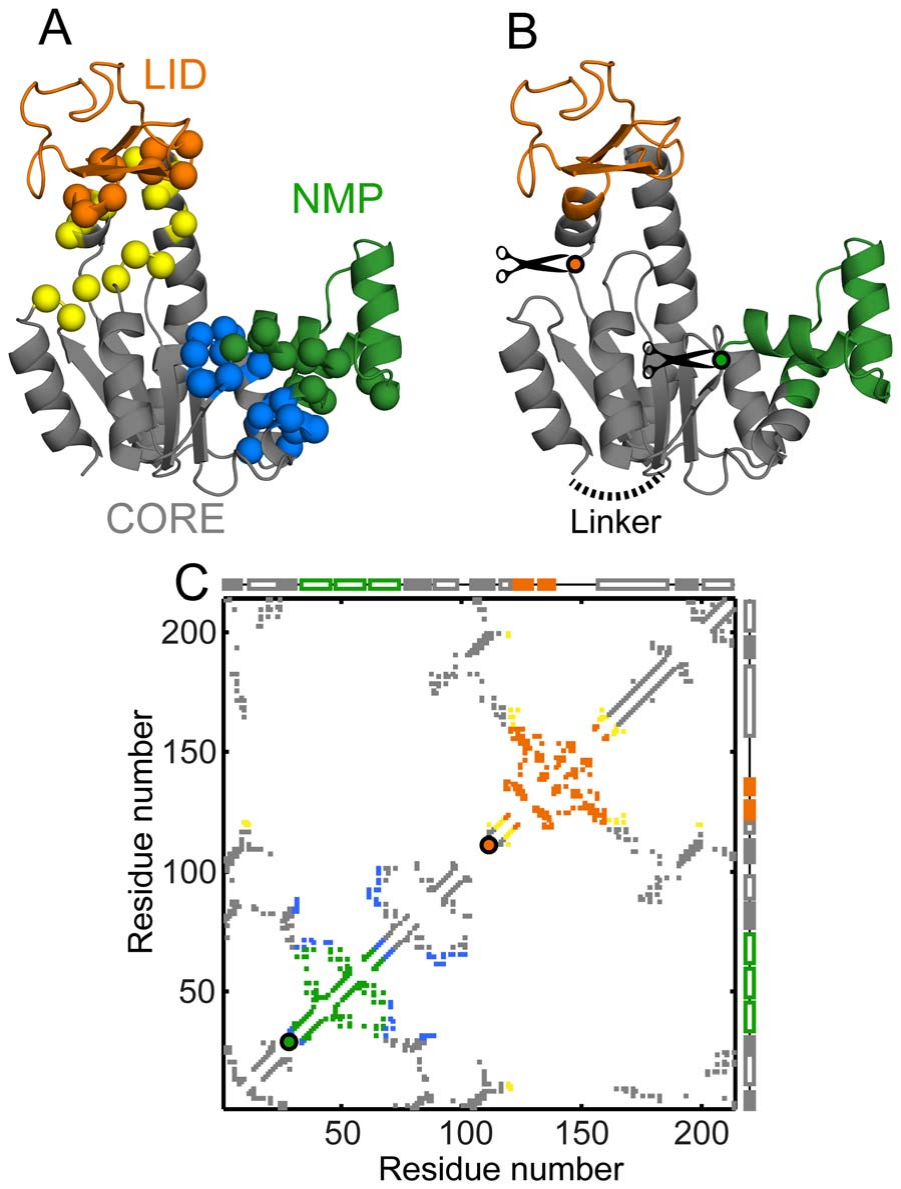
Structures and contact maps of mutants of AKE. Domains of AKE are colored as in Fig. 1 (CORE: grey, NMP: green, LID: orange). (A) Interface deletion mutants of AKE. All marked residues are represented by their C-α atoms. The CORE-NMP interface is composed of interactions between the green (NMP) and the blue (CORE) residues. The mutant, ΔCORE-NMPi, has these interactions deleted. The CORE-LID interface is composed of interactions between the orange (LID) and the yellow (CORE) residues. This interface is deleted in the mutant ΔCORE-LIDi. (B) Circular permutants of AKE. The WT N- and C-termini are linked by a 4 glycine loop (dotted black line). New N- and C-termini are generated by cutting at one of the positions indicated by the circles (CP-NMPcut: green; between residues 29 and 30; CP-LIDcut: orange; between residues 111 and 112). (C) The C-α contact map of the open state of WT AKE with the intra-domain contacts colored as in Fig. 1C. The CORE-NMP interface interactions (33 blue contacts) are deleted in ΔCORE-NMPi while the CORE-LID interface interactions (22 yellow contacts) are deleted in ΔCORE-LIDi. The absolute contact order of the interfaces is: CORE-NMP interface: 29.58 and CORE-LID interface: 39.73. Colored circles (CP-NMPcut: green and CP-LIDcut: orange) mark the (x, x) location of the first residue x, of the CPs. The closed state specific contacts (Fig. 1C, red contacts; appropriately renumbered in the CPs) are not shown here but are present in the conformational transition simulations.

Structure-based models (SBMs) capture the funnelled energy landscape of proteins [33] by encoding the native structure into their potential energy functions [34]. MD simulations of SBMs have successfully reproduced the folding routes and the folding rates of diverse proteins [1], [8], [9], [12], [32], [34]. We find in agreement with experiment that a C-α structure-based model (C-α SBM) of AKE folds cooperatively. In order to test the role of inter-domain interactions in this cooperative folding, we create AKE mutants where these interactions are deleted (Fig. 2A, 2C). MD simulations of these mutants show that the inter-domain interactions play a minimal role in promoting folding cooperativity. We then create circular permutants (CPs) of AKE where either LID or NMP (the inserted domains) are converted to singly-linked domains (Fig. 2B, 2C) and find that the CPs fold less cooperatively than WT AKE. Thus, domain insertion rather than inter-domain interactions promotes folding cooperativity in AKE.

A dual-SBM which includes information from both the ligand free and the ligand bound structures of AKE has been developed to understand the conformational transitions of WT AKE [18], [19]. We use this mechanism of conformational transitions as an assay for function and test if the mechanism is WT-like in the AKE mutants. We find that the mutants with the deleted inter-domain interactions (which show WT-like folding mechanism and cooperativity) show altered mechanisms of conformational transitions. The CPs, on the other hand, have a WT-like mechanism. Together, our results show that domain insertion promotes folding cooperativity in *E. coli* Adenylate kinase while inter-domain interactions are optimized for conformational transitions.

## Results

### Thermodynamic folding cooperativity

We perform all simulations close to the folding temperature, T_f_, where multiple transitions occur between the equally populated folded and unfolded ensembles and the best sampling of the transition region is achieved. The presence of a single free energy barrier separating the native and unfolded ensembles at T_f_ implies that the protein folds cooperatively [4]. If the different domains of a multi-domain protein fold at different T_f_s, partially folded states get populated at temperatures between the lowest and the highest domain specific T_f_s. Upon mutation, a domain specific decrease of T_f_ can result in the incomplete folding of that domain at the T_f_ of the whole protein and the population of partially folded states in the folded ensemble. This results in reduced folding cooperativity.

Folding cooperativity is usually deduced from the heat capacity curve (C_v_(T) vs. T) using the ratio of the van't Hoff enthalpy (ΔH_vH_) to the calorimetric enthalpy (ΔH_cal_) [4]. If this curve has a single narrow peak at T_f_ then the folding transition is cooperative. We make perturbations to WT AKE which change the free-energetic balance between domains and in turn the folding cooperativity. However, since the domains of AKE are of unequal size (CORE≫LID>NMP) the largest contribution to C_v_(T_f_) comes from the folding of CORE which is not perturbed much across simulations. Thus, the ΔH_vH_/ΔH_cal_ is not a sensitive measure of the folding cooperativity of AKE and its mutants (however, it does show the same trend as seen in the following sections; Fig. S3). Therefore, we use the height of the free energy barrier at T_f_ and the “foldedness” of the protein in this native ensemble to infer the degree of cooperative folding. We define foldedness as the ratio of the population of a mutant at the value of the reaction coordinate where WT AKE is folded to the population of WT AKE at the value of the reaction coordinate where WT AKE is folded. This definition inherently assumes that the value of the reaction coordinate where WT AKE is folded is greater than or equal to the value of the reaction coordinate where the mutants are folded.

### A C-α SBM reproduces the main features of folding experiments on WT AKE

**Figure 3:**
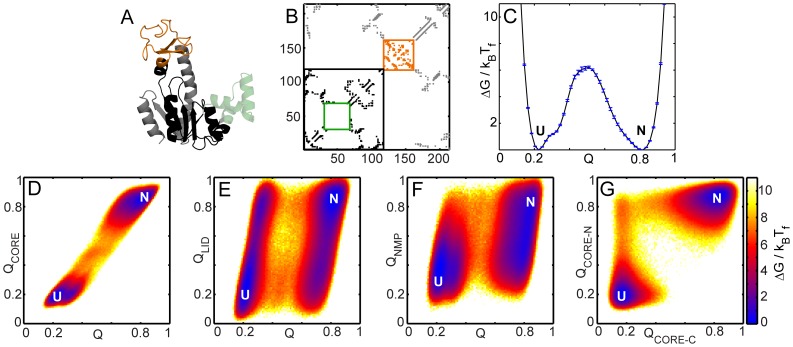
Contact clusters and free energy profiles of WT AKE. (A) The three contact clusters whose contacts form and break together during the folding and unfolding of WT AKE are projected onto their residues and shown on the structure: CORE-N (black; residues 1–29 and 68–117), LID (orange) and CORE-C (grey; residues 161–214). No contact cluster was found in NMP (green). (B) The contacts of the residues that form the clusters are shown in the same colors as in A. The intra-LID and intra-NMP contacts are enclosed in orange and green boxes and their contact location close to the diagonal underlines that LID and NMP are inserted domains. The CORE-N contacts are demarcated by the black box. (C–G) Reaction coordinates (RCs) for the folding free energy plots are defined as the fraction of native contacts formed in the whole protein (Q; all contacts, Fig. 2C), CORE-N (Q_CORE-N_; black contacts in B), CORE-C (Q_CORE-C_; grey contacts in B), CORE (Q_CORE_; grey contacts, Fig. 2C), LID (Q_LID_; orange contacts, Fig. 2C) and NMP (Q_NMP_; green, Fig. 2C). The more folded a given region, the higher the value of the corresponding Q. (C) The scaled folding free energy (ΔG/k_B_T_f_) of WT AKE plotted as a function of Q. N and U denote the native and the unfolded ensembles. The error bars represent twice the square root of the variance in the folding free energy and were calculated using a jackknife algorithm. (D) 2DFES plotted with RCs of Q_CORE_ and Q. The free energies are scaled by k_B_T_f_ for all 2DFESs. The color indicates the height of the free energy at a given value of (x, y) and the limits of the color scale are the same as the limits of the y-axis in C. (E) The 2DFES plot with RCs of Q_LID_ and Q. (F) The 2DFES plot with RCs of Q_NMP_ and Q. (G) The 2DFES plot with RCs of Q_CORE-N_ and Q_CORE-C_ shows that CORE-N folds before CORE-C in the predominant folding route.

Both the conformational transitions and the folding of AKE have previously been studied using different flavors of SBMs [18], [19], [32]. Here, we use a well-tested SBM [34] which uses only the C-α atom to represent the entire residue. This C-α SBM, which uses the folded structure (Fig. 1A, 2A, 2B) and its contact map (Fig. 1C, 2C) as inputs, is sufficient to capture the main changes in folding upon topological perturbation [35], [36] and is not intended for the detailed analysis of structural populations [2], [32]. We first validate the C-α SBM by performing folding simulations of WT AKE (Fig. 3) and show that these broadly agree with results from diverse ensemble experiments (HX-NMR [31], tryptophan fluorescence [24], and time-resolved FRET [26]–[30]).

We calculate the correlation coefficients of the formation of all pairs of native contacts using MD simulation trajectories of only the transitions between the folded and the unfolded states (or between the unfolded and the folded states) of WT AKE. Using these correlation coefficients, we partition native contacts into three clusters (Fig. 3A, 3B) such that the contacts in each cluster form and break together (See SI Methods, section 4 and Fig. S1). These contact clusters correspond well with the three foldons determined earlier in HX-NMR experiments [31]. The three clusters correspond to LID (contacts between residues 118–160), the N-terminal part of CORE (CORE-N: contacts between residues 1–29, 68–117 and between these residues and NMP residues 30–67) and the C-terminal part of CORE (CORE-C: contacts between residues 161–214 and contacts between these and the CORE-N residues). Native contacts from NMP (Fig. 2C, green) and the CORE-LID interface (Fig. 2C, yellow) are not part of any cluster (Fig. 3B. S1).

We next analyze folding routes by plotting the free energy profile (FEP) and the 2-dimensional free energy surfaces (2DFESs) for WT AKE along several suitable reaction coordinates (Fig. 3C–G). The FEP (Fig. 3C) shows a large free energy barrier (∼6 k_B_T_f_) separating the unfolded (Q∼0.2) and the native (Q∼0.8) ensembles. There is a slight dip at Q∼0.3 due to the population of states with only LID folded (Fig. 3E). The single free energy barrier means that bulk probes such as circular dichroism (CD) and FRET observe a cooperative 2-state folding transition [2], [23]. In agreement with experiments [17], this transition corresponds to the folding/unfolding of CORE (Fig. 3D). Fig. 3E and 3F show that LID can be either folded or unfolded in the N and U ensembles while NMP cannot fold completely in U. In agreement with experiments [23], [31], both LID and NMP can either be folded or unfolded once CORE is folded (N in Fig. 3E–F).

**Figure 4:**
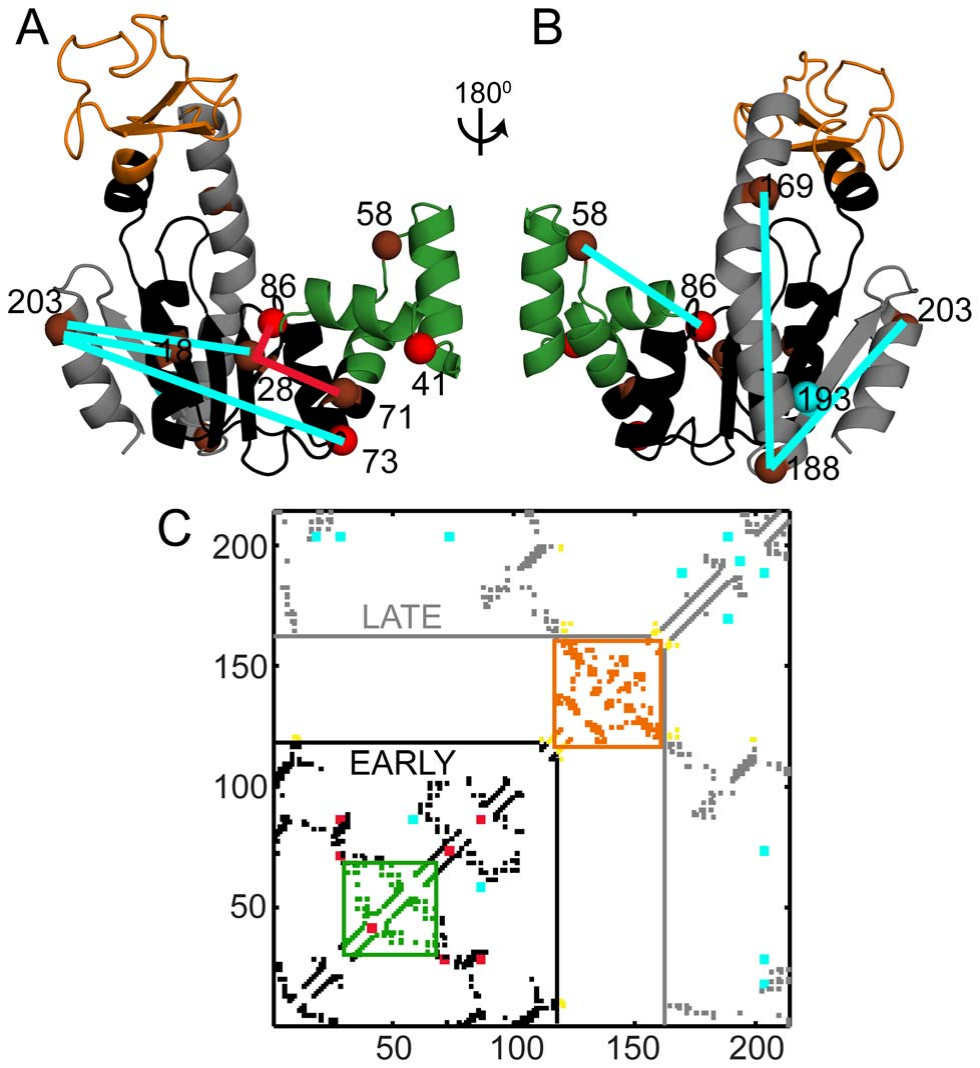
Comparison of simulations with experimental refolding kinetics data. (A–B) WT AKE colored according to the contact clusters from Fig. 3A. NMP, which is not part of the clusters is shown in green as in Fig. 1A. Red (fast folding in experiment) and cyan (slow folding in experiment) spheres mark the positions of the C-α atoms of single tryptophan mutations used to study refolding kinetics using intrinsic fluorescence [24]. Brown spheres mark the positions of C-α atoms of FRET pairs used to study refolding kinetics by time resolved FRET [26]–[30]. Residues 73 and 86 were used in both experiments. Red lines connect the experimental early forming FRET distances while cyan lines connect the experimental late forming FRET distances. The probe residues lie in either CORE-N (black), CORE-C (grey) or NMP (green). (C) The native contacts of AKE colored similar to 3B, 4A and 4B: CORE-N (black), CORE-C (grey), LID (orange), NMP (green), and CORE-LID interface (yellow). The regions which fold early (CORE-N, black) and late (CORE-C, grey) during simulations (Fig. 3G) are demarcated by black and grey lines respectively. The red (early forming) and cyan (late forming) squares along the diagonal correspond to the single tryptophan mutants from A, B. Off diagonal red (early forming) and cyan (late forming) squares mark the FRET pairs joined by red and cyan lines in A, B.

Intrinsic fluorescence from single tryptophan (Trp) mutants has been used to study the refolding of AKE (24). These experiments show that regions of AKE near residues 41 (part of NMP), 86 and 73 (both part of CORE-N) (red spheres in Fig. 4A, B) fold faster than the region near residue 193 (part of CORE-C; cyan sphere in Fig. 4B). Thus, except for residue 41, the main folding route from our simulations where CORE-N folds before CORE-C (Fig. 3G) rationalizes the behaviour of the single Trp mutant fluorescence experiments. Further, we observe another route (∼10% of transitions) where CORE-C folds before CORE-N, in qualitative agreement with single molecule FRET data [2].

Refolding kinetics of WT AKE were also monitored by time resolved FRET [26]–[30]. In these experiments, the FRET donor acceptor pairs were located at: 58–86 (between CORE-N and NMP); 28–71 and 28–86 (both intra-CORE-N); 73–203, 28–203, 18–203 (all between CORE-N and CORE-C); 188–203, 169–188 (both intra-CORE-C) (Fig. 4A, B). The rates of formation of these distances in experiment broadly agree with the major folding route found in our simulations (CORE-N folds first followed by CORE-C) (Fig. 4C). In experiment, the burst phase distribution of the 58–86 (NMP-CORE-N) FRET distance resembled the unfolded state distribution and it adopted a native-like distribution only late during refolding [26]. NMP is inserted into CORE-N (Fig. 3B). CORE-N folds early in our simulations and the folding of NMP is dependent on the folding of CORE-N. Although, the complete folding of NMP occurs only after crossing the free energy barrier, specific NMP residues do fold early (Fig. 3F). Local energetic heterogeneities will modulate the order of formation of local contacts in NMP. If our coarse-grained simulations capture such local ordering correctly, it is only by chance. So, we choose to not interpret the specific temporal ordering of our NMP contacts and assume that NMP forms late during folding.

Overall, simulations of the AKE C-α SBM are able to integrate data from diverse bulk experiments into one folding mechanism and this model can be used for further protein perturbations.

### Removing inter-domain interactions has a minimal effect on folding cooperativity

**Figure 5:**
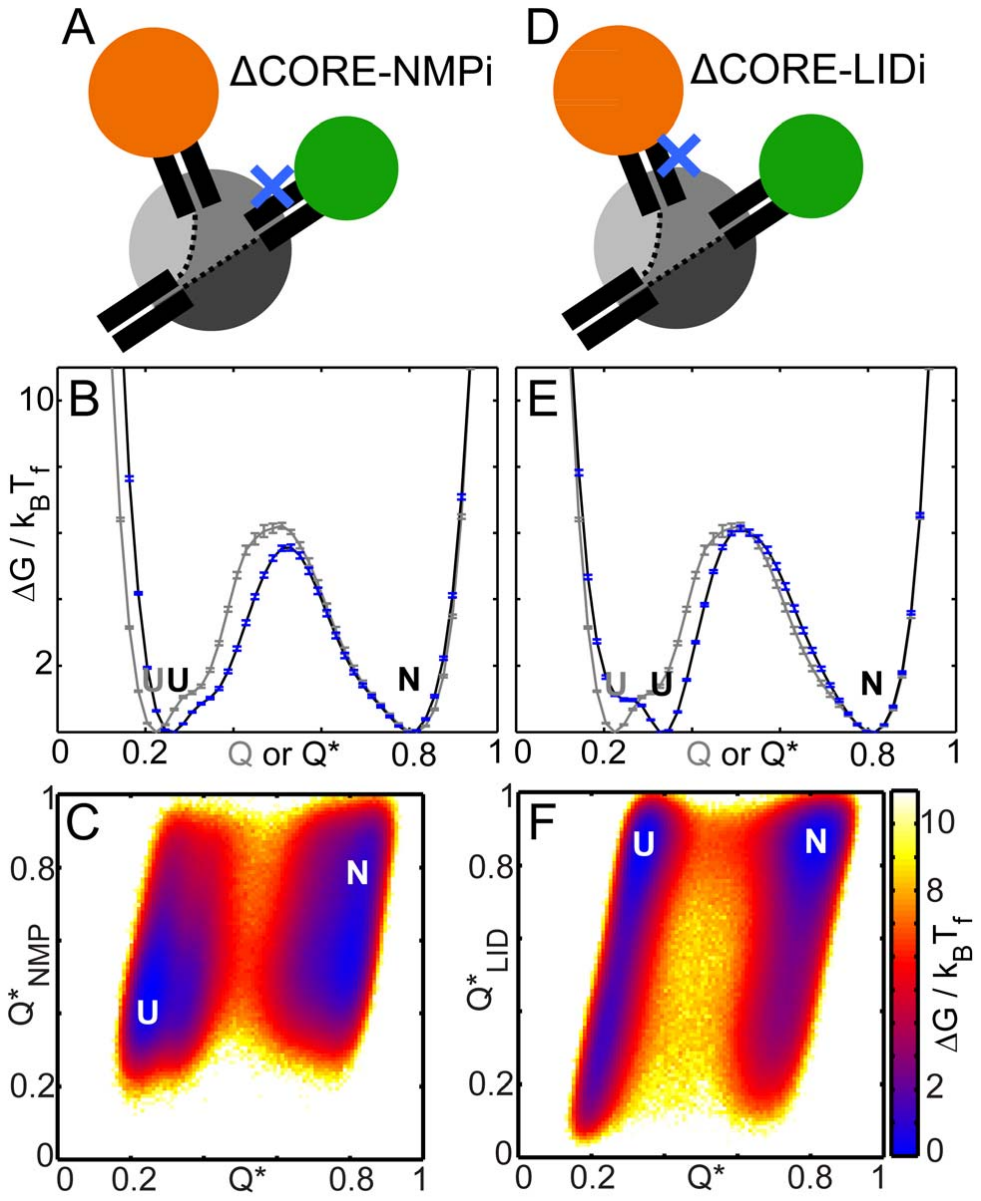
Folding of AKE mutants with deleted inter-domain interfaces. All free energies are scaled by their respective k_B_T_f_s. N and U denote the native and the unfolded ensembles. The error bars represent twice the square root of the variance in the folding free energy and were calculated using a jackknife algorithm. The blue Xs mark the position of the deleted interfaces. (A) Cartoon of the folded state of ΔCORE-NMPi at T_f_. (B) The FEP (black with blue error bars) shows a free energy barrier similar to that in WT (grey). (C) Q* and Q*_NMP_ are strength-scaled fraction of native contacts, e.g., a contact with a strength of 1.2 is counted as 1.2, when formed, in the calculation of Q* and Q*_NMP_. The 2DFES plot with RCs of Q*_NMP_ and Q* shows that NMP folds as in WT. (D) Cartoon of the folded state of ΔCORE-LIDi at T_f_. (E) The FEP (black with blue error bars) shows a free energy barrier similar to that in WT (grey). The U ensemble shifts to higher Q*. (F) The 2DFES plot with RCs of Q*_LID_ and Q* shows that the U ensemble has partially folded LID.

AKE has two inter-domain interfaces (Fig. 2A, 2C). We computationally create two mutants: one with no CORE-NMP inter-domain interactions (ΔCORE-NMPi; Fig. 5A) and another with no CORE-LID interactions (ΔCORE-LIDi; Fig. 5D). Similar mutants have been experimentally created to study AKE function [22]. However, when some contacts of an interface residue are deleted in the C-α SBM, it is enthalpically less stable when folded. In order to preserve WT-like energetic stabilization for every residue at the interface, we appropriately scale the strength of the other contacts of that residue when creating both ΔCORE-NMPi and ΔCORE-LIDi. This is similar to converting an outward facing residue which contributes to inter-domain interactions into an inward facing one which contributes to intra-domain interactions. We simulate C-α SBMs of these mutants to understand the effect of inter-domain interactions on the folding cooperativity of AKE.

The free energy barriers (∼5–6 k_B_T) of both ΔCORE-NMPi (Fig. 5B; black) and ΔCORE-LIDi (Fig. 5E; black) are similar to that of WT AKE (Fig. 5B, 5E, grey). Further, like in WT AKE, NMP and LID are folded in the native ensembles (Q or Q*∼0.8) of ΔCORE-NMPi and ΔCORE-LIDi respectively (“foldedness” of 1). The unfolded ensemble of ΔCORE-NMPi appears at a higher Q* (∼0.25) than that of WT (Q∼0.2, Fig. 5B). This is because some intra-NMP contacts are stronger in ΔCORE-NMPi than in WT and these stay folded. Additionally, they also contribute more to Q*. However, this is not sufficient to fold NMP completely in the unfolded state of ΔCORE-NMPi (Fig. 5C) and the folding mechanism of ΔCORE-NMPi is WT-like (Fig. S2). Overall, the CORE-NMP interface contributes little to the folding cooperativity of WT AKE. This interface is formed in the transition state of WT AKE (Fig. S5A, Q∼0.5) and its deletion should destabilize the transition state and affect the height of the free energy barrier. This does not happen because the energetic stabilization of interface residues is preserved in our model of ΔCORE-NMPi. The CORE-LID interface is significantly formed only in the native ensemble (Fig. S5B, Q∼0.5) and its deletion is unlikely to affect the transition state or the height of the free energy barrier.

The unfolded ensemble of ΔCORE-LIDi (Q*∼0.32) is higher than that of WT-AKE (Fig. 5E) and LID is partially folded in the unfolded ensemble of ΔCORE-LIDi (Fig. 5F). This could be interpreted as a loss in folding cooperativity. However, upon closer inspection we find that the same LID residues which contribute to inter-domain interactions contribute to interactions between the N- and C-termini of LID (Fig. 2A, orange spheres). When the intra-LID contacts of these residues are strengthened in order to create ΔCORE-LIDi, the N- and C-termini of LID stick to each other more. This constrains the LID domain in a manner similar to when it is inserted into a larger folded domain (even when CORE is unfolded). Reducing the strength of these contacts reduces the population of folded LID in the unfolded ensemble and makes the mechanism and the folding cooperativity more WT-like. We explore domain insertion further in the next section.

### CPs of WT AKE, with either LID or NMP converted to a singly-linked domain, have reduced folding cooperativity

**Figure 6:**
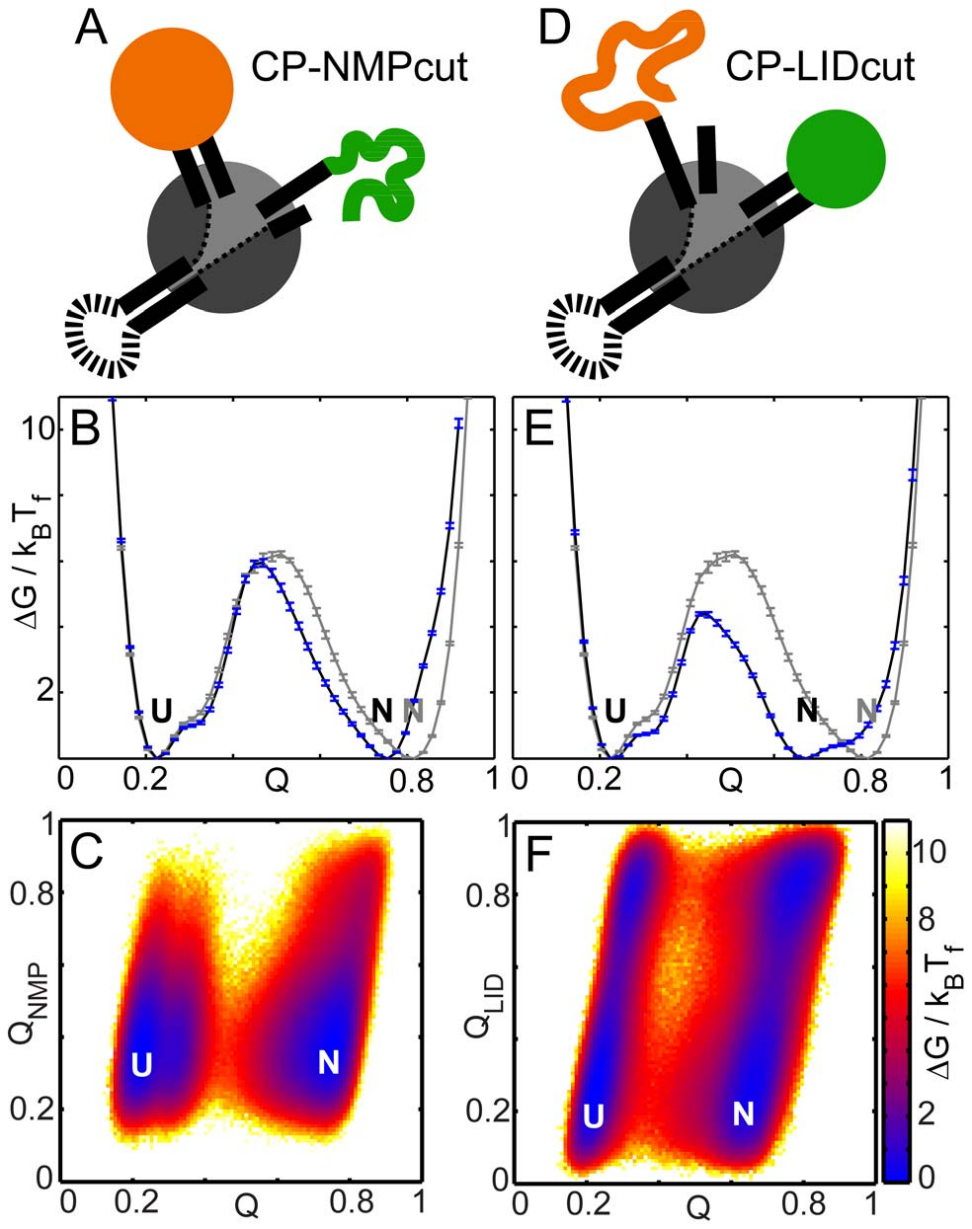
Folding of AKE circular permutants. All free energies are scaled by their respective k_B_T_f_s. N and U denote the native and the unfolded ensembles. The error bars represent twice the square root of the variance in the folding free energy and were calculated using a jackknife algorithm. (A) Cartoon of the folded state of CP-NMPcut at T_f_. (B) The FEP (black with blue error bars) shows a free energy barrier similar to that in WT (grey). The N ensemble shifts to lower Q as compared to WT. (C) The 2DFES plot with RCs of Q_NMP_ and Q. NMP does not fold completely in the N ensemble. (D) Cartoon of the folded state of CP-LIDcut at T_f_. (E) The FEP (black) shows that the barrier to folding is lower than that in WT AKE (grey). The N ensemble shifts to lower Q as compared to WT. (F) The 2DFES plot with RCs of Q_LID_ and Q shows a significantly higher population of unfolded LID in the N ensemble.

We computationally generate CPs of AKE by connecting the WT termini (Fig. 2B). New N- and C-termini are created before NMP in CP-NMPcut and before LID in CP-LIDcut (Fig. 2B, C). This converts the inserted NMP and LID domains into singly-linked domains in the CPs (Fig. 6A, D). The C-α SBMs for both CPs have the same native contacts as WT AKE renumbered according to their changes in topology (Fig. 1C, 2C). Two clusters of hydrophobic residues, one in NMP (V39, A49 and M53) and the other in the flanking helices of LID (I116, V117, V164 and L168) stabilize the two inserted domains [31]. The cuts in both CPs are before these clusters and do not disturb their connectivity. The native contacts from these clusters have the same stabilizing effect on NMP and LID in the CPs as in WT. Thus, any changes in folding cooperativity are only due to the conversion of NMP or LID to singly-linked domains.

On simulating CP-NMPcut, we find that its folding barrier is not significantly different from that of WT AKE (Fig. 6B). However, the folded ensemble has a single population at Q∼0.75 with NMP unfolded (N in Fig. 6C). The “foldedness” of CP-NMPcut gives how populated it is at the folded ensemble of WT-AKE (Q∼0.8) as compared to WT-AKE and equals exp(−ΔG_CP-NMPcut_(Q∼0.8)/k_B_T_f_)/exp(−ΔG_WT_(Q∼0.8)/k_B_T_f_) = exp(−1.6)/1∼0.202±0.006. The FEP of CP-LIDcut shows that the barrier height is ∼2 k_B_T lower than that in WT (Fig. 6E) and the folded basin has two populations. The less stable one at Q∼0.8 corresponds to completely folded CP-LIDcut, while the more stable population at Q∼0.65 has a partially folded protein with an unfolded LID domain (N in Fig. 6F). The foldedness of CP-LIDcut equals exp(−ΔG_CP-LIDcut_(Q∼0.8)/k_B_T_f_)/exp(−ΔG_WT_(Q∼0.8)/k_B_T_f_) = exp(−0.94)/1∼0.390±0.026. In both CPs, incomplete folding in the native state indicates lower folding cooperativity relative to WT. Together our results show that stabilizing interactions (here, appropriately placed native contacts due to a hydrophobic cluster) are insufficient to maintain folding cooperativity and that the insertion of the LID and NMP domains into the CORE domain is necessary. In the next section, we study the conformational transitions of AKE and its mutants to understand why domain insertion is preferred over strengthening inter-domain interactions for promoting cooperative folding.

### The mechanism of conformational transitions gets perturbed in ΔCORE-NMPi and ΔCORE-LIDi but is WT-like in the CPs

**Figure 7:**
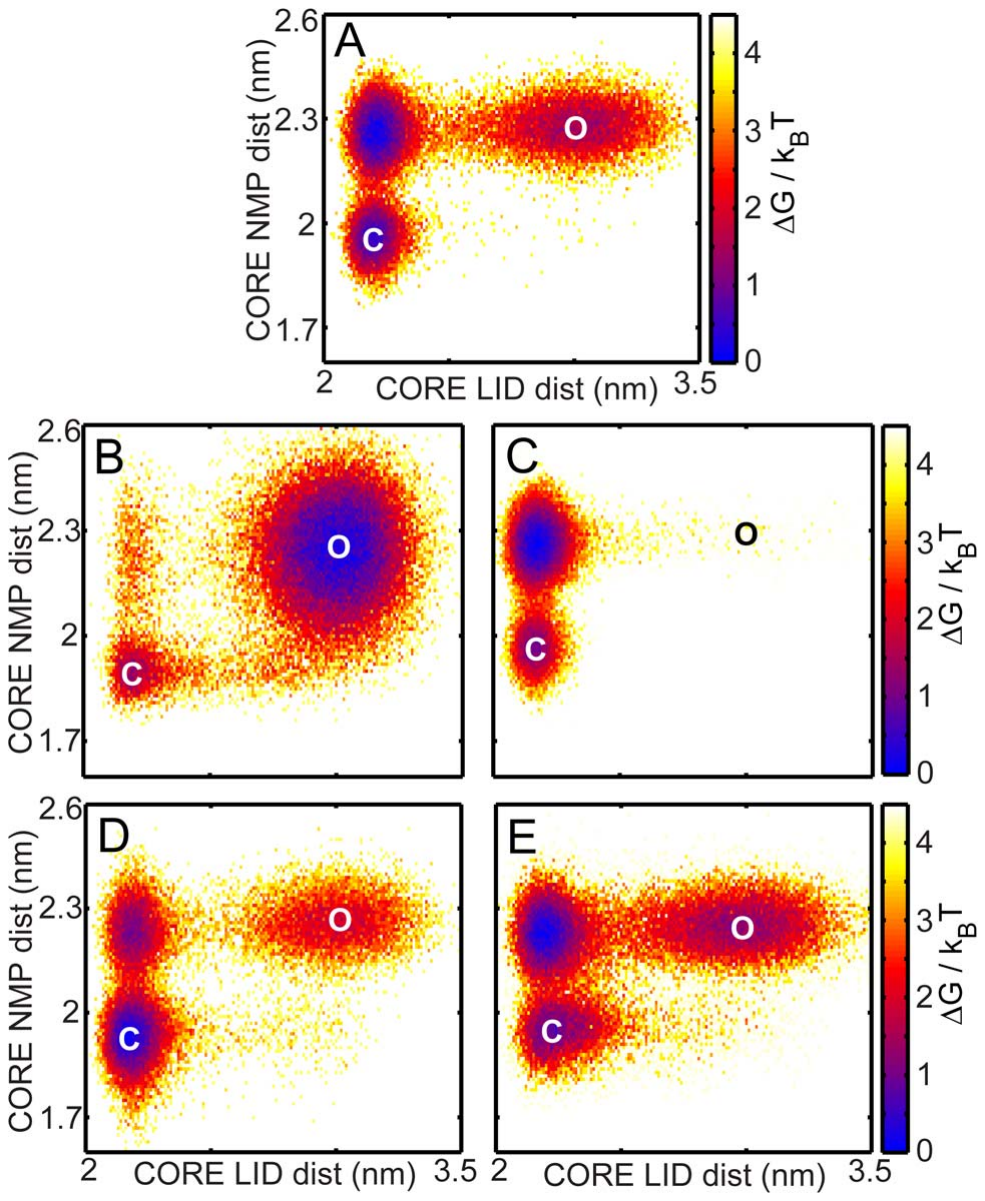
Conformational transitions of AKE and its mutants. All the conformational transition simulations are performed below T_f_ and the free energies are scaled by the simulation temperature (k_B_T). **O** is the open state and **C** is the closed state. The RCs used to obtain the conformational transition 2DFES are the distance between the centre of masses of CORE and LID residues (the CORE-LID dist) and the distance between the centre of masses of CORE and NMP (termed the CORE-NMP dist). (A) The conformational transition 2DFES for WT AKE shows that LID closes before NMP. (B) The ΔCORE-NMPi 2DFES shows that conformational transitions proceed mainly through a LID-open-NMP-closed state unlike WT. (C) In ΔCORE-LIDi, the mechanism of the transitions remains the same as in WT. However, the open state is very sparsely populated. (D) In CP-NMPcut and (E) in CP-LIDcut, the conformational transitions show a mechanism similar to that in WT AKE with most transitions occurring via the LID-closed-NMP-open ensemble.

We perform conformational transition simulations of AKE using a previously developed dual-SBM [18], [19] whose open state is the same structure as that used to simulate folding. The closed state is introduced into this C-α SBM through the addition of 39 contacts (Fig. 1C, red contacts) whose minimum energy (native contact) distances are calculated from the ligand-bound structure, 1AKE.pdb, chain A [18]. The strength of these closed state specific contacts is tuned to populate both the open and closed state ensembles in each of the proteins (Table S1). The molecular mechanism of the conformational transition of WT AKE has a LID-closed NMP-open intermediate and its relevance to experiment has already been shown [19]. Here, we reproduce the WT results (Fig. 7A) and use the same dual-SBM to understand how perturbations to either domain interfaces or chain connectivity affect the mechanism of conformational transitions. All conformational transition simulations are performed below T_f_ where the proteins do not unfold (See SI Methods and Table S1 for details). Also, at these temperatures the CPs of AKE are fully folded.

Unlike in WT AKE, the main route for the conformational transitions in ΔCORE-NMPi is through a LID-open-NMP-closed intermediate (Fig. 7B). It has been suggested that the mechanism of conformational transitions is important both for preventing misligation during the binding of ATP and AMP and for maintaining the high catalytic efficiency of WT AKE [19], [21]. The deletion of the CORE-NMP interface contacts completely alters the mechanism of conformational transitions. The main route for the conformational transitions in ΔCORE-LIDi remains WT-like (Fig. 7C). However, removing the CORE-LID interface reduces the population of the open state significantly and this is also likely to increase misligation [19].We conclude that the inter-domain interfaces are not necessary for the cooperative folding of WT AKE but are tuned to achieve the correct mechanism of conformational transitions.

We next simulate the conformational transitions of both CPs (Fig. 7D, E) and find that the population of the three states and the overall mechanism of conformational transitions are largely WT-like. Perturbing the edges of the inserted domains by making them singly-connected marginally affects the populations of the states. Thus, the choice of domain connectivity in AKE (with or without inserted NMP and LID domains) does not affect the mechanism of conformational transitions. We conclude that domain insertion is used to promote folding cooperativity in AKE because domain interfaces are tuned to facilitate conformational transitions.

## Discussion

### Domain insertion can stabilize the inserted domain and increase folding cooperativity

Domain insertion constrains the position of the N- and C-termini of the inserted domain when the discontinuous domain (e.g. CORE is the discontinuous domain in AKE; Fig. 1B) is folded. This reduces the conformational entropy of the inserted domain and stabilizes its folded state. Thus, domain insertion is likely to be advantageous for small, marginally stable domains such as NMP and LID during instances of spontaneous local unfolding in the cell [6]. Evidence that the folded states of small domains are more stable upon insertion into a stably folded discontinuous domain also comes from experiments on the *E. coli* protein slyD. slyD consists of an unfolded polypeptide binding domain inserted into a proline isomerisation catalyzing FKBP domain [37]. The insert is unfolded in isolation but becomes structured within the slyD FKBP domain [37].

Inserting a smaller domain with fewer stabilizing interactions and a lower T_f_, into a larger, more stable one (with a higher T_f_) not only stabilizes the inserted domain but also couples their folding and brings their T_f_s closer to each other [36]. This allows the entire protein to fold more cooperatively. In addition to the present simulations of AKE, reduced folding cooperativity upon the conversion of an inserted domain into a singly-linked one has also been observed in folding experiments on T4-lysozyme (T4L) [38].

**Figure 8:**
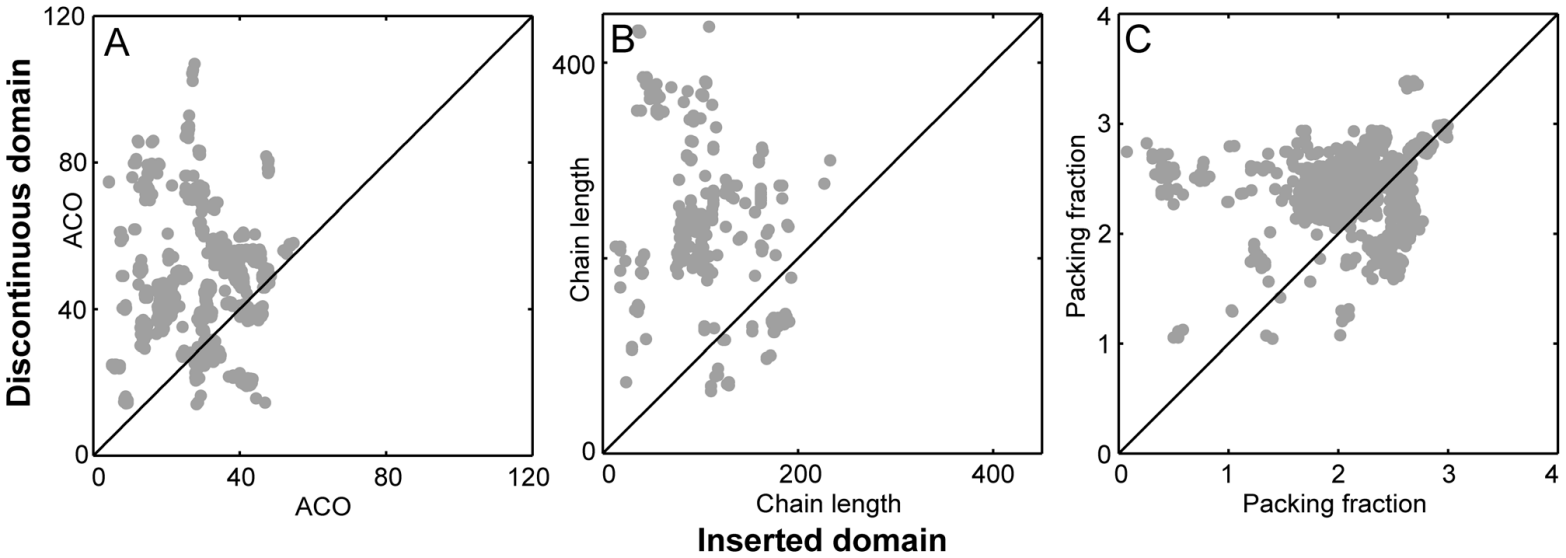
Absolute contact order (ACO), chain length and packing fraction of insert-discontinuous domain pairs from the Pfam-A database. We extract 1713 protein chains from Pfam which have an inserted domain and an associated PDB ID and a chainID. For each chain the (A) ACO, (B) the chain length and (C) the packing fraction of the inserted ( = x) and the discontinuous domains ( = y) are calculated separately and marked as filled grey circles at the (x, y) point. The ACO, chain length and packing fraction for a majority of the insert-discontinuous domain pairs lie above the y = x line (marked in black in A–C). Fig. S6 represents the same data coloured according to the Pfam family of the inserted domain and corroborates this inference at the Pfam family level.

We extract the structures of diverse families of multi-domain proteins with inserts (36 inserted domain families in total) from the Pfam database [39] and calculate three structural parameters, which have previously been correlated with the folding properties of single domain proteins (Fig. 8). For each protein chain (there are a total of 1713 chains), we compare the structural parameters of the inserted domain to those of the discontinuous domain in order to understand if domain insertion can stabilize the inserted domain and increase folding cooperativity in other multi-domain proteins. Further details of the data collation and analysis are given in the SI Methods.

The structural parameters that we calculate separately for the inserts and the discontinuous domains of each protein chain are the absolute contact order (ACO) [11], the chain length and the packing fraction. The ACO and to a lesser extent the chain length inversely correlate with the folding rate of a protein domain [40] and reflect its kinetic stability and its resistance to spontaneous unfolding. The chain length (through its correlation with protein stability) and the packing fraction may correlate with the T_f_ (or the thermodynamic stability) of individual domains [41]. In Fig. 8, we plot the ACO (Fig. 8A), the chain length (Fig. 8B) and the packing fraction (Fig. 8C) of the discontinuous domain versus that of the corresponding inserted domain. (Also see Fig. S6 for a family-wise split of the data).

The ACO (Fig. 8A) is the average of the number of residues along the protein chain that separate a pair of residues that form a native contact [11]. We use the same contact calculation for these protein chains as we use for C-α SBMs. ACO assesses the complexity of a fold and has been shown to be inversely correlated with the logarithm of protein folding rates [40]. Although unfolding rates have not been directly correlated with ACO, slow folding rates imply a high barrier to folding (and unfolding) at T_f_ [42] and in turn slow unfolding rates. Slow unfolding rates will reduce the events of spontaneous unfolding once a protein domain is folded. We find that the discontinuous domains of 1343 out of the 1713 protein chains have a larger ACO than their inserted domains. Further, the ACO of the inserted domain of every member of 31 out of the 36 Pfam families is lower than that of the corresponding discontinuous domain. This data and the following data on chain length indicate that discontinuous domains are on an average, more kinetically stable and more resistant to unfolding than the inserted domains. As in AKE and slyD, insertion of a kinetically less stable insert is likely to stabilize its folded state once the discontinuous domain is folded.

Thermodynamic stabilities of proteins can be calculated from their chain lengths [41] and longer domains are expected to be more stable than shorter ones. The square root of the chain length also inversely correlates with the logarithm of the folding rate [40]. We find that a greater number of protein chains (1318 out of 1713 and 32 out of 36 Pfam families) have longer discontinuous domains than inserted domains (Fig. 8B). An earlier bioinformatics study with a different data set also showed the same trend [43].

Finally, we calculate and plot the packing fraction (Fig. 8C). This is the ratio of the number of contacts to the number of residues in a protein. It varies between 2.0 and 3.0 when the contacts are coarse-grained to a C-α level as in our simulation models [12]. There is evidence from studies on thermophilic proteins that a higher packing fraction (and consequently better packing) can lead to a higher T_f_ [44]. We find that again that a larger number of discontinuous domains (1175 out of 1713 and 21 out of 36 families) have a higher packing fraction than their respective inserted domains (Fig. 8C). Besides the fact that quantities such as ACO, chain length and packing fraction can only establish trends and may not accurately predict the folding of individual domains and proteins, there are several other caveats to our dataset and our analyses which may bias the above results and we list some of these in SI Methods.

The overall trend that emerges is that the T_f_s, the thermodynamic and the kinetic stabilities of the discontinuous domains are larger than those of their respective inserted domains (1079 of the 1713 or 63% of the protein chains have all three i.e. ACO, chain length and packing fraction higher in the discontinuous domain than in the inserted domain). Thus, it is likely that domain insertion stabilizes the folded state and increases the folding cooperativity of at least some other multi-domain proteins with inserts. Further, both our folding simulations and the above structural bioinformatics analysis indicate that the design of singly-linked multi-domain proteins is likely to be fundamentally different from that of proteins with inserts. Thus, studies that investigate the connection between the folding and evolution of multi-domain proteins [6], [45] should classify proteins based on their chain topology. We next discuss the functional advantages of domain insertion.

### Domain insertion can maintain folding cooperativity even when inter-domain interfaces are tuned for function

Domain insertion naturally couples the folding of the inserted and the discontinuous domains of a protein and can, given the right order of stabilities of domains, make folding cooperative. However, domain insertion also makes the topology of the protein more complex and is likely to slow down its folding [11]. Further, it is also possible for singly-linked, multi-domain proteins to fold cooperatively with appropriately tuned inter-domain interactions [1], [6]–[10]. Given the presence of inserted domains in several multi-domain proteins [43], we would like to know if domain insertion confers an additional advantage onto a multi-domain protein which is absent when the same two domains are singly-linked. Here we argue, using AKE, T4L and slyD as examples, that domain insertion may be advantageous because it can promote folding cooperativity while accommodating the constraints imposed on domain interfaces by protein function. In AKE, the area of interaction between domains is small and the interface residues are selected to bind ATP and AMP and to locally unfold and promote conformational transitions [14]–[16], [18]. The residues at the domain interface of T4L are tuned to bind and hydrolyze bacterial cell wall peptidoglycans [46]. slyD has almost no inter-domain interface, likely because the insert needs to be mobile in order to bind unfolded polypeptide chains using a fly casting-like mechanism [47]. Despite these functional constraints, AKE, T4L and slyD can fold cooperatively due to their inserted domain topologies.

When the area of interaction between domains is small or almost non-existent, it may not be possible to tune inter-domain interactions for anything other than function and with domain insertion, these interactions are not necessary to promote folding cooperativity. If the area of interaction between the domains is large, it may be possible to promote folding cooperativity using only inter-domain interactions [10] even when interfaces are functional. In such cases, domain insertion may not confer any extra advantage.

In order to understand if other multi-domain proteins with inserts have function at inter-domain interfaces, we use the 36 inserted domain Pfam families (1713 protein chains). We further classify these families into 38 sets of proteins such that the proteins within each set have the same pair of Pfam IDs for the inserted and the discontinuous domains. We use the function annotation database, Firedb [48], to output groups of residues which are part of individual functional sites in the 1713 protein chains. We then identify chains with functional sites that span the inserted and the discontinuous domains and mark them as having function at the interface. We find that 24 out of the 38 sets of proteins have at least one protein chain that has function at the domain interface. If we assume that similar inserted and discontinuous domain pairs have similar function then ∼63% of the protein sets have functional domain interfaces. We note in passing that 14 out of the 24 sets of protein chains with interface function have higher ACO, chain length and packing fraction in their discontinuous domain than in their inserted domain. It has been hypothesized that the interfaces of multi-domain proteins (whether they be singly-linked or with inserted domains) are enriched in function [49]. A study of whether the inter-domain interfaces of multi-domain proteins with inserts are more enriched in function than those of singly-linked multi-domain proteins is currently ongoing in our group.

### Possible connections to studies on protein evolution and design

A recent study [50] discusses sequence and structure divergence in the HAD superfamily of proteins. Insertion of different domains into a Rossmann discontinuous domain imparts specificity to the enzymatic activity of proteins within the HAD superfamily. The inter-domain interface seems to be critical for catalysis and its structure is conserved across the HAD proteins. However, correlated structural changes between the two domains are observed far from the domain interface. Although little is known about the folding of these proteins, we suggest that this structural co-evolution of the domains may also compensate for the differences in size and stability of the inserts and promote folding cooperativity.

Except for the antagonistic coupling between the folding of the inserted and discontinuous domains in protein switches [51], the design of multi-domain proteins rarely accounts for folding cooperativity [51]–[53]. Several proteins with inserts have been designed to couple the functions of two domains [51]. Cooperative folding may be advantageous in such proteins and our study suggests that at the least, the less stable domain should be inserted into the more stable one.

### Conclusions

Based on our simulations of AKE, we conclude that the insertion of a smaller and less stable domain into a larger and more stable one promotes folding cooperativity. Given that folding cooperativity is hypothesized to have evolved to reduce misfolding and aggregation, we expect inserted domains to be an important motif in multi-domain proteins. Further, domain insertion (and the resulting protein topology) allows the residues that promote folding cooperativity to be separate from the residues which are required for function. Using structural analysis of several multi-domain protein families, we hypothesize that domain insertion may promote folding cooperativity in at least some other multi-domain proteins whose interfaces are tuned to facilitate substrate binding, conformational transitions or domain mobility and we hope that future experimental studies will investigate this aspect of the folding of multi-domain proteins with inserts.

## Methods

The details of the C-α SBMs [34] of WT AKE and its topological variants are given either in the Results section or in SI Methods. Contact lists were identified using CSU analysis [54] and are given in SI Text S3. MODELLER 9 [55] was used to create all poly-glycine loops added to the CPs of AKE. All simulations were performed using GROMACS 4.0 [56] (See SI Methods for details). We define the fraction of native contacts (Q) using a smooth, switching function (Section 4.19 of the Ref [57]) and rescale it such that Q equals 0 when no contacts are formed and 1 when all contacts are formed (SI Methods Eq. S2, S3).

## Supporting Information

Figure S1 **Clusters of native contacts from kinetic simulations of WT AKE.** (A) The CLANS visualization of the three contact clusters. The two clusters on the right are shown in the inset. (B) The native contacts that belong to each of the clusters shown in A are marked. X and Y axes represent residue number. Intra helical contacts have inherently low cross-correlation coefficients and are not part of any cluster. However, their tertiary contacts are present in the clusters, and therefore, their residues are included in the corresponding cluster. By comparing the contact clusters with the domain definitions we identify the three clusters as: LID (orange, residues 118–160), CORE-N (black, residues 1–29 and 68–117) and CORE-C (grey, residues 161–214).
(TIF)

Figure S2 **Free energy profiles and 2DFESs of WT AKE and its topological variants at their T_f_s.** Rows: (A) WT (B) ΔCORE-NMPi (C) ΔCORE-LIDi (D) CP-NMPcut, and (E) CP-LIDcut. Columns: (1^st^) FEP as a function of Q (black with blue error bars, WT is in grey in B–E) with error bars representing twice the square root of the variance, (2^nd^) 2DFES with RCs, Q_CORE_ and Q, (3^rd^) 2DFES with RCs, Q_CORE-N_ and Q_CORE-C_ (4^th^) 2DFES with RCs, Q_LID_ and Q, and (5^th^) 2DFES with RCs, Q_NMP_ and Q. In (B, C), the scaled RCs, Q*, Q*_NMP_, Q*_LID_, Q*_CORE_, Q*_CORE-N_, and Q*_CORE-C_ are used as applicable and are defined in the Supporting Methods. Some of these 2DFESs are shown in Figs. 3, 5 and 6. The free energy in all plots is scaled by their respective k_B_T_f_s and is dimensionless.
(TIF)

Figure S3 **Ratio of the van't Hoff to calorimetric entalpies for WT AKE and its topological variants.** Folding cooperativity is well estimated by the ratio of the van't Hoff to calorimetric enthalpies. A ratio of 1 indicates cooperative folding and a ratio of 0 indicates non-cooperative folding. These ratios are computed from the equilibrium simulations of WT and the mutants of AKE at their respective T_f_s. The error bars represent twice the square root of the variance. WT AKE has the highest cooperativity followed by ΔCORE-NMPi and ΔCORE-LIDi. The CPs, CP-NMPcut and CP-LIDcut have lower folding cooperativity as compared to WT.
(TIF)

Figure S4
**A typical simulation trajectory and free energy convergence for WT AKE.** (A) A time trace of the fraction of native contacts (Q) from a representative simulation of WT AKE at its estimated T_f_. This trace shows transitions between the folded (Q∼0.8) and unfolded ensembles (Q∼0.2). (B) We plot the evolution of the free energy profile with increasing simulation time. The bottom panel shows the location of folding (U to N) or unfolding (N to U) transitions at the time that they occurred in the simulation trajectory. 29 such transitions were observed for WT AKE. The middle panel plots the difference (as given by Eq. S8) between the free energy profile, *F(Q,i,)*, calculated by using simulation data up to the time point *t_i_*, and the final free energy profile (calculated using all the data, i.e. upto *t_n_*). The intermediate free energy profiles, *F(Q,i)*, were first reweighted to their respective folding temperatures, T_f_(i)'s before the difference was calculated. All *F(Q,i)* are in scaled by their respective k_B_T_f_(i). The T_f_(i)'s are plotted in the top panel. The middle and the top panels show that the free energy profile and the T_f_ have converged by the end of the simulation.
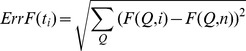
(Eq.S8)
(TIF)

Figure S5 **2DFESs of inter-domain interface contacts in WT AKE.** (A) Q_CORE-NMPi_ vs. Q shows that the CORE-NMP interface is significantly formed in the transition state (the red population at Q∼0.5, Q_CORE-NMPi_∼0.8). The formation of the CORE-NMP interface in the transition state is consistent with its inclusion in CORE-N (formed in the transition state, Fig. 3G). (B) Q_CORE-LIDi_ vs. Q shows that the CORE-LID interface is not completely formed in the transition state (the red population at Q∼0.5, Q_CORE-LIDi_∼0.4).
(TIF)

Figure S6 **A family-wise comparison of the structural features of the inserted and the discontinuous domains.** Domains from 36 inserted domain Pfam families are found to be inserted in the structures of multi-domain proteins present in the PDB. For each such multi-domain protein chain from the PDB, (A–C) the absolute contact order (ACO), (D–F) the chain length and (G–I) the packing fraction are plotted for the discontinuous vs. the inserted domains. The plot of every such chain is shown in Fig. 8. Here we split the 36 families into 3 groups of 12 and plot their structural parameters in separate columns (A,D,G correspond to families 1–12, B,E,H correspond to families 13–24 and C,F,I correspond to families 25–36). The numbering of the families is arbitrary and the data has been split only to aid in visualization of different families. Within a column the same color denotes the same Pfam family. The y = x line is also plotted. Points above this line have a higher value for the structural parameter of the discontinuous domain than the inserted domain. Proteins within the same family usually cluster together.
(TIF)

Table S1 **Temperatures and the energetic scaling factors for equilibrium simulations.** Error on the T_f_ estimates is ±0.1 K or 0.0008314 (k_B_T units).
(PDF)

Table S2 **Summary of the experimental results obtained from refolding kinetics of AKE compared with folding simulations of WT AKE (See** **Fig. 4** **).**
(PDF)

Text S1 **Supporting methods.**
(PDF)

Text S2 **Supporting results.**
(PDF)

Text S3 **Additional supporting information.** Contact lists for WT AKE, some of which are used to define the reaction coordinates (RCs).
(DOCX)

Text S4 **List of all PDBs having insert-discontinuous domain pairs used in the structural bioinformatics analysis.**
(DOCX)

Text S5 **Supporting references.**
(PDF)
