# Supplementary figures and images for: In the Multi-domain Protein Adenylate Kinase, Domain Insertion Facilitates Cooperative Folding while Accommodating Function at Domain Interfaces
Source: PLoS Comput Biol. 2014 Nov 13;10(11):e1003938. doi: 10.1371/journal.pcbi.1003938 (PMC4230728; doi:10.1371/journal.pcbi.1003938)

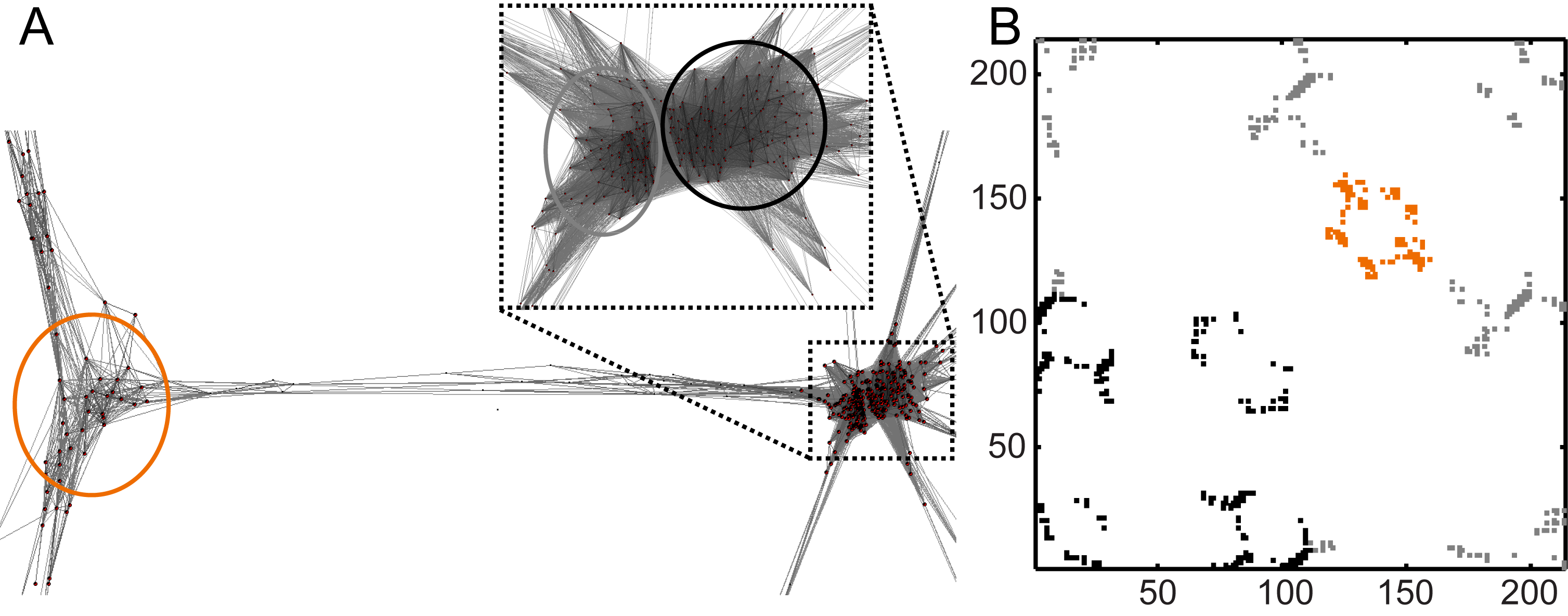

Supplement: Figure S1 — Clusters of native contacts from kinetic simulations of WT AKE. (A) The CLANS visualization of the three contact clusters. The two clusters on the right are shown in the inset. (B) The native contacts that belong to each of the clusters shown in A are marked. X and Y axes represent residue number. Intra helical contacts have inherently low cross-correlation coefficients and are not part of any cluster. However, their tertiary contacts are present in the clusters, and therefore, their residues are included in the corresponding cluster. By comparing the contact clusters with the domain definitions we identify the three clusters as: LID (orange, residues 118–160), CORE-N (black, residues 1–29 and 68–117) and CORE-C (grey, residues 161–214). (TIF) [file pcbi.1003938.s001.tif]

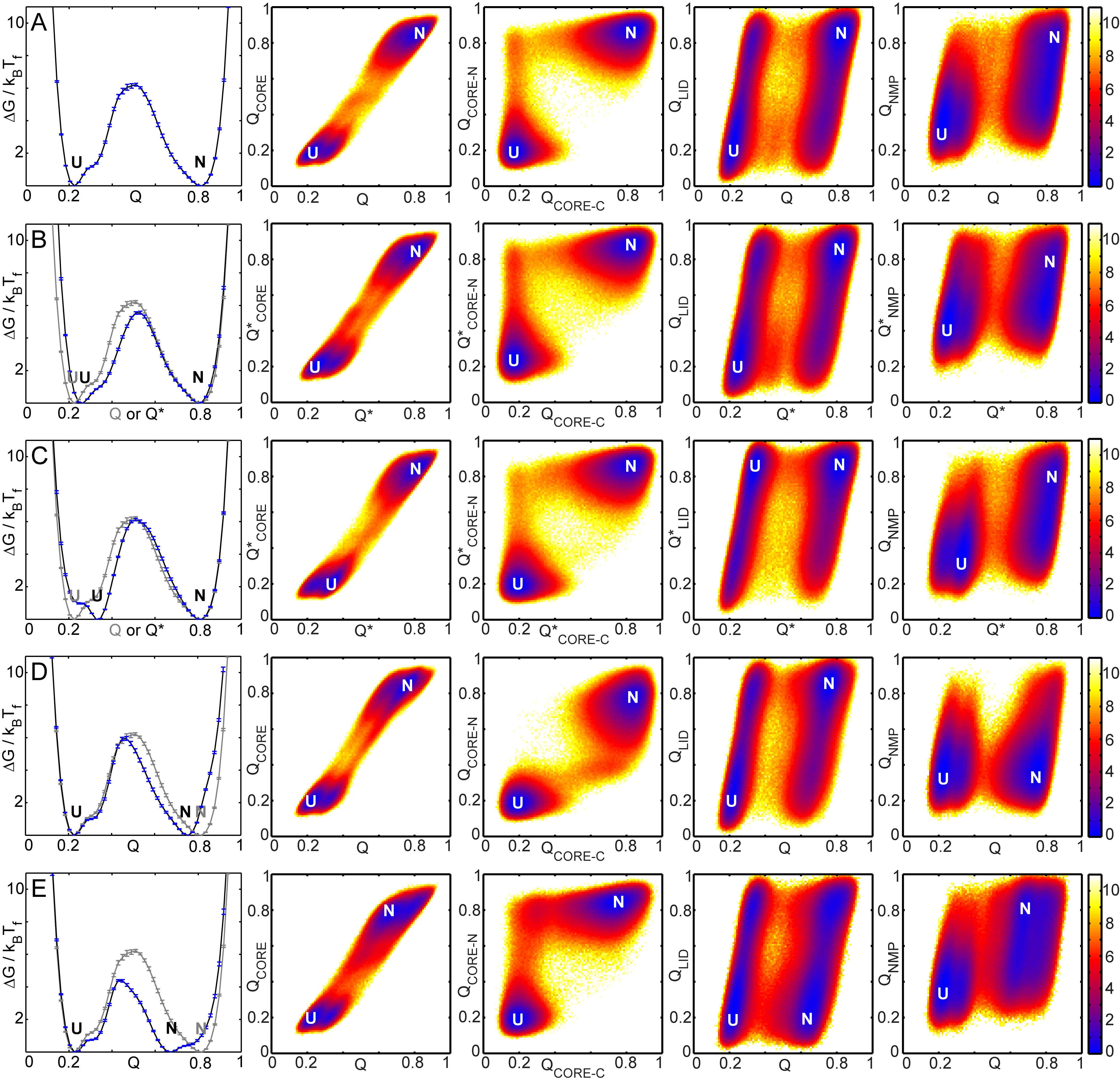

Supplement: Figure S2 — Free energy profiles and 2DFESs of WT AKE and its topological variants at their Tfs. Rows: (A) WT (B) ΔCORE-NMPi (C) ΔCORE-LIDi (D) CP-NMPcut, and (E) CP-LIDcut. Columns: (1st) FEP as a function of Q (black with blue error bars, WT is in grey in B–E) with error bars representing twice the square root of the variance, (2nd) 2DFES with RCs, QCORE and Q, (3rd) 2DFES with RCs, QCORE-N and QCORE-C (4th) 2DFES with RCs, QLID and Q, and (5th) 2DFES with RCs, QNMP and Q. In (B, C), the scaled RCs, Q*, Q*NMP, Q*LID, Q*CORE, Q*CORE-N, and Q*CORE-C are used as applicable and are defined in the Supporting Methods. Some of these 2DFESs are shown in Figs. 3, 5 and 6. The free energy in all plots is scaled by their respective kBTfs and is dimensionless. (TIF) [file pcbi.1003938.s002.tif]

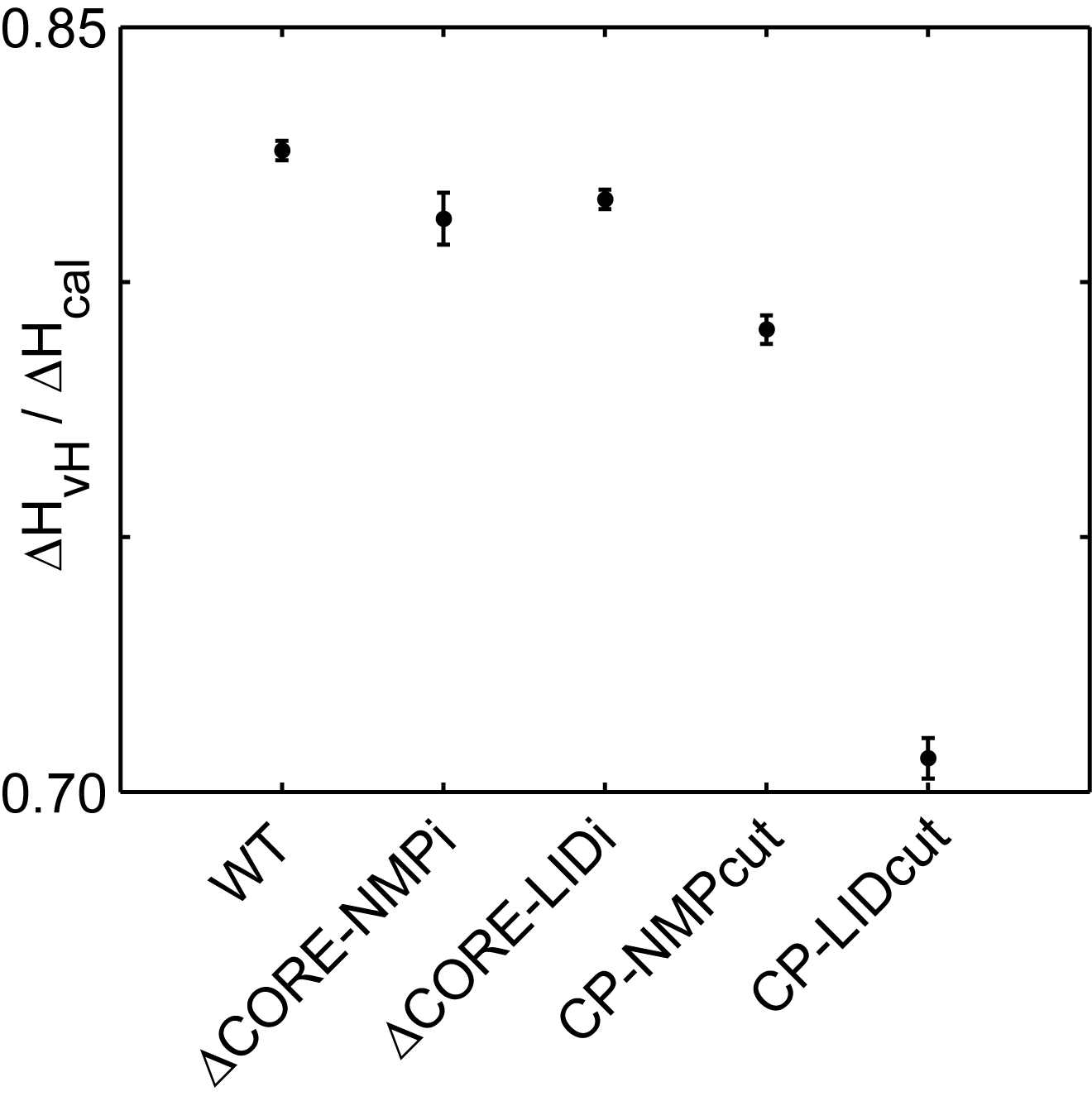

Supplement: Figure S3 — Ratio of the van't Hoff to calorimetric entalpies for WT AKE and its topological variants. Folding cooperativity is well estimated by the ratio of the van't Hoff to calorimetric enthalpies. A ratio of 1 indicates cooperative folding and a ratio of 0 indicates non-cooperative folding. These ratios are computed from the equilibrium simulations of WT and the mutants of AKE at their respective Tfs. The error bars represent twice the square root of the variance. WT AKE has the highest cooperativity followed by ΔCORE-NMPi and ΔCORE-LIDi. The CPs, CP-NMPcut and CP-LIDcut have lower folding cooperativity as compared to WT. (TIF) [file pcbi.1003938.s003.tif]

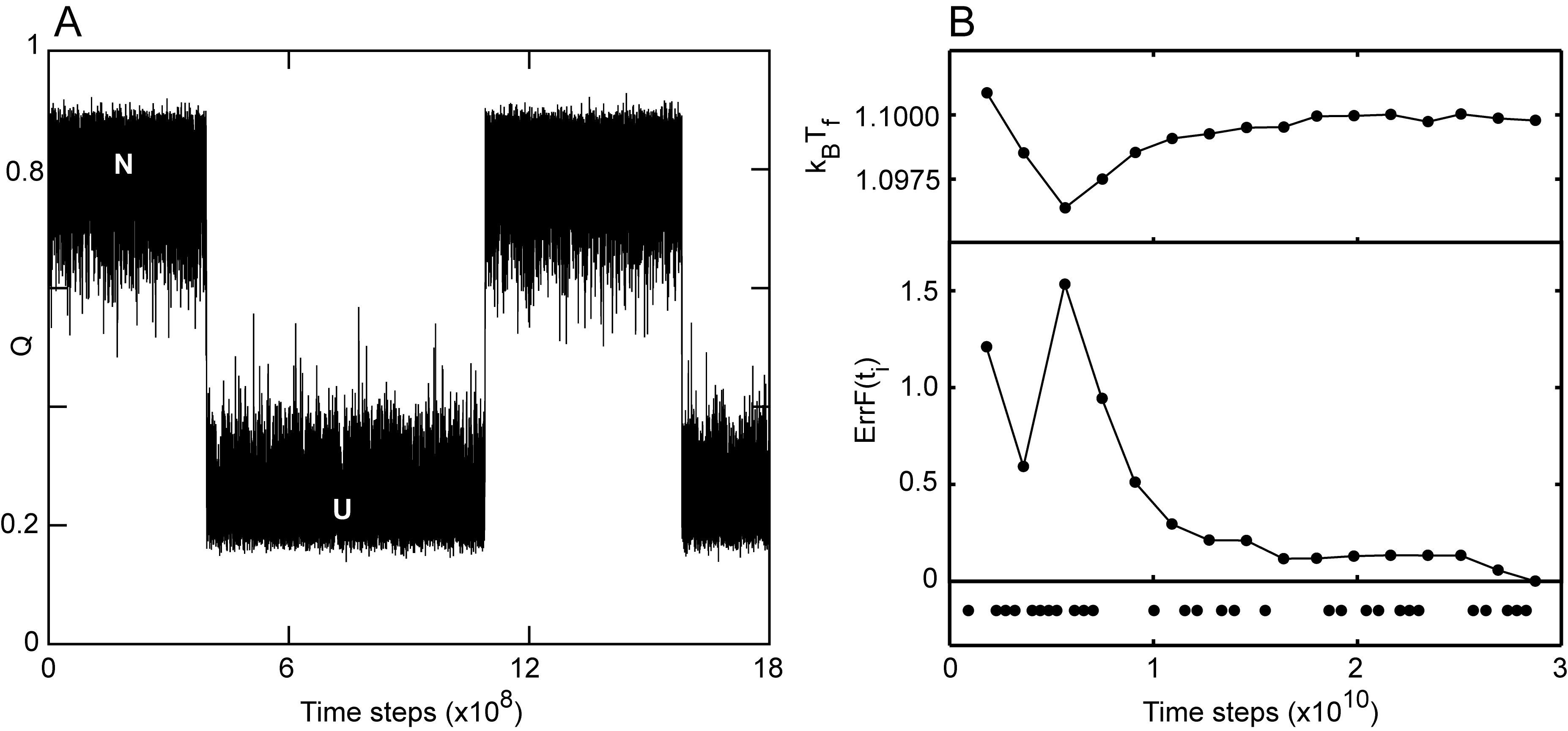

Supplement: Figure S4 — A typical simulation trajectory and free energy convergence for WT AKE. (A) A time trace of the fraction of native contacts (Q) from a representative simulation of WT AKE at its estimated Tf. This trace shows transitions between the folded (Q∼0.8) and unfolded ensembles (Q∼0.2). (B) We plot the evolution of the free energy profile with increasing simulation time. The bottom panel shows the location of folding (U to N) or unfolding (N to U) transitions at the time that they occurred in the simulation trajectory. 29 such transitions were observed for WT AKE. The middle panel plots the difference (as given by Eq. S8) between the free energy profile, F(Q,i,), calculated by using simulation data up to the time point ti, and the final free energy profile (calculated using all the data, i.e. upto tn). The intermediate free energy profiles, F(Q,i), were first reweighted to their respective folding temperatures, Tf(i)'s before the difference was calculated. All F(Q,i) are in scaled by their respective kBTf(i). The Tf(i)'s are plotted in the top panel. The middle and the top panels show that the free energy profile and the Tf have converged by the end of the simulation.(Eq.S8) (TIF) [file pcbi.1003938.s004.tif]

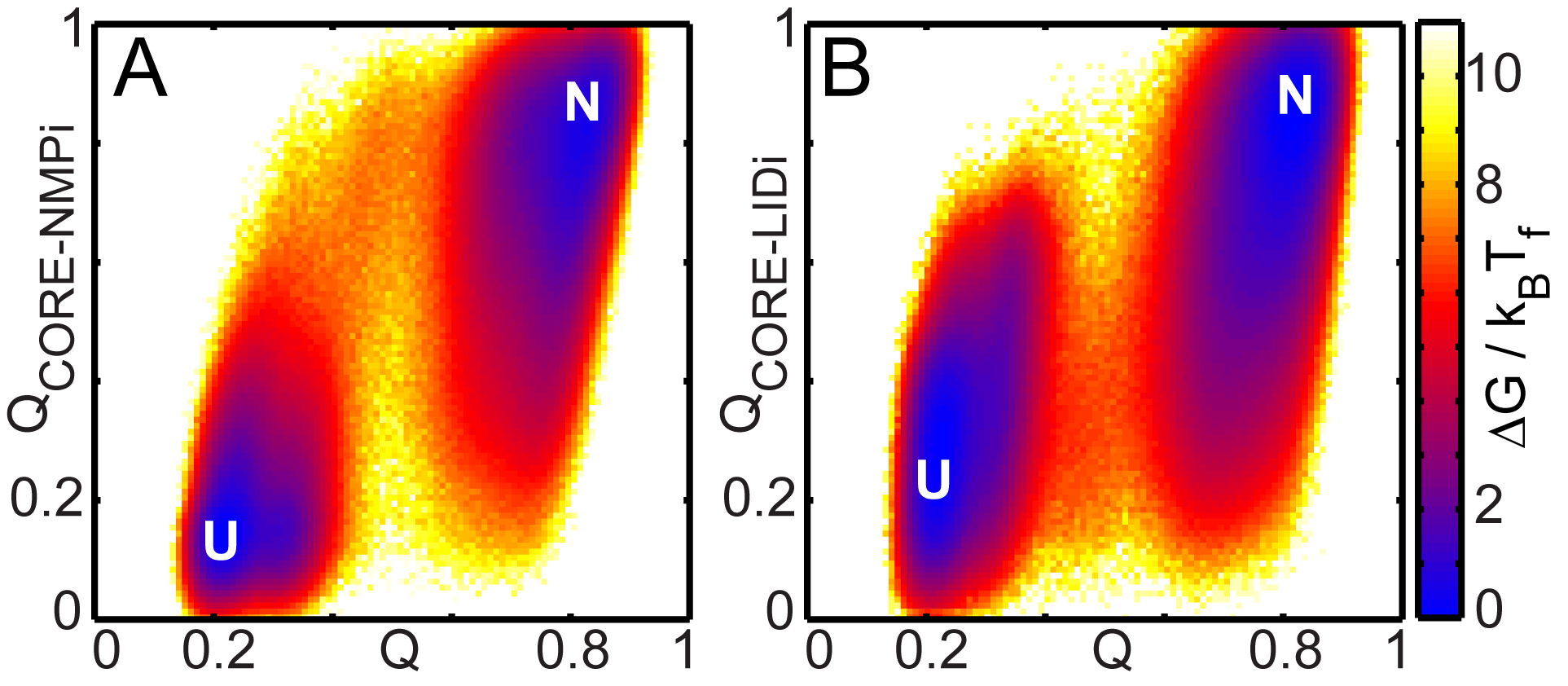

Supplement: Figure S5 — 2DFESs of inter-domain interface contacts in WT AKE. (A) QCORE-NMPi vs. Q shows that the CORE-NMP interface is significantly formed in the transition state (the red population at Q∼0.5, QCORE-NMPi∼0.8). The formation of the CORE-NMP interface in the transition state is consistent with its inclusion in CORE-N (formed in the transition state, Fig. 3G). (B) QCORE-LIDi vs. Q shows that the CORE-LID interface is not completely formed in the transition state (the red population at Q∼0.5, QCORE-LIDi∼0.4). (TIF) [file pcbi.1003938.s005.tif]

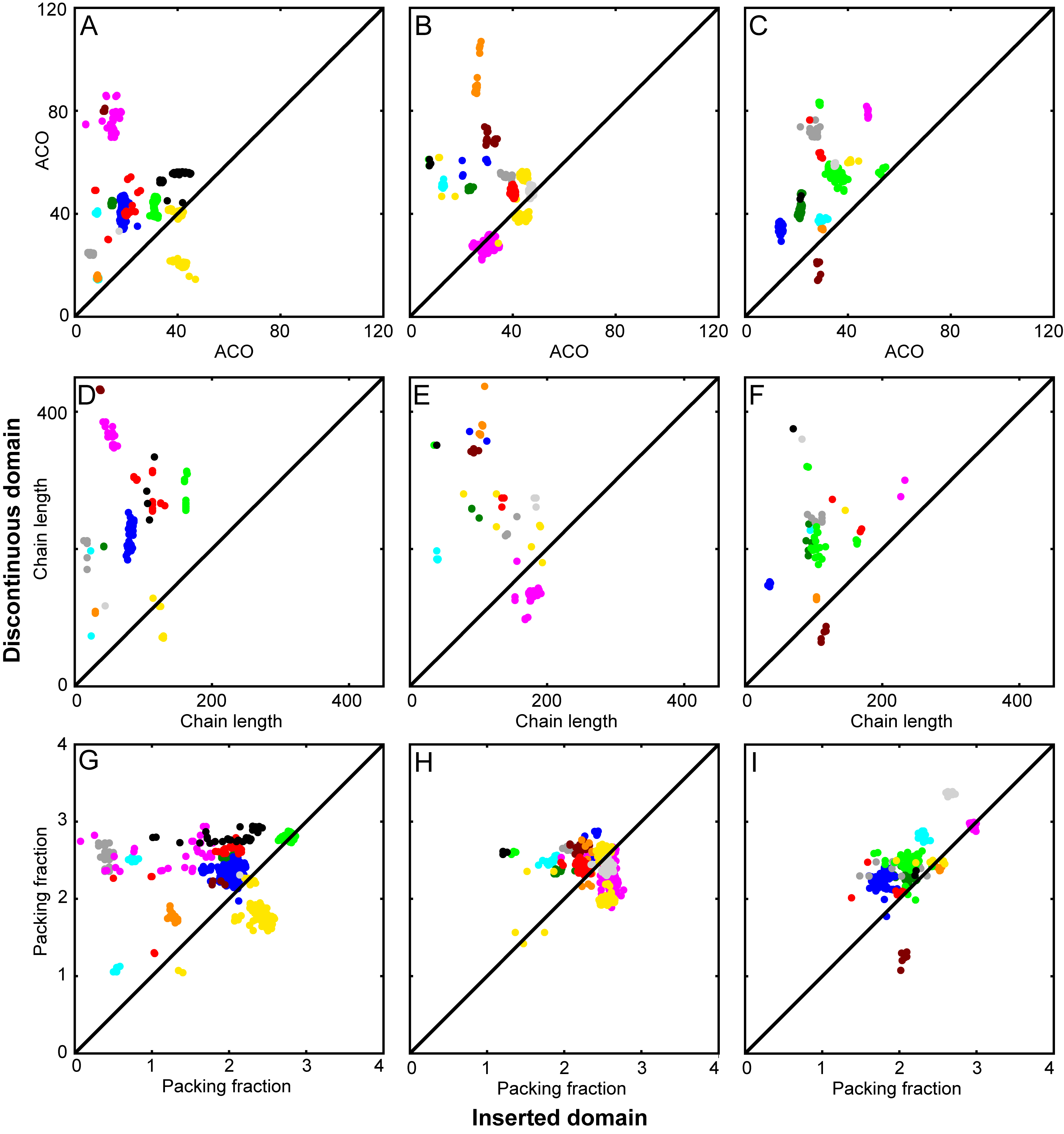

Supplement: Figure S6 — A family-wise comparison of the structural features of the inserted and the discontinuous domains. Domains from 36 inserted domain Pfam families are found to be inserted in the structures of multi-domain proteins present in the PDB. For each such multi-domain protein chain from the PDB, (A–C) the absolute contact order (ACO), (D–F) the chain length and (G–I) the packing fraction are plotted for the discontinuous vs. the inserted domains. The plot of every such chain is shown in Fig. 8. Here we split the 36 families into 3 groups of 12 and plot their structural parameters in separate columns (A,D,G correspond to families 1–12, B,E,H correspond to families 13–24 and C,F,I correspond to families 25–36). The numbering of the families is arbitrary and the data has been split only to aid in visualization of different families. Within a column the same color denotes the same Pfam family. The y = x line is also plotted. Points above this line have a higher value for the structural parameter of the discontinuous domain than the inserted domain. Proteins within the same family usually cluster together. (TIF) [file pcbi.1003938.s006.tif]
