# Supplementary material for: In the Multi-domain Protein Adenylate Kinase, Domain Insertion Facilitates Cooperative Folding while Accommodating Function at Domain Interfaces
Source: PLoS Comput Biol. 2014 Nov 13;10(11):e1003938. doi: 10.1371/journal.pcbi.1003938 (PMC4230728; doi:10.1371/journal.pcbi.1003938)
Supplement: Table S1 — Temperatures and the energetic scaling factors for equilibrium simulations. Error on the Tf estimates is ±0.1 K or 0.0008314 (kBT units). (PDF) [file pcbi.1003938.s007.pdf]

WT or Mutant	Simulation temperature. (k_BT)	Folding temperature (k_BT_f)	Temperature for conformational transitions (k_BT)	Energy of closed state specific contacts i.e. scaling factor ε_3
WT	1.0975	1.0997	0.8980	1.2
ΔCORE-NMPi	1.0975	1.0991	0.6652	0.9
ΔCORE-LIDi	1.1058	1.1048	0.8980	1.2
CP-NMPcut	1.0892	1.0917	0.8980	1.2
CP-LIDcut	1.0892	1.0886	0.8980	1.1
pCP	1.0393	1.0382	0.8980	1.1

Table S1. Temperatures and the energetic scaling factors for equilibrium simulations of WT AKE and its mutants. Error on the T_f estimates is ± 0.1 K or 0.0008314 (k_BT units).
