# Supplementary material for: In the Multi-domain Protein Adenylate Kinase, Domain Insertion Facilitates Cooperative Folding while Accommodating Function at Domain Interfaces
Source: PLoS Comput Biol. 2014 Nov 13;10(11):e1003938. doi: 10.1371/journal.pcbi.1003938 (PMC4230728; doi:10.1371/journal.pcbi.1003938)
Supplement: Table S2 — Summary of the experimental results obtained from refolding kinetics of AKE compared with folding simulations of WT AKE (See Fig. 4 ). (PDF) [file pcbi.1003938.s008.pdf]

Residue position of single Tryptophan	Location reported on	Formation of native structure during refolding kinetics
86, 73	CORE-N	Early (Fast phase) (17)
41	NMP	Early (Fast phase) (17)
193	CORE-C	Late (Slow phase) (17)
**Residue pairs used for FRET**	**Distance reported on**	**Formation of native like distance distribution during refolding kinetics**
58-86	CORE-N-NMP	Late (Slow phase) (18)
28-71	intra CORE-N	Early (Burst phase) (18, 19)
28-86	intra CORE-N	Early (Burst phase) (18)
73-203	CORE-N-CORE-C	Late (Slow phase) (20)
28-203	CORE-N-CORE-C	Late (Slow phase) (21)
18-203	CORE-N-CORE-C	Late (Slow phase) (18, 19)
188-203	intra CORE-C	Late (Slow phase) (21)
169-188	intra CORE-C	Late (Slow phase) (21, 22)

Table S2: Summary of the experimental results obtained from refolding kinetics of AKE compared with folding simulations of WT AKE (See Fig. 4). Citations in brackets in the last column are from the supporting references list (Text S5).
