# Supplementary material for: In the Multi-domain Protein Adenylate Kinase, Domain Insertion Facilitates Cooperative Folding while Accommodating Function at Domain Interfaces
Source: PLoS Comput Biol. 2014 Nov 13;10(11):e1003938. doi: 10.1371/journal.pcbi.1003938 (PMC4230728; doi:10.1371/journal.pcbi.1003938)
Supplement: Text S1 — Supporting methods. (PDF) [file pcbi.1003938.s009.pdf]

SUPPORTING METHODS (Citations are from the supporting references list: Text S5)

Structure-based models (SBMs) for the folding of WT AKE and its mutants. SBMs use potential energy functions defined by the native or folded state of a protein (as given in the X-ray or NMR structure) to calculate protein dynamics. Here, we use a C-α structure based model (SBM) constructed using the SMOG webserver (1) to perform molecular dynamics (MD) simulations. In this type of SBM, the protein is represented only by its C-α atoms. The potential energy (E) is composed of interactions between these C-α atoms and is as follows (2):

$$E = \sum_{bonds} K_r (r - r_0)^2 + \sum_{angles} K_\theta (\theta - \theta_0)^2 + \sum_{dihedrals}^{n=1,3} K_\varphi^{(n)} (1 - \cos(n(\varphi - \varphi_0))) + \sum_{contacts} \varepsilon_1 \left[5\left(\frac{\sigma_{ij}}{r_{ij}}\right)^{12} - 6\left(\frac{\sigma_{ij}}{r_{ij}}\right)^{10} \right] + \sum_{non-contacts} \varepsilon_2 \left(\frac{\sigma}{r_{ij}}\right)^{12}$$

Eq. S1

The first and second terms of E are harmonic potentials used to represent bond vibration and angle bending motions. The third term describes the dihedral angle rotation. The fourth term is a Lennard-Jones potential and represents the interactions between residues that form native contact pairs. The fifth term gives the repulsive (excluded volume) interaction between non-contact pairs i.e. those pairs which have neither covalent nor non-covalent interactions in the native state. The summations in the first to fifth terms are over all bonds, angles, dihedrals, native contacts and non-contacts respectively. The constants, r_0, θ_0, and φ_0 are calculated from the coordinates of C-α atoms in the native state of the protein. σ_{ij} is the distance between those C-α atoms, *i* and *j*, which are in contact in the native state. The set of native contacts for 4AKE.pdb (chain A, has 531 contacts), was calculated using the Contacts of Structural Units (CSU) software (3) and has been published previously (4). A contact between residues *i* and *j* (Fig. 1C) implies that at least one atom from residue *i* and one atom from residue *j* are in native contact according to CSU analysis. Contact lists are given in SI Text S3. The excluded volume term is calculated between C-α atoms that are not in native contact using an interaction distance of σ=0.4 nm. In

our simulations, the energy scale ε (= $\varepsilon_1 = \varepsilon_2$) is set equal to 1 kJ/mol. The values of the other force constants are K_r =100ε, K_θ =20ε, $K_\varphi^{(1)}$ =ε and $K_\varphi^{(3)}$ =0.5ε.

Generating the structure of the polyglycine loops which link WT termini in the circular permutants. The automodel class of MODELLER 9 (5, 6) was used to generate several polyglycine loop conformations which fit between the WT termini (residues 1 and 214). No WT atoms were perturbed during this procedure. The shortest length of loop that fits the gap between the WT termini and yet has a sufficient diversity of conformational structures, is chosen (here 4 glycines). Out of the different conformations of 4 glycine loops which can link the WT termini, one was chosen at random for our simulations. The loop was only used to constrain the polypeptide chain (by having bond, angle, dihedral angle and excluded volume interactions) and did not have any stabilizing native contact interactions either within the loop or between the loop and the rest of the protein.

Kinetic folding and unfolding simulations of WT AKE. MD simulations of the C-α SBM of WT AKE were performed using GROMACS 4.0 (7) at different temperatures above and below the folding temperature (T_f) in a canonical ensemble (NVT). A stochastic dynamics integrator was used to simulate Langevin dynamics. We used a time-step of 0.0005 ps and sampled the system every 2000 steps. Each trajectory was run for 2×10^8 time-steps and showed one or two folding and/or unfolding transitions. From these simulations, the WT AKE T_f was estimated to be ~1.1 (in units of k_BT). To ensure sufficient and unbiased sampling of the folding mechanism, further simulations were run at 6 different temperatures close to T_f (i.e. within $T_f \pm 0.025$ in k_BT units). 36 different folded structures and 36 unfolded structures were generated and simulations were performed at each of the 6 temperatures using each of these structures. Thus, a total of 432 kinetic simulations were performed and at least 100 transitions were obtained.

Identification of WT native contact clusters which fold and unfold together. The transition path (TP) (8) was defined as any piece of the simulation trajectory which started at the unfolded ensemble: Q=0.2 (or the folded ensemble: Q=0.8) and ended at the folded ensemble: Q=0.8 (or the unfolded ensemble: Q=0.2) without recrossing into the unfolded ensemble (or the folded ensemble). Each TP consists of a series of simulation snapshots. Each of these snapshots was represented as a vector of 531 binary elements with each element corresponding to a native contact of WT AKE. An element equals 1 if that native contact is formed and 0 otherwise. All TPs ($\sim 2.2\times10^5$ snapshots) were pooled into a single ($\sim 2.2\times10^5 \times 531$) matrix M. The Matlab function corrcoef() was then used to obtain the 531 × 531 cross-correlation matrix, Z (=corrcoef(M)). If two contacts *i* and *j* form or break together, their crosscorrelation coefficient $z_{ij}\sim1$. Similarly uncorrelated contacts have $z_{ij}\sim0$, and anticorrelated contacts have $z_{ij}\sim-1$. The values of the z_{ij}'s were found to lie between −0.1 and 1. Thus, there is very little anticorrelation between the contacts and the z_{ij}'s can be rescaled and normalized to lie between 0 and 1 without introducing large errors. This gave the normalized cross-correlation matrix, Z_N.

In order to analyze parts of AKE which form and unfold together, we clustered the elements of Z_N using the CLANS software (9). CLANS is usually used to cluster protein sequences. We repurposed CLANS to construct a graph, whose vertices represent the native contacts. An edge between vertices (contacts) *i* and *j* provides an attractive force proportional to the normalized z_{ij} (i.e. elements of Z_N). The contact clustering takes place by computing the pair-wise forces and evolving all the vertices of the graph in an iterative manner till the distance between the vertices reached equilibrium. Subsequently, the weakest edges (normalized z_{ij} less than a cutoff value, C) were removed, and the graph was re-equilibrated. This procedure was repeated several times while progressively increasing C until equilibration was achieved at the desired C. After equilibration, the number and the nature of the clusters were examined. At low values of C, few edges get deleted and a single cluster was seen. Two clusters (corresponding to the

contacts of LID and CORE) were seen for C~0.223; three (LID, CORE-N and CORE-C) for 0.357<C<0.431; two (CORE-N and CORE-C) for 0.511<C<0.693 and none (the network becomes diffuse as almost all the edges are removed) above C=0.8. We chose C=0.431 in order to best compare with the three foldons seen in HX-NMR experiments (10). These three clusters (LID, CORE-N and CORE-C) and the native contacts that comprise them are shown in Fig. S1.

Domain and contact cluster definitions. The three domains in AKE are defined as: NMP: residues 30–67, LID: residues 118–160 and CORE: residues 1–29, 68–117 and 161–214. The CORE domain is further split into two regions that are identified by contact clustering (Fig. S1B): CORE-N: residues 1–29 and 68–117, and CORE-C: residues 161–214.

In most cases, the contacts specific to a given region are defined as all those contacts which are between the residues within that region and these were used to calculate the RCs (Figs. 3, 5, 6, S2, S7). However, the CORE-N contacts also contain all contacts from the CORE-NMP interface and the CORE-C contacts also contain contacts between CORE-C and CORE-N. The CORE-LID interface contacts are not part of any of the regions. Numbers of native contacts (N in Eq. S2) used in the simulation analyses of all proteins in this manuscript are: Q: 531; Q_{NMP}: 59; Q_{LID}: 89; Q_{CORE}: 328, $Q_{CORE\text{-}N}$: 197; $Q_{CORE\text{-}C}$: 164. The following are different for ΔCORE-NMPi: Q^*_{NMP}: 75.5, Q^*_{CORE}: 344.5, $Q^*_{CORE\text{-}N}$: 180.5, and for ΔCORE-LIDi: Q*: 530.5, Q^*_{LID}: 99.5, Q^*_{CORE}: 339, $Q^*_{CORE\text{-}N}$: 199.73, $Q^*_{CORE\text{-}C}$: 172.27. The number of contacts at the domain interfaces are: CORE-NMP: 33; and CORE-LID: 22.

We next list the two differences between these contact definitions (Fig. 2C) and those obtained from the CLANS clusters (Fig. S1B). 5 out of the 22 CORE-LID interface contacts were part of the CLANS CORE-C cluster (Fig. S1B). Only 14 out of the 33 CORE-NMP interface contacts were part of the CLANS CORE-N cluster (Fig. S1B).

Equilibrium folding simulations of WT AKE and its mutants. For each model of AKE, multiple, independent simulations with different initial conformations of the folded and the unfolded states were performed for ~2×10^9 timesteps over a wide range of temperatures (k_BT ~ 1 to 1.123). The folding temperature (T_f) of each protein was estimated from these simulations as that temperature at which both folding and unfolding transitions are seen. Multiple, independent simulations at the estimated T_f were then performed for each of the models such that at least 15 transitions were observed. A transition is defined as going from a folded state (N) to an unfolded state (U) back to N or going from U to N back to U. To account for equilibration of the system, the initial 1×10^5 timesteps of each independent simulation were ignored. All the simulation trajectories of a given protein were then concatenated and analyzed to obtain folding free energy profiles. All other simulation parameters and the sampling rate were the same as used in the kinetic simulations of the WT.

We use the fraction of native contacts (Q) as the reaction coordinate for the folding free energy profiles (FEPs). Q, for any snapshot of the simulation, is defined according to the following equations:

$$Q = \frac{\sum_{contacts} Q_{ij}}{0.6N} \qquad \text{Eq. S2}$$

$$Q_{ij} = \theta\left(c_{ij} - r_{ij}\right)\omega_{ij}\left[\left(1-\left(\frac{r_{ij}}{\sigma_{ij}}\right)^{n}\right)\Big/\left(1-\left(\frac{r_{ij}}{\sigma_{ij}}\right)^{m}\right)\right] \qquad \text{Eq. S3}$$

In Eq. S2, N is the total number of native contacts. In Eq. S3, Q_{ij} quantifies the formation of a native contact defined between a pair of residues *i* and *j* (C-α atoms in our model). $\theta(x)$ is a step function which equals 1 when $x>0$ and equals 0 when $x\leq0$. c_{ij} is the cut-off distance beyond which no contact is formed. Here, c_{ij} is set to $1.5\sigma_{ij}$, where σ_{ij} is the native contact distance between C-α atoms *i* and *j*. r_{ij} is the

distance between the same atoms in a snapshot of the simulation. ω_{ij} is a weighting factor which equals the strength of the contact between *i* and *j* and usually equals 1. For the contacts of ΔCORE-NMPi and ΔCORE-LIDi which are rescaled, ω_{ij} is equal to the scaling factor. *n* and *m* are equal to 6 and 10 respectively. The value of Q_{ij} at $r_{ij}=\sigma_{ij}$ is not defined, but the left and the right limits exist and are equal to 0.6. When C-α atoms *i* and *j* are almost in contact, $Q_{ij}\approx 0.6$. The sum over contacts in Eq. S2 is normalized by $0.6N$ in order to account for this factor. So, for all practical purposes, Q lies between 0, (when no contacts are formed) and 1 (when all contacts are formed). The calculation of values of reaction coordinates which include only a subset of contacts (e.g. Q_{CORE}, Q_{LID}, Q_{NMP}) are performed using the same equations (Eq. S2 and S3) except the summation in Eq. S2 is now over the subset of contacts and the normalization is also by the number of contacts in that subset.

The logarithm of the density of states as a function of Q (and other reaction coordinates) calculated from the concatenated simulations is then used to calculate the FEPs and the 2D free energy surfaces. The populations are reweighted using the Boltzmann factor to a temperature where the folded and the unfolded states not equally populated. This temperature is the calculated T_f. The simulation temperatures (i.e. estimated T_fs) and the calculated T_fs are shown in Table S1.

Free energy barriers in folding simulations can be best calculated at close to T_f because at this temperature the protein transitions several times between the folded and the unfolded states. The folding barriers between different proteins can also be best compared at T_f because the folded and the unfolded ensembles of the proteins are equally populated. Thus, the effect of protein stability (difference between the free energy of the folded and the unfolded ensembles at zero denaturant concentration) on the folding rate is removed and the effect of the free energy barrier can be isolated. But the T_fs of different proteins are different and to account for these differences during comparison of FEPs, the free energies are normalized by k_BT_f.

Estimating the variance of the folding free energy, T_f and folding cooperativity. We use the jackknife method (11, 12) to estimate the variance of and in turn the errors in the folding free energy along Q for WT AKE and its mutants. As mentioned earlier, we performed several independent simulations at the estimated folding temperature T_f for each protein and we call this number, *n* (=16). In order to estimate the variance in the total FEP, *F(Q,n)*, we compute the *n* partial FEPs, $F_i(Q,n-1)$, from the concatenated trajectory of only *n–1* simulations obtained by omitting the ith simulation trajectory. Errors in FEPs can occur because of under-sampling of barrier crossing events. They can also occur because the finite simulation time makes the sampling of one of the folded or the unfolded ensembles higher (even if the simulation is run at the true T_f) and this creates errors in the position of the folded and unfolded basins. In order to separate these two types of errors, we reweight the $F_i(Q,n-1)$'s such that their folded and unfolded basins are equally populated. The free energy profile is then Boltzmann reweighted to a temperature where the folded and unfolded states are equally populated. The variance, *Var(Q)*, calculated from these FEPs using the following equation gives an estimate on errors created by under-sampling the barrier region.

$$Var(Q) = \frac{n-1}{n}\sum_{i=1}^{n}\left(F_i(Q,n-1) - \langle F_i(Q,n-1)\rangle\right)^2 \qquad \text{Eq. S4}$$

The quantity in the angle brackets is the average of the *n* free energy profiles $F_i(Q,n-1)$. Twice the square root of *Var(Q)* given the sizes of the error bar shown on the FEPs in Figs. 3, 5, 6, S2 and S7. The free energies of two different proteins at the same *Q* can be assumed to be different if their error bars do not overlap and by this criteria the foldedness of ΔCORE-NMPcut and ΔCORE-LIDcut are different from those of WT AKE. However, it is known that using slightly different C-α SBM contact maps can create minor changes in the top of the folding free energy barrier (both shape and height) which may not

be relevant to the phenomenon being investigated. In order to avoid such over-interpretation, we assume that free energy barriers are the same unless they differ by ~2 $k_B T$.

In order to estimate the errors caused by the unbalanced sampling of the folded and the unfolded basins we calculate the variance of T_f by using the $T_{f,i}(n-1)$ calculated from reweighting the $F_i(Q,n-1)$'s such that each profile has equal populations of folded and unfolded states. Using the jackknife method, the variance in T_f is given by:

$$VarT_f = \frac{n-1}{n}\sum_{i=1}^{n}(T_{f,i}(n-1) - \langle T_{f,i}(n-1)\rangle)^2 \qquad \text{Eq. S5}$$

where, the quantity in angle brackets is the average of the n folding temperatures $T_{f,i}(n-1)$. The square root of $VarT_f$ is given as the error estimate in Table S1 in units of $k_B T_f$.

We also compute the variance in the folding cooperativity by using a similar procedure. Folding cooperativity is estimated by the ratio of the change in the van't Hoff enthalpy to the change in the calorimetric enthalpy (13) and is given by the following equation:

$$\kappa(n) = \frac{\Delta H_{vH}(n)}{\Delta H_{cal}(n)} = \frac{2\sqrt{k_B T_f^2(n) C_v(T,n)_{\max}}}{\int_{T_f - T_{\min}}^{T_f + T_{\max}} C_v(T,n)\,dT} \qquad \text{Eq. S6}$$

where, $\kappa(n)$ is the folding cooperativity, $\Delta H_{vH}(n)$ is the change is the van't Hoff enthalpy, $\Delta H_{cal}(n)$ is the change in the calorimetric enthalpy, $T_f(n)$ is the folding temperature, $C_v(T,n)$ is the heat capacity curve, and $C_v(T,n)_{max}$ is the maximum value of $C_v(T,n)$ which is the C_v at the transition temperature $T_f(n)$. The label n means that the values are calculated from the entire folding trajectory (a concatenation of n independent simulations). $C_v(T,n)$ was computed from the Boltzmann reweighted histogram of the total

energy of the system at $T_f(n)$ (13). We used a value of 10 K for T_{min} and T_{max}. The values of $\kappa(n)$ for WT AKE and its mutants are shown in Fig. S3. The variance in $\kappa(n)$ for each model of AKE was computed using the equivalent form of Eq. S4 for $\kappa(n)$. The error bars shown in Fig. S3 correspond to twice the square root of the variance in $\kappa(n)$.

Conformational transition simulations for WT AKE and its mutants. The model used to obtain the open to closed state conformational transitions in WT AKE was generated as described previously (4). In brief, 39 contacts specific to the closed state of AKE (1AKE.pdb, chain A), but absent in the open state (4AKE.pdb, chain A) were appended to the open state contacts and used to drive the conformational transitions. The native contact distance for these contacts is calculated from the closed state. Control folding simulations of a model of WT AKE which includes the 39 closed state specific contacts were similar to those of WT (with only open state contacts). The same 39 contacts (renumbered as necessary) were used to drive the conformational transitions in the mutants of AKE. In order to achieve an equal population of open and closed states, the energies of the closed state specific contacts (Fig. 1C; red contacts) were multiplied by a scaling factor ε_3 (Table S1). The simulations were performed below T_f where AKE does not unfold but only transitions between the open and the closed states. Simulations were performed in the canonical ensemble using GROMACS 4.0 using a stochastic dynamics integrator with a timestep of 0.0005 ps. There were at least 20 open to closed to open transitions for each mutant.

Analysis of the Pfam database for identifying multi-domain proteins having domain insertions. The Pfam database (14) classifies protein families based on sequence similarity. Proteins within a Pfam family have similar structural and functional characteristics, and are expected to be evolutionarily related (14). Based on these criteria, each Pfam family constitutes a unique protein domain. Pfam also

provides information on the number, nature and arrangement of domains in proteins based on sequence similarity to manually curated sequence signatures of known Pfam families. For several proteins, structural evidence for the domain architectures defined in Pfam can be found in the Protein Data Bank. Our objective here is to calculate structural features, such as the absolute contact order (ACO), chain length and packing fraction of the insert and the discontinuous domains in proteins having inserted domains.

We used the high quality, manually curated Pfam-A database to identify proteins having structural evidence of inserted domains. The Pfam-A database includes a list of Pfam families which are known to be nested i.e. these families are predicted to have overlapping domain boundaries determined by using sequence homology. We found 88 nested families listed in Pfam-A. For each of these families we obtained structures from the PDB in which at least one domain belongs to that family. We obtained 3726 such structures (PDB IDs) in total. These structures were first processed to remove multiple models (primarily NMR structures) while retaining only the first model. In cases where alternative side chain conformations were present only one conformation was retained. In addition, structures having non-natural amino acids were excluded, except when the non-natural amino acid was selenomethionine. Multiple chains in a PDB ID are listed separately in Pfam with their respective domain annotations and we chose to perform our analysis on each PDB ID-chain ID combination separately. We obtained 6147 PDB ID-chainIDs and this comprises our dataset for calculating the structural features of interest.

For each PDB ID-chainID, we obtained the number of domains and their boundaries from Pfam. We ignored domains from Pfam whose chain lengths were less than 10 amino acids. When multiple domains are present in a PDB ID-chain ID we compared the domain boundaries of the constituent domains to identify their connectivity i.e. whether they are singly linked or whether they are inserted. We consider a domain to be inserted if there are at least 20 amino acids of the same discontinuous domain beyond both

its N- and C-termini. On the other hand, a domain is singly linked if it is not inserted in any other domain, and if it is directly succeeded by another domain. We applied these criteria to our dataset and obtained 1713 instances of domain insertion (i.e. insert–discontinuous domain pairs) belonging to 36 Pfam families, 2764 instances of singly linked domains belonging to 55 Pfam families and 2428 instances of single domains belonging to 56 Pfam families from our analysis. For each of these instances, we calculated the packing fraction of native contacts, the chain length and the absolute contact order (ACO) for the inserted domains, the discontinuous domain into which these were inserted, as well as the singly-linked and the single domains. C-α native contacts were pre-calculated for all the 6147 PDB ID-chain IDs using the CSU software (3) and were used for calculating the packing fraction (ratio of number of contacts to the chain length) and absolute contact order (Eq. S7). While calculating contact order for discontinuous domains, the polypeptide segments were renumbered so that they become continuous. This allowed us to obtain the contact order of the discontinuous domains independent of the length of the inserted domain.

$$ACO = \frac{\sum_{\forall i,j \in contacts} |i - j|}{N} \qquad \text{Eq. S7}$$

The discontinuous domains of 1079 out of the 1713 protein chains have all the structural parameters higher than those of the inserted domains. This is also true for every protein chain from 21 of the 36 inserted domain Pfam families. We next list the potential biases in our analysis.

We have not manually curated the structural data and it is subject to domain annotation errors. We clearly see these in some families (e.g. PF06470). Further, several inserted domains are annotated only textually as inserts (but not in the sequence analysis: e.g. PF00702) and thus there are errors of omission. For ease of analysis, we have used every protein chain with an inserted domain. So, some proteins are

represented multiple times. Finally, the data is dependent on structural characterization and is inherently biased towards more stable proteins. There are also likely to be biases present in quantities such as the packing fraction due to the quality and the type (either NMR or X-ray) of the structures.

Domains which are both inserted and singly linked. The question that arises from the above analyses is whether domains lose stability when inserted into another domain either to increase folding cooperativity or to gain function. The inserted domains of AKE do not appear in contexts outside the CORE domain in the Pfam database. However, other inserted domains from the above dataset do occur as independent or singly-linked domains. We found that 19 of the 36 families which having structural evidence for domain insertion also have structural evidence for existing as singly-linked or independent domains. We find that the ACO, the chain length and the packing fraction of domains do not appear to differ significantly when they are inserted versus when they are singly-linked or when they exist independently. We also plotted sequence specific quantities such as the number of hydrophobic residues and found that these too do not differ by much between inserted domains and structurally similar single/singly-linked domains.
