# Supplementary material for: In the Multi-domain Protein Adenylate Kinase, Domain Insertion Facilitates Cooperative Folding while Accommodating Function at Domain Interfaces
Source: PLoS Comput Biol. 2014 Nov 13;10(11):e1003938. doi: 10.1371/journal.pcbi.1003938 (PMC4230728; doi:10.1371/journal.pcbi.1003938)
Supplement: Text S2 — Supporting results. (PDF) [file pcbi.1003938.s010.pdf]

SUPPORTING RESULTS (Citations are from the supporting references list: Text S5)

Construction and the folding simulations of a pseudo-circular permutant (pCP) of AKE. In the main article we show that domain insertion is necessary for the three domains of AKE to have the same T_f. This makes WT AKE fold and unfold at the same T_f and increases folding cooperativity. In order to understand if domain insertion is sufficient to maintain WT-like domain-specific T_fs of NMP and LID, we generate a permutant of AKE whose central β-sheet is reordered (Fig. S7C-D). This destabilizes the CORE domain while keeping both LID and NMP inserted. We then study both the folding and the conformational transitions of this pCP. Whether a construct such as pCP will fold can only be determined by experiment however it allows us to perform control simulations.

To generate the pdb file for pCP, the central β-sheet of the CORE domain was re-connected so that the β-strand order was 54321 instead of 54132 (WT) (Fig. S7C). WT AKE was cut between residues 24 and 25, as well as, between residues 103 and 104. The ATOM entries of 4AKE.pdb, chain A were rearranged in the following order: 25 to 103, followed by 1 to 24 and finally 104 to 214 (WT numbering). The two discontinuities in the structure were fixed by linking residue 103 with residue 1 (WT numbering) by a 3 glycine loop and residue 24 to 104 (WT numbering) by a 6 glycine loop. After loop addition, pCP has 223 residues. The new N-terminus is at residue 25 (WT numbering). The C-terminus remains the same as that in WT. The contact map of pCP was generated by permuting and renumbering the contact map of the WT. No extra contacts were added for the loops (Figs. S7B and S7D).

The automodel class of MODELLER 9 (5, 6) was used to generate several polyglycine loop conformations which fit between the WT residues to be connected (103 to 1 and 24 to 104 in pCP). In order to test the effect of loop conformations, we compared the folding simulations of pCP with and

without loop dihedral interactions (i.e. in Eq. S1 $K_\varphi^{(1)}$, $K_\varphi^{(3)}$ =0 for dihedrals of both loops). We specifically explored the effect of polyglycine loop conformations on pCP for the following reason: the two polyglycine loops added to pCP are roughly in the centre of the central β-sheet of CORE. Our simulations with default loop dihedral interactions showed that pCP folded non-cooperatively through the formation of an intermediate in which CORE-N and CORE-C fold independent of each other (Fig. S7E and S7G). In order to ensure that this folding mechanism was not an artifact of adding the polyglycine loops in CORE, we simulated pCP without loop dihedral interactions. Having no dihedral interactions allows the loops to adopt a larger diversity of conformations. However, we found little difference between the dynamics of the two protein models (with and without dihedral interactions) either during folding or during conformational transition simulations. Thus, the observed folding mechanism of pCP was due to the reordering of the central β-sheet CORE and not due to the specific conformations of either of the loops. Figs. S7E-I shows data from simulations of a pCP with no polyglycine loop dihedral interactions.

In agreement with experiments on chimeric AKE variants which show that CORE determines the global T_f of AKE (16), the T_f (~1.04 in reduced units) of pCP is lower than those of WT and the two CPs (~1.1, see Table S1). Despite the changes in the folding of CORE (compare Fig. 3G with Fig. S7G), the folding of the inserted NMP and LID domains remains WT-like. Unlike in CP-NMPcut and CP-LIDcut (Fig. 6C, F), both NMP and LID are folded in the folded ensemble, N, (Fig. S7H, S7I). NMP has low intrinsic stability and unless its termini are brought together by the folding of CORE, it cannot fold. As in WT, inserted NMP folds and unfolds along with CORE in pCP (Fig. S7E, single native basin at Q~0.8, and Fig. S7I). The T_f of the inserted LID remains closer to that of WT AKE and higher than that of pCP and thus, it stays folded in the unfolded pCP ensemble (Fig. S7H, Q~0.35). Thus, domain insertion of NMP and LID is sufficient to maintain their WT-like domain-specific T_fs.

As in the CPs, the conformational transitions of pCP are observed to be similar to those of WT (i.e. like Fig. 7A). Thus, AKE could have evolved with alternate chain topologies and retained the same mechanism of conformational transitions (through a LID-closed-NMP-open intermediate).

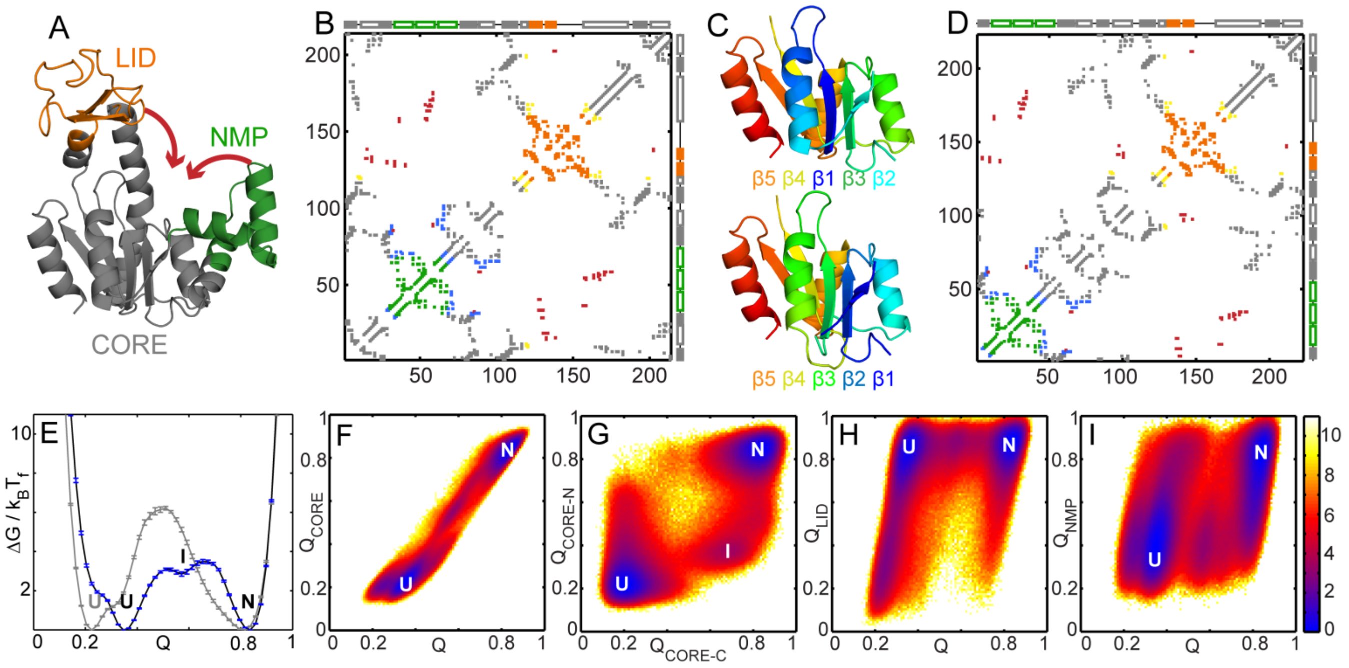

Figure S7 The model for pCP, its free energy profile and 2DFESs at T_f: (A) WT AKE with its three domains (B) The C-α contact map of WT AKE. X and Y axes represent residue number. The contacts are colored according to their location (CORE: grey, NMP: green, LID: orange, CORE-NMP interface: blue and CORE-LID interface: yellow). The red contacts are closed state specific contacts that drive conformational transitions. Secondary structure is shown along the axes: α-helices are empty boxes and β-strands are filled boxes. (C) The β-sheet from CORE and its flanking α-helices are shown for WT AKE (top) and pCP (below). These are colored from N- to C-terminus in blue to red. The β-strands of WT AKE are re-connected to get pCP. (D) The contact map of pCP with the same color scheme as in B. It can be seen both here and in C (yellow through red β-strands) that the C-terminal part of the protein is unperturbed. (E) The FEP along Q (black with blue error bars) shows that folding is less cooperative than in WT (grey). The error bars represent twice the square root of the variance. (F-I) The 2DFESs with RCs of (F) Q_{CORE} and Q, (G) Q_{CORE-N} and Q_{CORE-C}, (H) Q_{LID} and Q, (I) Q_{NMP} and Q. (G) shows that folding can proceed either through CORE-N or through CORE-C. All free energies are scaled by k_BT_f. N and U denote the native and the unfolded ensembles.
