# Supplementary material for: In the Multi-domain Protein Adenylate Kinase, Domain Insertion Facilitates Cooperative Folding while Accommodating Function at Domain Interfaces
Source: PLoS Comput Biol. 2014 Nov 13;10(11):e1003938. doi: 10.1371/journal.pcbi.1003938 (PMC4230728; doi:10.1371/journal.pcbi.1003938)
Supplement: Text S3 — Additional supporting information. Contact lists for WT AKE, some of which are used to define the reaction coordinates (RCs). (DOCX) [file pcbi.1003938.s011.docx]

**ADDITIONAL SUPPORTING INFORMATION**

**Contact lists for WT AKE, some of which are used to define the reaction coordinates (RCs).**

The 531 native contacts (Q): (1,24), (1,26), (1,79), (1,80), (1,81), (1,104), (1,105), (1,213), (2,72), (2,77), (2,80), (2,81), (2,100), (2,101), (2,102), (2,103), (2,104), (2,105), (3,20), (3,26), (3,81), (3,82), (3,83), (3,103), (3,105), (3,107), (3,213), (4,83), (4,86), (4,87), (4,96), (4,103), (4,105), (4,106), (4,107), (4,182), (5,13), (5,17), (5,20), (5,82), (5,83), (5,84), (5,85), (5,107), (5,109), (6,13), (6,87), (6,106), (6,107), (6,108), (6,109), (6,171), (6,175), (6,178), (6,179), (6,182), (6,193), (7,11), (7,13), (7,108), (7,109), (7,171), (8,13), (8,109), (8,111), (8,113), (8,116), (8,168), (8,171), (9,13), (9,116), (9,120), (9,164), (9,167), (9,168), (9,171), (10,116), (10,119), (10,120), (11,109), (11,111), (11,116), (11,119), (12,109), (13,17), (13,84), (13,85), (13,109), (14,18), (14,84), (15,19), (15,202), (16,20), (16,109), (16,202), (16,205), (16,206), (16,209), (17,21), (17,28), (17,82), (18,22), (19,23), (19,24), (19,206), (19,209), (19,210), (19,213), (20,24), (20,26), (20,82), (20,107), (20,209), (20,213), (21,25), (21,26), (21,27), (21,28), (21,82), (23,206), (23,210), (23,214), (24,210), (24,213), (24,214), (26,79), (26,80), (26,81), (26,82), (27,71), (27,76), (27,77), (27,80), (27,81), (27,82), (28,33), (28,71), (28,82), (28,84), (29,34), (29,68), (29,71), (29,72), (29,77), (29,81), (29,82), (29,83), (29,84), (30,34), (30,36), (30,84), (31,35), (31,64), (31,68), (31,84), (31,85), (31,86), (31,88), (32,36), (33,37), (34,38), (34,67), (34,68), (34,71), (35,39), (35,49), (35,53), (35,59), (35,64), (35,67), (36,40), (36,53), (37,41), (38,43), (38,46), (38,67), (39,46), (39,47), (39,50), (39,53), (42,47), (43,47), (44,48), (45,63), (45,66), (45,67), (45,70), (48,52), (48,63), (49,53), (49,59), (49,63), (50,54), (51,55), (52,56), (52,57), (52,58), (52,59), (52,60), (52,63), (53,57), (53,59), (59,63), (59,64), (60,64), (61,65), (61,89), (61,91), (61,92), (62,66), (63,67), (64,68), (64,86), (64,92), (65,69), (65,86), (65,92), (65,95), (66,70), (67,71), (68,72), (68,83), (68,86), (68,96), (68,101), (69,73), (69,99), (69,101), (70,74), (71,76), (71,77), (72,77), (72,81), (72,83), (72,101), (74,78), (76,80), (77,81), (81,101), (81,103), (83,96), (86,92), (86,96), (87,92), (87,93), (87,96), (87,178), (87,182), (88,92), (88,174), (88,175), (88,178), (89,93), (89,174), (89,178), (90,94), (90,177), (90,178), (90,181), (91,95), (92,96), (93,97), (93,178), (93,181), (93,182), (94,98), (94,181), (95,99), (96,101), (96,103), (96,182), (97,101), (97,181), (97,185), (97,190), (102,190), (103,182), (103,190), (103,191), (104,191), (104,192), (105,192), (105,194), (105,212), (105,213), (106,182), (106,191), (106,192), (106,193), (106,194), (107,194), (107,196), (107,209), (107,212), (107,213), (108,171), (108,179), (108,193), (108,194), (108,195), (108,196), (109,196), (109,198), (109,205), (109,209), (110,195), (110,196), (110,197), (110,198), (110,199), (111,115), (111,116), (111,119), (111,198), (112,116), (112,199), (113,117), (113,168), (113,172), (114,118), (115,119), (115,199), (116,120), (116,164), (116,168), (117,121), (117,161), (117,164), (117,165), (117,168), (118,122), (118,134), (118,136), (118,137), (119,123), (119,134), (119,137), (119,198), (119,199), (120,159), (120,164), (120,167), (121,159), (121,161), (121,164), (122,134), (122,135), (123,132), (123,133), (123,134), (123,155), (123,156), (123,159), (123,167), (124,133), (124,135), (124,140), (124,153), (124,154), (124,155), (125,130), (125,131), (125,132), (125,153), (125,154), (125,155), (125,156), (126,130), (126,131), (126,146), (126,149), (126,151), (126,152), (126,153), (127,154), (128,151), (131,146), (131,148), (131,149), (132,138), (133,138), (133,139), (133,140), (133,146), (133,147), (133,153), (134,138), (134,139), (135,139), (135,140), (139,147), (140,144), (140,145), (140,147), (140,153), (141,147), (142,147), (144,152), (144,153), (145,150), (145,151), (145,152), (145,153), (146,150), (146,151), (146,153), (155,159), (156,160), (158,163), (158,164), (158,167), (159,164), (160,164), (161,165), (162,166), (163,167), (164,168), (165,169), (166,170), (167,171), (168,172), (169,173), (170,174), (171,175), (171,179), (172,176), (173,177), (174,178), (175,179), (176,180), (177,181), (178,182), (179,183), (179,193), (180,184), (181,185), (182,186), (182,191), (182,193), (183,187), (183,193), (184,188), (185,190), (185,191), (186,191), (186,192), (186,193), (194,212), (195,212), (196,205), (196,208), (196,209), (196,212), (197,205), (197,208), (198,205), (200,204), (200,205), (200,208), (201,205), (202,206), (203,207), (204,208), (205,209), (206,210), (207,211), (208,212), (209,213), (210,214), (1,214), (3,24), (5,14), (5,16), (5,209), (6,85), (6,86), (7,85), (8,12), (8,172), (15,109), (24,209), (25,76), (25,79), (26,76), (29,33), (30,83), (31,83), (34,64), (37,71), (38,49), (38,70), (39,49), (40,47), (43,70), (46,50), (47,51), (49,67), (51,57), (64,88), (65,96), (65,99), (65,101), (68,81), (81,102), (97,102), (108,172), (111,199), (112,197), (113,169), (114,165), (120,158), (121,136), (121,155), (122,136), (123,158), (124,143), (124,144), (125,133), (125,159), (127,152), (130,156), (132,156), (133,148), (135,143), (139,143), (142,146), (142,150), (155,160), (157,163), (179,195), (204,211).

The 59 native contacts of NMP (Q_NMP_): (30,34), (30,36), (31,35), (31,64), (32,36), (33,37), (34,38), (34,67), (35,39), (35,49), (35,53), (35,59), (35,64), (35,67), (36,40), (36,53), (37,41), (38,43), (38,46), (38,67), (39,46), (39,47), (39,50), (39,53), (42,47), (43,47), (44,48), (45,63), (45,66), (45,67), (48,52), (48,63), (49,53), (49,59), (49,63), (50,54), (51,55), (52,56), (52,57), (52,58), (52,59), (52,60), (52,63), (53,57), (53,59), (59,63), (59,64), (60,64), (61,65), (62,66), (63,67), (34,64), (38,49), (39,49), (40,47), (46,50), (47,51), (49,67), (51,57).

The 89 native contacts of LID (Q_LID_): (118,122), (118,134), (118,136), (118,137), (119,123), (119,134), (119,137), (120,159), (121,159), (122,134), (122,135), (123,132), (123,133), (123,134), (123,155), (123,156), (123,159), (124,133), (124,135), (124,140), (124,153), (124,154), (124,155), (125,130), (125,131), (125,132), (125,153), (125,154), (125,155), (125,156), (126,130), (126,131), (126,146), (126,149), (126,151), (126,152), (126,153), (127,154), (128,151), (131,146), (131,148), (131,149), (132,138), (133,138), (133,139), (133,140), (133,146), (133,147), (133,153), (134,138), (134,139), (135,139), (135,140), (139,147), (140,144), (140,145), (140,147), (140,153), (141,147), (142,147), (144,152), (144,153), (145,150), (145,151), (145,152), (145,153), (146,150), (146,151), (146,153), (155,159), (156,160), (120,158), (121,136), (121,155), (122,136), (123,158), (124,143), (124,144), (125,133), (125,159), (127,152), (130,156), (132,156), (133,148), (135,143), (139,143), (142,146), (142,150), (155,160).

The 328 native contacts of CORE (Q_CORE_): (1,24), (1,26), (3,20), (3,26), (5,13), (5,17), (5,20), (6,13), (7,11), (7,13), (8,13), (9,13), (13,17), (14,18), (15,19), (16,20), (17,21), (17,28), (18,22), (19,23), (19,24), (20,24), (20,26), (21,25), (21,26), (21,27), (21,28), (3,24), (5,14), (5,16), (8,12), (68,72), (68,83), (68,86), (68,96), (68,101), (69,73), (69,99), (69,101), (70,74), (71,76), (71,77), (72,77), (72,81), (72,83), (72,101), (74,78), (76,80), (77,81), (81,101), (81,103), (83,96), (86,92), (86,96), (87,92), (87,93), (87,96), (88,92), (89,93), (90,94), (91,95), (92,96), (93,97), (94,98), (95,99), (96,101), (96,103), (97,101), (111,115), (111,116), (112,116), (113,117), (68,81), (81,102), (97,102), (1,79), (1,80), (1,81), (1,104), (1,105), (2,72), (2,77), (2,80), (2,81), (2,100), (2,101), (2,102), (2,103), (2,104), (2,105), (3,81), (3,82), (3,83), (3,103), (3,105), (3,107), (4,83), (4,86), (4,87), (4,96), (4,103), (4,105), (4,106), (4,107), (5,82), (5,83), (5,84), (5,85), (5,107), (5,109), (6,87), (6,106), (6,107), (6,108), (6,109), (7,108), (7,109), (8,109), (8,111), (8,113), (8,116), (9,116), (10,116), (11,109), (11,111), (11,116), (12,109), (13,84), (13,85), (13,109), (14,84), (16,109), (17,82), (20,82), (20,107), (21,82), (26,79), (26,80), (26,81), (26,82), (27,71), (27,76), (27,77), (27,80), (27,81), (27,82), (28,71), (28,82), (28,84), (29,68), (29,71), (29,72), (29,77), (29,81), (29,82), (29,83), (29,84), (6,85), (6,86), (7,85), (15,109), (25,76), (25,79), (26,76), (161,165), (162,166), (163,167), (164,168), (165,169), (166,170), (167,171), (168,172), (169,173), (170,174), (171,175), (171,179), (172,176), (173,177), (174,178), (175,179), (176,180), (177,181), (178,182), (179,183), (179,193), (180,184), (181,185), (182,186), (182,191), (182,193), (183,187), (183,193), (184,188), (185,190), (185,191), (186,191), (186,192), (186,193), (194,212), (195,212), (196,205), (196,208), (196,209), (196,212), (197,205), (197,208), (198,205), (200,204), (200,205), (200,208), (201,205), (202,206), (203,207), (204,208), (205,209), (206,210), (207,211), (208,212), (209,213), (210,214), (179,195), (204,211), (1,213), (3,213), (4,182), (6,171), (6,175), (6,178), (6,179), (6,182), (6,193), (7,171), (8,168), (8,171), (9,164), (9,167), (9,168), (9,171), (15,202), (16,202), (16,205), (16,206), (16,209), (19,206), (19,209), (19,210), (19,213), (20,209), (20,213), (23,206), (23,210), (23,214), (24,210), (24,213), (24,214), (1,214), (5,209), (8,172), (24,209), (87,178), (87,182), (88,174), (88,175), (88,178), (89,174), (89,178), (90,177), (90,178), (90,181), (93,178), (93,181), (93,182), (94,181), (96,182), (97,181), (97,185), (97,190), (102,190), (103,182), (103,190), (103,191), (104,191), (104,192), (105,192), (105,194), (105,212), (105,213), (106,182), (106,191), (106,192), (106,193), (106,194), (107,194), (107,196), (107,209), (107,212), (107,213), (108,171), (108,179), (108,193), (108,194), (108,195), (108,196), (109,196), (109,198), (109,205), (109,209), (110,195), (110,196), (110,197), (110,198), (110,199), (111,198), (112,199), (113,168), (113,172), (115,199), (116,164), (116,168), (117,161), (117,164), (117,165), (117,168), (108,172), (111,199), (112,197), (113,169), (114,165).

The 197 native contacts of CORE-N (Q_CORE-N_): (1,24), (1,26), (3,20), (3,26), (5,13), (5,17), (5,20), (6,13), (7,11), (7,13), (8,13), (9,13), (13,17), (14,18), (15,19), (16,20), (17,21), (17,28), (18,22), (19,23), (19,24), (20,24), (20,26), (21,25), (21,26), (21,27), (21,28), (3,24), (5,14), (5,16), (8,12), (68,72), (68,83), (68,86), (68,96), (68,101), (69,73), (69,99), (69,101), (70,74), (71,76), (71,77), (72,77), (72,81), (72,83), (72,101), (74,78), (76,80), (77,81), (81,101), (81,103), (83,96), (86,92), (86,96), (87,92), (87,93), (87,96), (88,92), (89,93), (90,94), (91,95), (92,96), (93,97), (94,98), (95,99), (96,101), (96,103), (97,101), (111,115), (111,116), (112,116), (113,117), (68,81), (81,102), (97,102), (1,79), (1,80), (1,81), (1,104), (1,105), (2,72), (2,77), (2,80), (2,81), (2,100), (2,101), (2,102), (2,103), (2,104), (2,105), (3,81), (3,82), (3,83), (3,103), (3,105), (3,107), (4,83), (4,86), (4,87), (4,96), (4,103), (4,105), (4,106), (4,107), (5,82), (5,83), (5,84), (5,85), (5,107), (5,109), (6,87), (6,106), (6,107), (6,108), (6,109), (7,108), (7,109), (8,109), (8,111), (8,113), (8,116), (9,116), (10,116), (11,109), (11,111), (11,116), (12,109), (13,84), (13,85), (13,109), (14,84), (16,109), (17,82), (20,82), (20,107), (21,82), (26,79), (26,80), (26,81), (26,82), (27,71), (27,76), (27,77), (27,80), (27,81), (27,82), (28,71), (28,82), (28,84), (29,68), (29,71), (29,72), (29,77), (29,81), (29,82), (29,83), (29,84), (6,85), (6,86), (7,85), (15,109), (25,76), (25,79), (26,76), (28,33), (29,34), (29,33), (30,84), (31,68), (31,84), (31,85), (31,86), (31,88), (34,68), (34,71), (45,70), (61,89), (61,91), (61,92), (64,68), (64,86), (64,92), (65,69), (65,86), (65,92), (65,95), (66,70), (67,71), (30,83), (31,83), (37,71), (38,70), (43,70), (64,88), (65,96), (65,99), (65,101).

The 164 native contacts of CORE-C (Q_CORE-C_): (161,165), (162,166), (163,167), (164,168), (165,169), (166,170), (167,171), (168,172), (169,173), (170,174), (171,175), (171,179), (172,176), (173,177), (174,178), (175,179), (176,180), (177,181), (178,182), (179,183), (179,193), (180,184), (181,185), (182,186), (182,191), (182,193), (183,187), (183,193), (184,188), (185,190), (185,191), (186,191), (186,192), (186,193), (194,212), (195,212), (196,205), (196,208), (196,209), (196,212), (197,205), (197,208), (198,205), (200,204), (200,205), (200,208), (201,205), (202,206), (203,207), (204,208), (205,209), (206,210), (207,211), (208,212), (209,213), (210,214), (179,195), (204,211), (1,213), (3,213), (4,182), (6,171), (6,175), (6,178), (6,179), (6,182), (6,193), (7,171), (8,168), (8,171), (9,164), (9,167), (9,168), (9,171), (15,202), (16,202), (16,205), (16,206), (16,209), (19,206), (19,209), (19,210), (19,213), (20,209), (20,213), (23,206), (23,210), (23,214), (24,210), (24,213), (24,214), (1,214), (5,209), (8,172), (24,209), (87,178), (87,182), (88,174), (88,175), (88,178), (89,174), (89,178), (90,177), (90,178), (90,181), (93,178), (93,181), (93,182), (94,181), (96,182), (97,181), (97,185), (97,190), (102,190), (103,182), (103,190), (103,191), (104,191), (104,192), (105,192), (105,194), (105,212), (105,213), (106,182), (106,191), (106,192), (106,193), (106,194), (107,194), (107,196), (107,209), (107,212), (107,213), (108,171), (108,179), (108,193), (108,194), (108,195), (108,196), (109,196), (109,198), (109,205), (109,209), (110,195), (110,196), (110,197), (110,198), (110,199), (111,198), (112,199), (113,168), (113,172), (115,199), (116,164), (116,168), (117,161), (117,164), (117,165), (117,168), (108,172), (111,199), (112,197), (113,169), (114,165).

The 33 native contacts of the CORE-NMP interface: (28,33), (29,34), (29,33), (30,84), (31,68), (31,84), (31,85), (31,86), (31,88), (34,68), (34,71), (45,70), (61,89), (61,91), (61,92), (64,68), (64,86), (64,92), (65,69), (65,86), (65,92), (65,95), (66,70), (67,71), (30,83), (31,83), (37,71), (38,70), (43,70), (64,88), (65,96), (65,99), (65,101).

The 39 closed state specific contacts that drive conformational transitions (4): (14,132), (15,132), (15,138), (18,130), (18,131), (18,132), (18,133), (28,129), (28,130), (32,85), (33,127), (33,129), (33,130), (36,156), (36,157), (36,158), (40,127), (40,128), (53,158), (53,167), (54,157), (54,158), (54,163), (55,163), (55,166), (56,163), (56,166), (56,167), (56,170), (57,166), (57,170), (58,88), (58,89), (58,170), (58,171), (58,174), (58,175), (137,202), (138,202).
