# Supplementary material for: In the Multi-domain Protein Adenylate Kinase, Domain Insertion Facilitates Cooperative Folding while Accommodating Function at Domain Interfaces
Source: PLoS Comput Biol. 2014 Nov 13;10(11):e1003938. doi: 10.1371/journal.pcbi.1003938 (PMC4230728; doi:10.1371/journal.pcbi.1003938)
Supplement: Text S4 — List of all PDBs having insert-discontinuous domain pairs used in the structural bioinformatics analysis. (DOCX) [file pcbi.1003938.s012.docx]

**List of all the PDBs having insert-discontinuous domain pairs used in the structural bioinformatics analysis.**

**Notes:**

1. Lines beginning with ### denote the Pfam ID of the inserted domain. The entries following it are PDB chains having an inserted domain which belongs to this Pfam ID. There are 36 Pfam IDs and 1713 PDB chains in total.

2. Format for each entry is as follows:

# PDBID ChainID

domain boundaries of inserted domain

domain boundaries of discontinuous domain

Example :

# 2FSG B

228 317

13 227 318 402

In the above example, PDB ID is 2FSG, chainID is B, inserted domain is residues 228 to 317, and the discontinuous domain is residues 13 to 227 and residues 318 to 402.

3. For 1QDM, the residue numbers were renumbered according to: 6P-27P to 6-27, 2-247 to 28-273, 1S-104S to 274-377, and 248-338 to 278-468, before analysis.

**PDB list:**

### PfamID PF01043 ###

# 2FSG B

228 317

13 227 318 402

# 2VDA A

228 358

9 227 359 402

# 2FSI B

228 318

11 227 319 402

# 2FSF B

228 313

12 227 314 402

# 2FSH B

228 313

13 227 314 402

# 2IPC D

228 352

6 227 353 396

# 2IPC C

228 352

5 227 353 396

# 2IPC B

228 352

6 227 353 396

# 2IPC A

228 352

5 227 353 396

# 3IQY A

226 338

5 225 339 382

# 2IBM A

226 338

5 225 339 382

# 2IBM B

226 338

11 225 339 382

# 3DL8 A

226 338

6 225 339 382

# 3DL8 B

226 338

6 225 339 382

# 3JV2 B

226 338

14 225 339 382

# 3JV2 A

226 338

15 225 339 382

# 3IQM A

226 338

5 225 339 382

# 1TF2 A

226 338

5 225 339 382

# 1TF5 A

226 338

5 225 339 382

# 1M74 A

226 338

5 225 339 382

# 1M6N A

226 338

5 225 339 382

# 3DIN B

271 383

1 270 384 427

# 3DIN A

271 383

1 270 384 427

# 3JUX A

271 383

4 270 384 427

# 1NL3 B

227 339

2 226 340 383

# 1NL3 A

227 339

2 226 340 383

# 1NKT A

227 339

2 226 340 383

# 1NKT B

227 339

2 226 340 383

### PfamID PF05189 ###

# 3PQV A

182 284

5 181 285 339

# 3PQV B

182 284

5 181 285 339

# 3PQV D

182 284

5 181 285 339

# 3PQV C

182 284

6 181 285 339

# 3TV1 B

184 278

8 183 279 329

# 3TV1 A

184 278

8 183 279 329

# 3KGD D

184 278

8 183 279 329

# 3KGD A

184 278

8 183 279 329

# 3KGD B

184 278

8 183 279 329

# 3KGD C

184 278

8 183 279 329

# 1QMI D

184 278

8 183 279 329

# 1QMI C

184 278

8 183 279 329

# 1QMI B

184 278

8 183 279 329

# 1QMI A

184 278

8 183 279 329

# 1QMH B

184 278

8 183 279 329

# 1QMH A

184 278

8 183 279 329

# 3TUX A

184 278

8 183 279 329

# 3TUT A

184 278

8 183 279 329

# 3TW3 A

184 278

8 183 279 329

### PfamID PF00037 ###

# 3MM6 E

234 256

125 233 257 344

# 3MM6 B

234 256

125 233 257 344

# 3MMC E

234 256

125 233 257 344

# 3MMC B

234 256

125 233 257 344

# 3MMA B

234 256

125 233 257 344

# 3MMA E

234 256

125 233 257 344

# 3MM7 B

234 256

125 233 257 344

# 3MM7 E

234 256

125 233 257 344

# 3MM9 B

234 256

125 233 257 344

# 3MM9 E

234 256

125 233 257 344

# 3MMB E

234 256

125 233 257 344

# 3MMB B

234 256

125 233 257 344

# 3MM8 E

234 256

125 233 257 344

# 3MM8 B

234 256

125 233 257 344

# 3MM5 E

234 256

125 233 257 344

# 3MM5 B

234 256

125 233 257 344

# 2VPW B

83 106

49 82 107 145

# 2VPW F

83 106

49 82 107 145

# 2VPY F

83 106

49 82 107 145

# 2VPY B

83 106

49 82 107 145

# 2VPX F

83 106

49 82 107 145

# 2VPX B

83 106

49 82 107 145

# 2VPZ F

83 106

49 82 107 145

# 2VPZ B

83 106

49 82 107 145

### PfamID PF12195 ###

# 3JU4 A

424 506

313 423 507 755

# 3GVL A

424 506

313 423 507 755

# 3GVK B

424 506

313 423 507 755

# 3GVK C

424 506

313 423 507 755

# 3GVK A

424 506

313 423 507 755

# 3GVJ A

424 506

313 423 507 755

# 1V0E B

424 506

313 423 507 755

# 1V0E F

424 506

313 423 507 755

# 1V0E C

424 506

313 423 507 755

# 1V0E E

424 506

313 423 507 755

# 1V0E D

424 506

313 423 507 755

# 1V0E A

424 506

313 423 507 755

# 1V0F E

424 506

313 423 507 755

# 1V0F B

424 506

313 423 507 755

# 1V0F A

424 506

313 423 507 755

# 1V0F C

424 506

313 423 507 755

# 1V0F F

424 506

313 423 507 755

# 1V0F D

424 506

313 423 507 755

### PfamID PF06415 ###

# 3IGZ B

95 327

15 94 328 547

# 3IGY B

95 327

15 94 328 547

# 3NVL A

94 326

14 93 327 546

# 3NVL B

94 326

14 93 327 546

# 1EQJ A

82 308

4 81 309 506

# 1EJJ A

82 308

4 81 309 506

# 1O99 A

82 308

4 81 309 506

# 1O98 A

82 308

4 81 309 506

# 2IFY A

81 307

3 80 308 505

### PfamID PF10143 ###

# 3KD8 A

38 205

2 37 206 394

# 3KD8 B

38 205

2 37 206 394

# 3IDD B

39 206

3 38 207 395

# 3IDD A

39 206

3 38 207 395

# 2ZKT A

41 167

5 40 168 403

# 2ZKT B

41 210

5 40 211 403

### PfamID PF01476 ###

# 2L9Y A

65 108

6 64 109 166

### PfamID PF02225 ###

# 3EIF A

363 449

125 362 450 582

# 1XF1 B

363 449

125 362 450 582

# 1XF1 A

363 449

125 362 450 582

# 3I6S B

345 456

139 344 457 607

# 3I6S A

345 456

139 344 457 607

# 3I74 B

345 456

139 344 457 607

# 3I74 A

345 456

139 344 457 607

### PfamID PF01658 ###

# 3CIN A

212 317

2 211 318 373

# 1U1I D

1429 1533

1201 1428 1534 1589

# 1U1I B

629 733

401 628 734 789

# 1U1I A

229 333

1 228 334 389

# 1U1I C

1029 1133

801 1028 1134 1189

# 3QW2 B

229 333

1 228 334 389

# 3QW2 D

229 333

1 228 334 389

# 3QW2 C

229 333

1 228 334 389

# 3QW2 A

229 333

1 228 334 389

# 3QVT A

229 333

1 228 334 389

# 3QVS A

229 333

1 228 334 389

# 3QVX A

229 333

1 228 334 389

# 3QVW A

229 333

1 228 334 389

# 1GR0 A

203 311

16 202 312 366

# 1P1K B

324 439

66 323 440 515

# 1P1K A

324 439

66 323 440 515

# 1LA2 B

324 439

66 323 440 515

# 1LA2 A

324 439

66 323 440 515

# 1LA2 D

324 439

66 323 440 515

# 1LA2 C

324 439

66 323 440 515

# 1RM0 B

324 439

66 323 440 515

# 1RM0 A

324 439

66 323 440 515

# 1P1I B

324 439

66 323 440 515

# 1P1I A

324 439

66 323 440 515

# 1JKF A

324 439

66 323 440 515

# 1JKF B

324 439

66 323 440 515

# 1P1F A

324 439

66 323 440 515

# 1P1F B

324 439

66 323 440 515

# 1P1J B

324 439

66 323 440 515

# 1P1J A

324 439

66 323 440 515

# 1JKI B

324 439

66 323 440 515

# 1JKI A

324 439

66 323 440 515

# 1P1H A

324 439

66 323 440 515

# 1P1H C

324 439

66 323 440 515

# 1P1H B

324 439

66 323 440 515

# 1P1H D

324 439

66 323 440 515

### PfamID PF00353 ###

# 1SRP A

341 358

248 331 340 349 350 358 359 368 377 471

# 1SRP A

332 349

248 331 340 349 350 358 359 368 377 471

# 1SRP A

350 367

248 331 340 349 350 358 359 368 377 471

# 1SRP A

359 376

248 331 340 349 350 358 359 368 377 471

# 1SMP A

350 367

248 331 340 349 350 358 359 368 377 471

# 1SMP A

332 349

248 331 340 349 350 358 359 368 377 471

# 1SMP A

359 376

248 331 340 349 350 358 359 368 377 471

# 1SMP A

341 358

248 331 340 349 350 358 359 368 377 471

# 1SAT A

350 367

248 331 340 349 350 358 359 368 377 471

# 1SAT A

332 349

248 331 340 349 350 358 359 368 377 471

# 1SAT A

359 376

248 331 340 349 350 358 359 368 377 471

# 1SAT A

341 358

248 331 340 349 350 358 359 368 377 471

# 1AF0 A

350 367

248 331 340 349 350 358 359 368 377 471

# 1AF0 A

332 349

248 331 340 349 350 358 359 368 377 471

# 1AF0 A

359 376

248 331 340 349 350 358 359 368 377 471

# 1AF0 A

341 358

248 331 340 349 350 358 359 368 377 471

# 1K7Q A

380 397

260 343 361 362 379 380 398 479

# 1K7Q A

362 379

260 343 361 362 379 380 398 479

# 1K7Q A

344 361

260 343 361 362 379 380 398 479

# 1GO8 P

380 397

260 343 361 362 379 380 398 479

# 1GO8 P

362 379

260 343 361 362 379 380 398 479

# 1GO8 P

344 361

260 343 361 362 379 380 398 479

# 3HBV P

380 397

260 343 361 362 379 380 398 479

# 3HBV P

362 379

260 343 361 362 379 380 398 479

# 3HBV P

344 361

260 343 361 362 379 380 398 479

# 3HBU P

380 397

260 343 361 362 379 380 398 479

# 3HBU P

362 379

260 343 361 362 379 380 398 479

# 3HBU P

344 361

260 343 361 362 379 380 398 479

# 3HB2 P

380 397

260 343 361 362 379 380 398 479

# 3HB2 P

362 379

260 343 361 362 379 380 398 479

# 3HB2 P

344 361

260 343 361 362 379 380 398 479

# 1GO7 P

380 397

260 343 361 362 379 380 398 479

# 1GO7 P

362 379

260 343 361 362 379 380 398 479

# 1GO7 P

344 361

260 343 361 362 379 380 398 479

# 1K7G A

380 397

260 343 361 362 379 380 398 479

# 1K7G A

362 379

260 343 361 362 379 380 398 479

# 1K7G A

344 361

260 343 361 362 379 380 398 479

# 3HDA P

380 397

260 343 361 362 379 380 398 479

# 3HDA P

362 379

260 343 361 362 379 380 398 479

# 3HDA P

344 361

260 343 361 362 379 380 398 479

# 1K7I A

380 397

260 343 361 362 379 380 398 479

# 1K7I A

362 379

260 343 361 362 379 380 398 479

# 1K7I A

344 361

260 343 361 362 379 380 398 479

# 1OM7 A

329 346

245 328 346 347 355 365 374 463

# 1OM7 A

356 373

245 328 346 347 355 365 374 463

# 1OM7 A

347 364

245 328 346 347 355 365 374 463

# 1OM8 A

329 346

245 328 346 347 355 365 374 463

# 1OM8 A

356 373

245 328 346 347 355 365 374 463

# 1OM8 A

347 364

245 328 346 347 355 365 374 463

# 1O0T A

329 346

245 328 346 347 355 365 374 463

# 1O0T A

356 373

245 328 346 347 355 365 374 463

# 1O0T A

347 364

245 328 346 347 355 365 374 463

# 1OM6 A

329 346

245 328 346 347 355 365 374 463

# 1OM6 A

356 373

245 328 346 347 355 365 374 463

# 1OM6 A

347 364

245 328 346 347 355 365 374 463

# 1H71 P

329 346

245 328 346 347 355 365 374 463

# 1H71 P

356 373

245 328 346 347 355 365 374 463

# 1H71 P

347 364

245 328 346 347 355 365 374 463

# 1O0Q A

329 346

245 328 346 347 355 365 374 463

# 1O0Q A

356 373

245 328 346 347 355 365 374 463

# 1O0Q A

347 364

245 328 346 347 355 365 374 463

# 1OMJ A

329 346

245 328 346 347 355 365 374 463

# 1OMJ A

356 373

245 328 346 347 355 365 374 463

# 1OMJ A

347 364

245 328 346 347 355 365 374 463

# 1G9K A

329 346

245 328 346 347 355 365 374 463

# 1G9K A

356 373

245 328 346 347 355 365 374 463

# 1G9K A

347 364

245 328 346 347 355 365 374 463

# 1JIW P

350 367

248 345 349 358 359 368 377 470

# 1JIW P

359 376

248 345 349 358 359 368 377 470

# 1JIW P

346 358

248 345 349 358 359 368 377 470

# 1AKL A

350 367

248 345 349 358 359 368 377 470

# 1AKL A

359 376

248 345 349 358 359 368 377 470

# 1AKL A

346 358

248 345 349 358 359 368 377 470

# 1KAP P

350 367

248 345 349 358 359 368 377 470

# 1KAP P

359 376

248 345 349 358 359 368 377 470

# 1KAP P

346 358

248 345 349 358 359 368 377 470

### PfamID PF02710 ###

# 3CL4 A

128 266

19 127 267 376

# 3CL5 A

128 266

19 127 267 376

# 1FLC E

137 292

5 136 293 407

# 1FLC C

137 292

5 136 293 407

# 1FLC A

137 292

5 136 293 407

# 3I1L C

131 271

25 130 272 386

# 3I1L A

131 271

25 130 272 386

# 3I1L B

131 271

25 130 272 386

# 3I1K C

131 271

25 130 272 386

# 3I1K B

131 271

25 130 272 386

# 3I1K A

131 271

25 130 272 386

# 3I26 D

122 263

16 121 264 379

# 3I26 C

122 263

16 121 264 378

# 3I26 B

122 263

16 121 264 379

# 3I26 A

122 263

16 121 264 379

# 3I27 C

122 263

16 121 264 378

# 3I27 D

122 263

16 121 264 379

# 3I27 B

122 263

16 121 264 379

# 3I27 A

122 263

16 121 264 379

### PfamID PF02938 ###

# 1ZQ1 D

301 393

13 300 394 451

# 1ZQ1 C

301 393

13 300 394 451

# 1EQR B

307 406

117 306 407 559

# 1EQR A

307 406

117 306 407 559

# 1EQR C

307 406

117 306 407 559

# 1IL2 B

1307 1406

1117 1306 1407 1559

# 1IL2 A

307 406

117 306 407 559

# 1C0A A

307 406

117 306 407 559

# 2D6F D

289 381

7 288 382 439

# 2D6F C

289 381

7 288 382 439

# 1L0W A

312 400

123 311 401 553

# 1L0W B

1312 1400

1123 1311 1401 1553

# 1G51 B

1312 1400

1123 1311 1401 1553

# 1G51 A

312 400

123 311 401 553

# 1EFW B

312 400

123 311 401 553

# 1EFW A

312 400

123 311 401 553

### PfamID PF07687 ###

# 3PFE A

203 366

92 202 367 466

# 3GB0 A

178 276

73 177 277 369

# 3ISZ A

176 284

63 175 285 373

# 3ISZ B

176 284

63 175 285 373

# 3IC1 A

176 284

63 175 285 373

# 3IC1 B

176 284

63 175 285 373

# 1YSJ A

178 280

69 177 281 373

# 1YSJ B

178 280

69 177 281 373

# 1VIX A

205 309

74 204 310 403

# 1VIX B

205 309

74 204 310 403

# 3DLJ A

215 376

102 214 377 476

# 3DLJ B

215 376

102 214 377 476

# 3MRU A

210 299

76 209 300 485

# 3MRU B

210 299

76 209 300 485

# 3IFE A

208 311

114 207 312 405

# 2F8H A

163 274

68 162 275 364

# 3TX8 A

174 281

73 173 282 369

# 1XMB A

215 317

108 214 318 421

# 2Q43 A

194 296

87 193 297 400

# 3IO1 B

227 327

112 226 328 429

# 3IO1 A

227 327

112 226 328 429

# 3CT9 B

169 275

69 168 276 352

# 3CT9 A

169 275

69 168 276 352

# 2ZOG B

208 369

95 207 370 469

# 2ZOG A

208 369

95 207 370 469

# 2ZOF A

208 369

95 207 370 469

# 2ZOF B

208 369

95 207 370 469

# 2RB7 B

171 272

68 170 273 356

# 2RB7 A

171 272

68 170 273 356

# 1VGY B

177 285

64 176 286 374

# 1VGY A

177 285

64 176 286 374

# 1R3N C

244 347

110 243 348 445

# 1R3N E

244 347

110 243 348 445

# 1R3N H

244 347

110 243 348 445

# 1R3N G

244 347

110 243 348 445

# 1R3N A

244 347

110 243 348 445

# 1R3N F

244 347

110 243 348 445

# 1R3N B

244 347

110 243 348 445

# 1R3N D

244 347

110 243 348 445

# 2VL1 D

244 347

110 243 348 445

# 2VL1 C

244 347

110 243 348 445

# 2VL1 A

244 347

110 243 348 445

# 2VL1 B

244 347

110 243 348 445

# 2V8V C

244 347

110 243 348 445

# 2V8V A

244 347

110 243 348 445

# 2V8V B

244 347

110 243 348 445

# 2V8V D

244 347

110 243 348 445

# 1R43 B

244 347

110 243 348 445

# 1R43 A

244 347

110 243 348 445

# 2V8G B

244 347

110 243 348 445

# 2V8G C

244 347

110 243 348 445

# 2V8G A

244 347

110 243 348 445

# 2V8G D

244 347

110 243 348 445

# 2V8H D

244 347

110 243 348 445

# 2V8H C

244 347

110 243 348 445

# 2V8H B

244 347

110 243 348 445

# 2V8H A

244 347

110 243 348 445

# 2V8D A

244 347

110 243 348 445

# 2V8D B

244 347

110 243 348 445

# 1FNO A

205 309

74 204 310 403

# 3N5F A

207 312

75 206 313 404

# 3N5F B

207 312

75 206 313 404

# 2F7V A

163 274

68 162 275 364

# 2QYV B

205 296

72 204 297 482

# 2QYV A

205 296

72 204 297 482

# 3RZA B

182 277

75 181 278 371

# 3RZA A

182 277

75 181 278 371

# 2POK A

198 360

86 197 361 455

# 2POK B

198 360

86 197 361 455

# 1CG2 D

211 314

108 210 315 410

# 1CG2 B

211 314

108 210 315 410

# 1CG2 A

211 314

108 210 315 410

# 1CG2 C

211 314

108 210 315 410

# 3PFO B

209 325

106 208 326 425

# 3PFO A

209 325

106 208 326 425

### PfamID PF14528 ###

# 2CW7 A

373 442

34 372 443 478

# 2CW8 A

373 442

34 372 443 478

### PfamID PF06470 ###

# 3NWC B

519 629

488 518 630 662

# 3NWC A

519 629

488 518 630 667

# 2WD5 B

528 642

492 527 643 685

# 1GXL C

513 630

475 512 631 679

# 1GXL B

513 630

475 512 631 679

# 1GXL A

513 630

475 512 631 679

# 1GXL D

513 630

482 512 631 679

### PfamID PF00040 ###

# 1L6J A

230 271

115 229 272 287 330 346 389 444

# 1L6J A

288 329

115 229 272 287 330 346 389 444

# 1L6J A

347 388

115 229 272 287 330 346 389 444

# 1GXD A

262 303

89 203 246 261 304 319 362 417

# 1GXD A

204 245

89 203 246 261 304 319 362 417

# 1GXD A

320 361

89 203 246 261 304 319 362 417

# 1GXD B

262 303

89 203 246 261 304 319 362 417

# 1GXD B

204 245

89 203 246 261 304 319 362 417

# 1GXD B

320 361

89 203 246 261 304 319 362 417

# 1EAK C

291 332

118 232 275 290 333 348 391 446

# 1EAK C

233 274

118 232 275 290 333 348 391 446

# 1EAK C

349 390

118 232 275 290 333 348 391 446

# 1EAK B

291 332

118 232 275 290 333 348 391 446

# 1EAK B

233 274

118 232 275 290 333 348 391 446

# 1EAK B

349 390

118 232 275 290 333 348 391 446

# 1EAK D

291 332

118 232 275 290 333 348 391 446

# 1EAK D

233 274

118 232 275 290 333 348 391 446

# 1EAK D

349 390

118 232 275 290 333 348 391 446

# 1EAK A

291 332

118 232 275 290 333 348 391 446

# 1EAK A

233 274

118 232 275 290 333 348 391 446

# 1EAK A

349 390

118 232 275 290 333 348 391 446

# 1CK7 A

291 332

118 232 275 290 333 348 391 446

# 1CK7 A

233 274

118 232 275 290 333 348 391 446

# 1CK7 A

349 390

118 232 275 290 333 348 391 446

### PfamID PF07691 ###

# 3ABZ D

409 554

321 408 555 722

# 3ABZ C

409 554

321 408 555 722

# 3ABZ B

409 554

321 408 555 722

# 3ABZ A

409 554

321 408 555 722

# 3AC0 D

409 554

321 408 555 722

# 3AC0 C

409 554

321 408 555 722

# 3AC0 B

409 554

321 408 555 722

# 3AC0 A

409 554

321 408 555 722

### PfamID PF00571 ###

# 1JR1 A

112 155

28 111 156 177 233 504

# 1JR1 A

178 232

28 111 156 177 233 504

# 1ZFJ A

88 149

11 87 150 154 212 479

# 1ZFJ A

155 211

11 87 150 154 212 479

# 1JCN B

181 232

28 111 165 180 233 499

# 1JCN B

112 164

28 111 165 180 233 499

# 1JCN A

181 232

28 111 165 180 233 499

# 1JCN A

112 164

28 111 165 180 233 499

# 1NFB B

188 232

28 111 153 187 233 498

# 1NFB B

112 152

28 111 153 187 233 498

# 1NFB A

188 232

28 111 153 187 233 498

# 1NFB A

112 152

28 111 153 187 233 498

# 1NF7 B

175 232

28 111 166 174 233 504

# 1NF7 B

112 165

28 111 166 174 233 504

# 1NF7 A

175 232

28 111 166 174 233 504

# 1NF7 A

112 165

28 111 166 174 233 504

# 1B3O B

178 232

28 111 160 177 233 499

# 1B3O B

112 159

28 111 160 177 233 499

# 3USB B

92 146

9 91 147 152 210 469

# 3USB B

153 209

9 91 147 152 210 469

# 3USB A

92 148

9 91 149 152 210 469

# 3USB A

153 209

9 91 149 152 210 469

# 3TSD A

92 148

9 91 149 152 210 473

# 3TSD A

153 209

9 91 149 152 210 473

# 3TSD B

92 146

9 91 147 152 210 472

# 3TSD B

153 209

9 91 147 152 210 472

# 3TSB B

92 146

9 91 147 161 210 474

# 3TSB B

162 209

9 91 147 161 210 474

# 3TSB A

92 148

9 91 149 152 210 474

# 3TSB A

153 209

9 91 149 152 210 474

### PfamID PF00627 ###

# 3IHP A

634 669

325 633 670 698 736 830

# 3IHP A

699 735

325 633 670 698 736 830

# 3IHP B

632 669

325 631 670 699 737 830

# 3IHP B

700 736

325 631 670 699 737 830

### PfamID PF01753 ###

# 3N71 A

52 90

18 51 91 253

# 3RU0 A

47 87

15 46 88 240

# 3RU0 B

47 87

15 46 88 240

# 3MEK A

47 87

15 46 88 240

# 3OXL A

47 87

15 46 88 240

# 3PDN A

47 87

15 46 88 240

# 3QWP A

47 87

15 46 88 240

# 3OXF B

47 87

15 46 88 240

# 3OXF A

47 87

15 46 88 240

# 3OXG A

47 87

15 46 88 240

# 3TG5 A

52 90

18 51 91 241

# 3S7F A

52 90

18 51 91 241

# 3RIB B

52 90

18 51 91 241

# 3RIB A

52 90

18 51 91 241

# 3TG4 A

52 90

18 51 91 241

# 3S7J A

52 90

18 51 91 241

# 3S7D A

52 90

18 51 91 241

# 3S7B A

52 90

18 51 91 241

# 3QWW A

51 90

18 50 91 241

# 3QWV A

51 90

18 50 91 241

### PfamID PF00633 ###

# 1KG7 A

99 128

35 98 129 170

# 1KG2 A

99 128

35 98 129 170

# 1MUN A

99 128

35 98 129 170

# 1MUD A

99 128

35 98 129 170

# 1KQJ A

99 128

35 98 129 170

# 1KG5 A

99 128

35 98 129 170

# 1KG4 A

99 128

35 98 129 170

# 1KG6 A

99 128

35 98 129 170

# 1KG3 A

99 128

35 98 129 170

# 1WEI A

99 128

35 98 129 170

# 1WEG A

99 128

35 98 129 170

# 1WEF A

99 128

35 98 129 170

# 1MUY A

99 128

35 98 129 170

# 1KEA A

105 134

40 104 135 178

# 1VRL A

105 134

41 104 135 176

# 1RRQ A

105 134

41 104 135 176

# 3G0Q A

105 134

41 104 135 176

# 3FSQ A

105 134

41 104 135 176

# 1RRS A

105 134

41 104 135 176

# 3FSP A

105 134

41 104 135 176

# 2ABK A

99 128

34 98 129 169

### PfamID PF04992 ###

# 3H0G M

879 1059

813 878 1060 1143 1281 1404

# 3H0G A

879 1059

813 878 1060 1143 1281 1404

# 1Y77 A

873 1056

807 872 1057 1140 1275 1398

# 3CQZ A

873 1056

807 872 1057 1140 1275 1398

# 3GTM A

873 1056

807 872 1057 1140 1275 1398

# 1TWA A

873 1056

807 872 1057 1140 1275 1385

# 4A3I A

873 1056

807 872 1057 1140 1275 1398

# 2NVY A

873 1056

807 872 1057 1140 1275 1398

# 2NVT A

873 1056

807 872 1057 1140 1275 1398

# 1I6H A

873 1056

807 872 1057 1140 1275 1398

# 1TWF A

873 1056

807 872 1057 1140 1275 1398

# 3S1M A

873 1056

807 872 1057 1140 1275 1398

# 2NVQ A

873 1056

807 872 1057 1140 1275 1398

# 3QT1 A

873 1056

807 872 1057 1140 1275 1398

# 2JA7 A

873 1056

807 872 1057 1140 1275 1398

# 2JA7 M

873 1056

807 872 1057 1140 1275 1398

# 3K1F A

873 1056

807 872 1057 1140 1275 1398

# 1I50 A

873 1056

807 872 1057 1140 1275 1398

# 4A3B A

873 1056

807 872 1057 1140 1275 1398

# 3H3V B

873 1056

807 872 1057 1140 1275 1398

# 3GTL A

873 1056

807 872 1057 1140 1275 1398

# 3S2H A

873 1056

807 872 1057 1140 1275 1398

# 3S17 A

873 1056

807 872 1057 1140 1275 1398

# 2VUM A

873 1056

807 872 1057 1140 1275 1398

# 3I4M A

873 1056

807 872 1057 1140 1275 1398

# 2B8K A

873 1056

807 872 1057 1140 1275 1398

# 2YU9 A

873 1056

807 872 1057 1140 1275 1398

# 3RZD A

873 1056

807 872 1057 1140 1275 1398

# 4A3D A

873 1056

807 872 1057 1140 1275 1398

# 3S14 A

873 1056

807 872 1057 1140 1275 1398

# 4A3E A

873 1056

807 872 1057 1140 1275 1398

# 1TWC A

873 1056

807 872 1057 1140 1275 1385

# 3HOX A

873 1056

807 872 1057 1140 1275 1398

# 3S16 A

873 1056

807 872 1057 1140 1275 1398

# 3S1R A

873 1056

807 872 1057 1140 1275 1398

# 2B63 A

873 1056

807 872 1057 1140 1275 1398

# 4A3K A

873 1056

807 872 1057 1140 1275 1398

# 1Y1V A

873 1056

807 872 1057 1140 1275 1398

# 3M4O A

873 1056

807 872 1057 1140 1275 1398

# 3HOY A

873 1056

807 872 1057 1140 1275 1398

# 4A3C A

873 1056

807 872 1057 1140 1275 1398

# 4A3G A

873 1056

807 872 1057 1140 1275 1398

# 3K7A A

873 1056

807 872 1057 1140 1275 1398

# 3PO3 A

873 1056

807 872 1057 1140 1275 1398

# 4A3M A

873 1056

807 872 1057 1140 1275 1398

# 1NIK A

873 1056

807 872 1057 1140 1275 1398

# 3HOZ A

873 1056

807 872 1057 1140 1275 1398

# 3HOV A

873 1056

807 872 1057 1140 1275 1398

# 1R9T A

873 1056

807 872 1057 1140 1275 1398

# 3GTJ A

873 1056

807 872 1057 1140 1275 1398

# 2R93 A

873 1056

807 872 1057 1140 1275 1398

# 3GTP A

873 1056

807 872 1057 1140 1275 1398

# 3I4N A

873 1056

807 872 1057 1140 1275 1398

# 3GTO A

873 1056

807 872 1057 1140 1275 1398

# 1TWG A

873 1056

807 872 1057 1140 1275 1385

# 3M3Y A

873 1056

807 872 1057 1140 1275 1398

# 3GTK A

873 1056

807 872 1057 1140 1275 1398

# 3S1Q A

873 1056

807 872 1057 1140 1275 1398

# 3FKI A

873 1056

807 872 1057 1140 1275 1398

# 2JA5 A

873 1056

807 872 1057 1140 1275 1398

# 3S2D A

873 1056

807 872 1057 1140 1275 1398

# 2E2H A

873 1056

807 872 1057 1140 1275 1398

# 2JA8 A

873 1056

807 872 1057 1140 1275 1398

# 4A3J A

873 1056

807 872 1057 1140 1275 1398

# 3HOU A

873 1056

807 872 1057 1140 1275 1398

# 3HOU M

873 1056

807 872 1057 1140 1275 1398

# 2R92 A

873 1056

807 872 1057 1140 1275 1398

# 1Y1W A

873 1056

807 872 1057 1140 1275 1398

# 2JA6 A

873 1056

807 872 1057 1140 1275 1398

# 1K83 A

873 1056

807 872 1057 1140 1275 1398

# 1R5U A

873 1056

807 872 1057 1140 1275 1398

# 1R9S A

873 1056

807 872 1057 1140 1275 1398

# 1WCM A

873 1056

807 872 1057 1140 1275 1398

# 2NVZ A

873 1056

807 872 1057 1140 1275 1398

# 1I3Q A

873 1056

807 872 1057 1140 1275 1398

# 2R7Z A

873 1056

807 872 1057 1140 1275 1398

# 2E2I A

873 1056

807 872 1057 1140 1275 1398

# 1TWH A

873 1056

807 872 1057 1140 1275 1385

# 3GTQ A

873 1056

807 872 1057 1140 1275 1398

# 4A93 A

873 1056

807 872 1057 1140 1275 1398

# 3GTG A

873 1056

807 872 1057 1140 1275 1398

# 3PO2 A

873 1056

807 872 1057 1140 1275 1398

# 1SFO A

873 1056

807 872 1057 1140 1275 1398

# 3HOW A

873 1056

807 872 1057 1140 1275 1398

# 2NVX A

873 1056

807 872 1057 1140 1275 1398

# 2E2J A

873 1056

807 872 1057 1140 1275 1398

# 4A3F A

873 1056

807 872 1057 1140 1275 1398

# 3S1N A

873 1056

807 872 1057 1140 1275 1398

# 4A3L A

873 1056

807 872 1057 1140 1275 1398

# 3S15 A

873 1056

807 872 1057 1140 1275 1398

### PfamID PF04990 ###

# 1Y77 A

1141 1274

807 872 1057 1140 1275 1398

# 3CQZ A

1141 1274

807 872 1057 1140 1275 1398

# 3GTM A

1141 1274

807 872 1057 1140 1275 1398

# 1TWA A

1141 1274

807 872 1057 1140 1275 1385

# 4A3I A

1141 1274

807 872 1057 1140 1275 1398

# 2NVY A

1141 1274

807 872 1057 1140 1275 1398

# 2NVT A

1141 1274

807 872 1057 1140 1275 1398

# 1I6H A

1141 1274

807 872 1057 1140 1275 1398

# 1TWF A

1141 1274

807 872 1057 1140 1275 1398

# 3S1M A

1141 1274

807 872 1057 1140 1275 1398

# 2NVQ A

1141 1274

807 872 1057 1140 1275 1398

# 3QT1 A

1141 1274

807 872 1057 1140 1275 1398

# 2JA7 A

1141 1274

807 872 1057 1140 1275 1398

# 2JA7 M

1141 1274

807 872 1057 1140 1275 1398

# 3K1F A

1141 1274

807 872 1057 1140 1275 1398

# 1I50 A

1141 1274

807 872 1057 1140 1275 1398

# 4A3B A

1141 1274

807 872 1057 1140 1275 1398

# 3H3V B

1141 1274

807 872 1057 1140 1275 1398

# 3GTL A

1141 1274

807 872 1057 1140 1275 1398

# 3S2H A

1141 1274

807 872 1057 1140 1275 1398

# 3S17 A

1141 1274

807 872 1057 1140 1275 1398

# 2VUM A

1141 1274

807 872 1057 1140 1275 1398

# 3I4M A

1141 1274

807 872 1057 1140 1275 1398

# 2B8K A

1141 1274

807 872 1057 1140 1275 1398

# 2YU9 A

1141 1274

807 872 1057 1140 1275 1398

# 3RZD A

1141 1274

807 872 1057 1140 1275 1398

# 4A3D A

1141 1274

807 872 1057 1140 1275 1398

# 3S14 A

1141 1274

807 872 1057 1140 1275 1398

# 4A3E A

1141 1274

807 872 1057 1140 1275 1398

# 1TWC A

1141 1274

807 872 1057 1140 1275 1385

# 3HOX A

1141 1274

807 872 1057 1140 1275 1398

# 3S16 A

1141 1274

807 872 1057 1140 1275 1398

# 3S1R A

1141 1274

807 872 1057 1140 1275 1398

# 2B63 A

1141 1274

807 872 1057 1140 1275 1398

# 4A3K A

1141 1274

807 872 1057 1140 1275 1398

# 1Y1V A

1141 1274

807 872 1057 1140 1275 1398

# 3M4O A

1141 1274

807 872 1057 1140 1275 1398

# 3HOY A

1141 1274

807 872 1057 1140 1275 1398

# 4A3C A

1141 1274

807 872 1057 1140 1275 1398

# 4A3G A

1141 1274

807 872 1057 1140 1275 1398

# 3K7A A

1141 1274

807 872 1057 1140 1275 1398

# 3PO3 A

1141 1274

807 872 1057 1140 1275 1398

# 4A3M A

1141 1274

807 872 1057 1140 1275 1398

# 1NIK A

1141 1274

807 872 1057 1140 1275 1398

# 3HOZ A

1141 1274

807 872 1057 1140 1275 1398

# 3HOV A

1141 1274

807 872 1057 1140 1275 1398

# 1R9T A

1141 1274

807 872 1057 1140 1275 1398

# 3GTJ A

1141 1274

807 872 1057 1140 1275 1398

# 2R93 A

1141 1274

807 872 1057 1140 1275 1398

# 3GTP A

1141 1274

807 872 1057 1140 1275 1398

# 3I4N A

1141 1274

807 872 1057 1140 1275 1398

# 3GTO A

1141 1274

807 872 1057 1140 1275 1398

# 1TWG A

1141 1274

807 872 1057 1140 1275 1385

# 3M3Y A

1141 1274

807 872 1057 1140 1275 1398

# 3GTK A

1141 1274

807 872 1057 1140 1275 1398

# 3S1Q A

1141 1274

807 872 1057 1140 1275 1398

# 3FKI A

1141 1274

807 872 1057 1140 1275 1398

# 2JA5 A

1141 1274

807 872 1057 1140 1275 1398

# 3S2D A

1141 1274

807 872 1057 1140 1275 1398

# 2E2H A

1141 1274

807 872 1057 1140 1275 1398

# 2JA8 A

1141 1274

807 872 1057 1140 1275 1398

# 4A3J A

1141 1274

807 872 1057 1140 1275 1398

# 3HOU A

1141 1274

807 872 1057 1140 1275 1398

# 3HOU M

1141 1274

807 872 1057 1140 1275 1398

# 2R92 A

1141 1274

807 872 1057 1140 1275 1398

# 1Y1W A

1141 1274

807 872 1057 1140 1275 1398

# 2JA6 A

1141 1274

807 872 1057 1140 1275 1398

# 1K83 A

1141 1274

807 872 1057 1140 1275 1398

# 1R5U A

1141 1274

807 872 1057 1140 1275 1398

# 1R9S A

1141 1274

807 872 1057 1140 1275 1398

# 1WCM A

1141 1274

807 872 1057 1140 1275 1398

# 2NVZ A

1141 1274

807 872 1057 1140 1275 1398

# 1I3Q A

1141 1274

807 872 1057 1140 1275 1398

# 2R7Z A

1141 1274

807 872 1057 1140 1275 1398

# 2E2I A

1141 1274

807 872 1057 1140 1275 1398

# 1TWH A

1141 1274

807 872 1057 1140 1275 1385

# 3GTQ A

1141 1274

807 872 1057 1140 1275 1398

# 4A93 A

1141 1274

807 872 1057 1140 1275 1398

# 3GTG A

1141 1274

807 872 1057 1140 1275 1398

# 3PO2 A

1141 1274

807 872 1057 1140 1275 1398

# 1SFO A

1141 1274

807 872 1057 1140 1275 1398

# 3HOW A

1141 1274

807 872 1057 1140 1275 1398

# 2NVX A

1141 1274

807 872 1057 1140 1275 1398

# 2E2J A

1141 1274

807 872 1057 1140 1275 1398

# 4A3F A

1141 1274

807 872 1057 1140 1275 1398

# 3S1N A

1141 1274

807 872 1057 1140 1275 1398

# 4A3L A

1141 1274

807 872 1057 1140 1275 1398

# 3S15 A

1141 1274

807 872 1057 1140 1275 1398

# 3H0G M

1144 1280

813 878 1060 1143 1281 1404

# 3H0G A

1144 1280

813 878 1060 1143 1281 1404

### PfamID PF00070 ###

# 2VDC K

291 369

149 290 370 448

# 2VDC H

291 369

149 290 370 448

# 2VDC I

291 369

149 290 370 448

# 2VDC L

291 369

149 290 370 448

# 2VDC G

291 369

149 290 370 448

# 2VDC J

291 369

149 290 370 448

# 1XDI B

184 264

4 183 265 322

# 1XDI A

184 264

4 183 265 322

# 2V3A A

147 227

6 146 228 283

# 2V3B A

147 227

6 146 228 283

# 1XHC A

137 211

2 136 212 266

# 3GD4 A

301 385

133 300 386 443

# 3GD4 B

301 385

133 300 386 443

# 3GD3 A

301 385

133 300 386 443

# 3GD3 D

301 385

133 300 386 443

# 3GD3 C

301 385

133 300 386 443

# 3GD3 B

301 385

133 300 386 443

# 1GV4 B

301 385

133 300 386 443

# 1GV4 A

301 385

133 300 386 443

# 1ZMD D

180 264

8 179 265 326

# 1ZMD F

180 264

8 179 265 326

# 1ZMD G

180 264

8 179 265 326

# 1ZMD A

180 264

8 179 265 326

# 1ZMD E

180 264

8 179 265 326

# 1ZMD C

180 264

8 179 265 326

# 1ZMD H

180 264

8 179 265 326

# 1ZMD B

180 264

8 179 265 326

# 1ZMC H

180 264

8 179 265 326

# 1ZMC A

180 264

8 179 265 326

# 1ZMC G

180 264

8 179 265 326

# 1ZMC E

180 264

8 179 265 326

# 1ZMC D

180 264

8 179 265 326

# 1ZMC B

180 264

8 179 265 326

# 1ZMC C

180 264

8 179 265 326

# 1ZMC F

180 264

8 179 265 326

# 3RNM A

180 264

8 179 265 326

# 3RNM B

180 264

8 179 265 326

# 3RNM C

180 264

8 179 265 326

# 3RNM D

180 264

8 179 265 326

# 2F5Z H

180 264

8 179 265 326

# 2F5Z B

180 264

8 179 265 326

# 2F5Z A

180 264

8 179 265 326

# 2F5Z E

180 264

8 179 265 326

# 2F5Z G

180 264

8 179 265 326

# 2F5Z J

180 264

8 179 265 326

# 2F5Z I

180 262

8 179 263 326

# 2F5Z C

180 264

8 179 265 326

# 2F5Z F

180 264

8 179 265 326

# 2F5Z D

180 264

8 179 265 326

# 1ZY8 H

180 264

8 179 265 326

# 1ZY8 A

180 264

8 179 265 326

# 1ZY8 G

180 264

8 179 265 326

# 1ZY8 J

180 264

8 179 265 326

# 1ZY8 F

180 264

8 179 265 326

# 1ZY8 C

180 264

8 179 265 326

# 1ZY8 D

180 264

8 179 265 326

# 1ZY8 E

180 264

8 179 265 326

# 1ZY8 B

180 264

8 179 265 326

# 1ZY8 I

180 264

8 179 265 326

# 1NDA A

190 275

6 189 276 332

# 1NDA B

190 275

6 189 276 332

# 1NDA D

190 275

6 189 276 332

# 1NDA C

190 275

6 189 276 332

# 1GXF B

191 276

7 190 277 333

# 1GXF A

191 276

7 190 277 333

# 1BZL A

191 276

7 190 277 333

# 1BZL B

191 276

7 190 277 333

# 1AOG A

191 276

7 190 277 333

# 1AOG B

191 276

7 190 277 333

# 3EF6 A

145 225

4 144 226 281

# 2WBA B

190 274

6 189 275 332

# 2WBA A

190 274

6 189 275 332

# 3URH A

177 261

4 176 262 321

# 3URH B

177 261

4 176 262 321

# 3KLJ A

142 218

5 141 219 265

# 1GET B

169 250

6 168 251 308

# 1GET A

169 250

6 168 251 308

# 1GES A

169 250

6 168 251 308

# 1GES B

169 250

6 168 251 308

# 1GER A

169 250

6 168 251 308

# 1GER B

169 250

6 168 251 308

# 1GEU B

169 250

6 168 251 308

# 1GEU A

169 250

6 168 251 308

# 3O0H A

172 252

7 171 253 311

# 3O0H B

172 252

7 171 253 311

# 1YQZ B

150 228

3 149 229 283

# 1YQZ A

150 228

3 149 229 283

# 3LXD A

154 235

11 153 236 291

# 2WP5 B

190 274

6 189 275 332

# 2WP5 D

190 274

6 189 275 332

# 2WP5 C

190 274

6 189 275 332

# 2WP5 A

190 274

6 189 275 332

# 2WOV A

190 274

6 189 275 332

# 2WOV C

190 274

6 189 275 332

# 2WOV B

190 274

6 189 275 332

# 2WOV D

190 274

6 189 275 332

# 2WPE A

190 274

6 189 275 332

# 2WPE B

190 274

6 189 275 332

# 2WPE D

190 274

6 189 275 332

# 2WPE C

190 274

6 189 275 332

# 2WPC A

190 274

6 189 275 332

# 2WPC D

190 274

6 189 275 332

# 2WPC C

190 274

6 189 275 332

# 2WPC B

190 274

6 189 275 332

# 2WOW B

190 274

6 189 275 332

# 2WOW D

190 274

6 189 275 332

# 2WOW C

190 274

6 189 275 332

# 2WOW A

190 274

6 189 275 332

# 2WOI D

190 274

6 189 275 332

# 2WOI A

190 274

6 189 275 332

# 2WOI B

190 274

6 189 275 332

# 2WOI C

190 274

6 189 275 332

# 2WP6 D

190 274

6 189 275 332

# 2WP6 C

190 274

6 189 275 332

# 2WP6 A

190 274

6 189 275 332

# 2WP6 B

190 274

6 189 275 332

# 2WPF C

190 274

6 189 275 332

# 2WPF B

190 274

6 189 275 332

# 2WPF D

190 274

6 189 275 332

# 2WPF A

190 274

6 189 275 332

# 3L8K B

172 252

4 171 253 311

# 3L8K A

172 252

4 171 253 311

# 1EBD A

178 260

11 177 261 319

# 1EBD B

178 260

11 177 261 319

# 3AB1 A

165 245

16 164 246 303

# 3AB1 B

165 245

16 164 246 303

# 1H6V E

192 276

14 191 277 340

# 1H6V F

192 276

14 191 277 340

# 1H6V C

192 276

14 191 277 340

# 1H6V B

192 276

14 191 277 340

# 1H6V A

192 276

14 191 277 340

# 1H6V D

192 276

14 191 277 340

# 3R9U B

146 224

3 145 225 286

# 3R9U A

146 224

3 145 225 286

# 1VDC A

147 225

7 146 226 292

# 1ONF A

177 257

3 176 258 316

# 3FG2 P

144 225

3 143 226 281

# 2YVG A

147 223

9 146 224 279

# 2GR1 A

147 223

9 146 224 279

# 2GQW A

147 223

9 146 224 279

# 2GR3 A

147 223

9 146 224 279

# 2YVF A

147 223

9 146 224 279

# 1F3P A

147 223

9 146 224 279

# 2GR0 A

147 223

9 146 224 279

# 2GR2 A

147 223

9 146 224 279

# 2YVJ P

147 223

9 146 224 279

# 2YVJ A

147 223

9 146 224 279

# 1D7Y A

147 223

9 146 224 279

# 2A87 B

157 233

16 156 234 294

# 2A87 A

157 233

16 156 234 294

# 3ITJ C

155 233

4 154 234 293

# 3ITJ B

155 233

4 154 234 293

# 3ITJ A

155 233

4 154 234 293

# 3ITJ D

155 233

4 154 234 293

# 3D8X B

156 234

5 155 235 294

# 3D8X A

156 234

5 155 235 294

# 3H4K A

288 365

109 287 366 439

# 2V6O A

288 365

109 287 366 439

# 2X8H A

288 365

109 287 366 439

# 2X8C A

288 365

109 287 366 439

# 2X8C B

288 365

109 287 366 439

# 2X99 A

288 365

109 287 366 439

# 2X8G A

288 365

109 287 366 439

# 3II4 A

173 253

5 172 254 314

# 3II4 B

173 253

5 172 254 314

# 2A8X A

173 253

5 172 254 314

# 2A8X B

173 253

5 172 254 314

# 1F6M F

147 228

7 146 229 292

# 1F6M A

147 228

7 146 229 292

# 1F6M B

147 228

7 146 229 292

# 1F6M E

147 228

7 146 229 292

# 1TRB A

147 228

7 146 229 292

# 1CL0 A

147 228

7 146 229 292

# 1TDF A

147 228

7 146 229 292

# 1TDE A

147 228

7 146 229 292

# 1M6I A

302 386

134 301 387 444

# 3OC4 A

147 228

2 146 229 283

# 3OC4 B

147 228

2 146 229 283

# 3DGH A

189 272

11 188 273 332

# 3DGH B

189 272

11 188 273 332

# 2NVK X

189 272

11 188 273 332

# 3DH9 B

189 272

11 188 273 332

# 3DH9 A

189 272

11 188 273 332

# 1FEA C

189 274

5 188 275 332

# 1FEA B

189 274

5 188 275 332

# 1FEA A

189 274

5 188 275 332

# 1FEA D

189 274

5 188 275 332

# 1TYP B

190 275

6 189 276 333

# 1TYP A

190 275

6 189 276 333

# 1FEC B

189 274

5 188 275 332

# 1FEC A

189 274

5 188 275 332

# 1TYT B

190 275

6 189 276 333

# 1TYT A

190 275

6 189 276 333

# 1FEB B

189 274

5 188 275 332

# 1FEB A

189 274

5 188 275 332

# 2TPR A

189 274

5 188 275 332

# 2TPR B

189 274

5 188 275 332

# 3CTY B

157 235

18 156 236 294

# 3CTY A

157 235

18 156 236 294

# 1HYU A

357 432

214 356 433 494

# 2Q7V B

154 232

10 153 233 290

# 2Q7V A

154 232

10 153 233 290

# 2QAE B

176 255

4 175 256 320

# 2QAE A

176 255

4 175 256 320

# 2C3C A

216 299

45 215 300 359

# 2C3C B

216 299

45 215 300 359

# 1MOK D

216 299

45 215 300 359

# 1MOK B

216 299

45 215 300 359

# 1MOK A

216 299

45 215 300 359

# 1MOK C

216 299

45 215 300 359

# 2C3D A

216 299

45 215 300 359

# 2C3D B

216 299

45 215 300 359

# 3Q6J B

216 299

45 215 300 359

# 3Q6J A

216 299

45 215 300 359

# 1MO9 B

216 299

45 215 300 359

# 1MO9 A

216 299

45 215 300 359

# 2WHD A

159 240

12 158 241 299

# 2WHD B

159 240

12 158 241 299

# 1ZK7 A

178 257

6 177 258 315

# 1ZX9 A

178 257

6 177 258 315

# 2Q0L A

145 224

3 144 225 286

# 2Q0L B

145 224

3 144 225 286

# 3ISH B

145 224

3 144 225 286

# 3ISH C

145 224

3 144 225 286

# 3ISH A

145 224

3 144 225 286

# 2Q0K A

145 224

3 144 225 286

# 2Q0K B

145 224

3 144 225 286

# 4DNA B

172 254

7 171 255 312

# 4DNA A

172 254

7 171 255 312

# 2YQU A

169 249

3 168 250 308

# 2YQU B

169 249

3 168 250 308

# 2EQ7 B

169 249

3 168 250 308

# 2EQ7 A

169 249

3 168 250 308

# 2R9Z B

168 247

6 167 248 308

# 2R9Z A

168 247

6 167 248 308

# 2RAB A

168 247

6 167 248 308

# 2RAB B

168 247

6 167 248 308

# 1GRA A

189 268

22 188 269 336

# 1GRT A

189 268

22 188 269 336

# 5GRT A

189 268

22 188 269 336

# 3GRS A

189 268

22 188 269 336

# 1GRE A

189 268

22 188 269 336

# 3DK4 A

189 268

22 188 269 336

# 4GRT A

189 268

22 188 269 336

# 4GR1 A

189 268

22 188 269 336

# 2AAQ A

189 268

22 188 269 336

# 3DK9 A

189 268

22 188 269 336

# 3DJG X

189 268

22 188 269 336

# 2GRT A

189 268

22 188 269 336

# 3DK8 A

189 268

22 188 269 336

# 3SQP B

189 268

22 188 269 336

# 3SQP A

189 268

22 188 269 336

# 1GRF A

189 268

22 188 269 336

# 2GH5 A

189 268

22 188 269 336

# 2GH5 B

189 268

22 188 269 336

# 3DJJ A

189 268

22 188 269 336

# 1XAN A

189 268

22 188 269 336

# 3GRT A

189 268

22 188 269 336

# 1GRB A

189 268

22 188 269 336

# 1BWC A

189 268

22 188 269 336

# 1OJT A

303 386

124 302 387 446

# 1BHY A

303 386

124 302 387 446

# 2W0H A

190 275

6 189 276 333

# 2W0H B

190 275

6 189 276 333

# 2X50 B

190 275

6 189 276 333

# 2X50 A

190 275

6 189 276 333

# 2YAU A

190 275

6 189 276 333

# 2YAU B

190 275

6 189 276 333

# 2JK6 B

190 275

6 189 276 333

# 2JK6 A

190 275

6 189 276 333

# 1Q1W A

151 234

6 150 235 290

# 1Q1W B

151 234

6 150 235 290

# 3LB8 A

151 234

6 150 235 290

# 3LB8 B

151 234

6 150 235 290

# 1Q1R B

151 234

6 150 235 290

# 1Q1R A

151 234

6 150 235 290

# 1ZKQ A

220 302

41 219 303 365

# 3DGZ A

187 269

8 186 270 332

# 1ZDL A

220 302

41 219 303 365

# 1FL2 A

357 432

214 356 433 494

# 1LVL A

173 257

7 172 258 311

# 3ICR B

155 233

4 154 234 290

# 3ICR A

155 233

4 154 234 290

# 3ICT A

155 233

4 154 234 290

# 3ICT B

155 233

4 154 234 290

# 3ICS B

155 233

4 154 234 290

# 3ICS A

155 233

4 154 234 290

# 2HQM B

199 281

25 198 282 340

# 2HQM A

199 281

25 198 282 340

# 1V59 B

185 270

7 184 271 331

# 1V59 A

185 270

7 184 271 331

# 1JEH A

185 270

7 184 271 331

# 1JEH B

185 270

7 184 271 331

# 3NT6 B

153 230

3 152 231 309

# 3NT6 A

153 230

3 152 231 309

# 3NTA B

153 230

3 152 231 309

# 3NTA A

153 230

3 152 231 309

# 3NTD A

153 230

3 152 231 309

# 3NTD B

153 230

3 152 231 309

# 2CFY D

192 272

14 191 273 340

# 2CFY A

192 272

14 191 273 340

# 2CFY F

192 272

14 191 273 340

# 2CFY B

192 272

14 191 273 340

# 2CFY C

192 272

14 191 273 340

# 2CFY E

192 272

14 191 273 340

# 2ZZC D

192 272

14 191 273 340

# 2ZZC B

192 272

14 191 273 340

# 2ZZC C

192 272

14 191 273 340

# 2ZZC A

192 272

14 191 273 340

# 2J3N D

192 272

14 191 273 340

# 2J3N E

192 272

14 191 273 340

# 2J3N B

192 272

14 191 273 340

# 2J3N A

192 272

14 191 273 340

# 2J3N F

192 272

14 191 273 340

# 2J3N C

192 272

14 191 273 340

# 2ZZ0 C

192 272

14 191 273 340

# 2ZZ0 D

192 272

14 191 273 340

# 2ZZ0 A

192 272

14 191 273 340

# 2ZZ0 B

192 272

14 191 273 340

# 2ZZB A

192 272

14 191 273 340

# 2ZZB D

192 272

14 191 273 340

# 2ZZB B

192 272

14 191 273 340

# 2ZZB C

192 272

14 191 273 340

# 3QFB B

192 272

14 191 273 340

# 3QFB A

192 272

14 191 273 340

# 3QFA B

192 272

14 191 273 340

# 3QFA A

192 272

14 191 273 340

# 1DXL C

179 262

8 178 263 323

# 1DXL D

179 262

8 178 263 323

# 1DXL A

179 262

8 178 263 323

# 1DXL B

179 262

8 178 263 323

### PfamID PF05191 ###

# 1ZIP A

127 162

5 126 163 191

# 1ZIO A

127 162

5 126 163 191

# 1ZIN A

127 162

5 126 163 191

# 1ZAK A

129 161

10 128 162 189

# 1ZAK B

129 161

10 128 162 189

# 2AK3 B

126 161

10 125 162 190

# 2AK3 A

126 161

10 125 162 190

# 3TLX A

155 190

33 154 191 219

# 3TLX B

155 190

33 154 191 219

# 2C9Y A

142 177

20 141 178 206

# 3NDP A

126 161

10 125 162 190

# 3NDP B

126 161

10 125 162 190

# 2BBW A

126 161

10 125 162 190

# 2BBW B

126 161

10 125 162 190

# 2AR7 B

126 161

10 125 162 190

# 2AR7 A

126 161

10 125 162 190

# 1ZD8 A

127 162

11 126 163 191

# 3FB4 A

127 163

5 126 164 191

# 2OSB B

127 162

5 126 163 191

# 2OSB A

127 162

5 126 163 191

# 2ORI A

127 162

5 126 163 191

# 2ORI B

127 162

5 126 163 191

# 2EU8 A

127 162

5 126 163 191

# 2EU8 B

127 162

5 126 163 191

# 3DL0 A

127 162

5 126 163 191

# 3DL0 B

127 162

5 126 163 191

# 1P3J A

127 162

5 126 163 191

# 2QAJ A

127 162

5 126 163 191

# 2QAJ B

127 162

5 126 163 191

# 2OO7 A

127 162

5 126 163 191

# 2OO7 B

127 162

5 126 163 191

# 2P3S A

127 162

5 126 163 191

# 3DKV A

127 162

5 126 163 191

# 1AK2 A

143 178

21 142 179 207

# 2AK2 A

143 178

21 142 179 207

# 3GMT B

123 158

5 122 159 187

# 3GMT A

123 158

5 122 159 187

# 1DVR A

132 167

9 131 168 196

# 1DVR B

132 167

9 131 168 196

# 2AKY A

132 167

9 131 168 196

# 3AKY A

132 167

9 131 168 196

# 1AKY A

132 167

9 131 168 196

# 1S3G A

127 162

5 126 163 191

# 1E4Y B

123 158

5 122 159 187

# 1E4Y A

123 158

5 122 159 187

# 4AKE A

123 158

5 122 159 187

# 4AKE B

123 158

5 122 159 187

# 3HPQ A

123 158

5 122 159 187

# 3HPQ B

123 158

5 122 159 187

# 2ECK B

123 158

5 122 159 187

# 2ECK A

123 158

5 122 159 187

# 1E4V B

123 158

5 122 159 187

# 1E4V A

123 158

5 122 159 187

# 1ANK A

123 158

5 122 159 187

# 1ANK B

123 158

5 122 159 187

# 1AKE B

123 158

5 122 159 187

# 1AKE A

123 158

5 122 159 187

# 3HPR A

123 158

5 122 159 187

# 3HPR B

123 158

5 122 159 187

# 3BE4 A

132 167

10 131 168 196

### PfamID PF05190 ###

# 2O8D B

932 1024

737 931 1025 1065

# 2O8D A

473 569

305 472 570 610

# 2O8F B

932 1024

737 931 1025 1065

# 2O8F A

473 569

305 472 570 610

# 2O8E B

932 1024

737 931 1025 1065

# 2O8E A

473 569

305 472 570 610

# 2O8C B

932 1024

737 931 1025 1065

# 2O8C A

473 569

305 472 570 610

# 2O8B B

932 1024

737 931 1025 1065

# 2O8B A

473 569

305 472 570 610

# 3THW B

701 789

530 700 790 830

# 3THW A

473 569

305 472 570 610

# 3THZ B

701 789

530 700 790 830

# 3THZ A

473 569

305 472 570 610

# 3THY B

701 789

530 700 790 830

# 3THY A

473 569

305 472 570 610

# 3THX B

701 789

530 700 790 830

# 3THX A

473 569

305 472 570 610

# 2WTU A

428 519

271 427 520 560

# 2WTU B

428 519

271 427 520 560

# 1OH7 A

428 519

271 427 520 560

# 1OH7 B

428 519

271 427 520 560

# 1OH5 B

428 519

271 427 520 560

# 1OH5 A

428 519

271 427 520 560

# 1W7A B

428 519

271 427 520 560

# 1W7A A

428 519

271 427 520 560

# 1NG9 A

428 519

271 427 520 560

# 1NG9 B

428 519

271 427 520 560

# 1OH8 B

428 519

271 427 520 560

# 1OH8 A

428 519

271 427 520 560

# 1WBD A

428 519

271 427 520 560

# 1WBD B

428 519

271 427 520 560

# 1WB9 A

428 519

271 427 520 560

# 1WB9 B

428 519

271 427 520 560

# 3K0S A

428 519

271 427 520 560

# 3K0S B

428 519

271 427 520 560

# 1OH6 A

428 519

271 427 520 560

# 1OH6 B

428 519

271 427 520 560

# 1E3M B

428 519

271 427 520 560

# 1E3M A

428 519

271 427 520 560

# 1WBB B

428 519

271 427 520 560

# 1WBB A

428 519

271 427 520 560

# 1NNE B

1403 1494

1254 1402 1495 1535

# 1NNE A

403 494

254 402 495 535

# 1FW6 B

1403 1494

1254 1402 1495 1535

# 1FW6 A

403 494

254 402 495 535

# 1EWQ A

403 494

254 402 495 535

# 1EWQ B

1403 1494

1254 1402 1495 1535

### PfamID PF07085 ###

# 3L31 B

142 245

68 141 246 298

# 3L31 A

142 245

66 141 246 299

# 3L2B A

142 245

66 141 246 297

# 3L2B B

142 245

67 141 246 296

### PfamID PF01000 ###

# 3AOH K

52 180

25 51 181 223

# 3AOH A

52 180

25 51 181 223

# 3AOH F

52 180

25 51 181 223

# 3AOH G

52 180

25 51 181 223

# 3AOH L

52 180

25 51 181 223

# 3AOH B

52 180

25 51 181 223

# 2A6E L

52 180

25 51 181 223

# 2A6E A

52 180

25 51 181 223

# 2A6E B

52 180

25 51 181 223

# 2A6E K

52 180

25 51 181 223

# 2O5J A

52 180

25 51 181 223

# 2O5J B

52 180

25 51 181 223

# 2O5J K

52 180

25 51 181 223

# 2O5J L

52 180

25 51 181 223

# 2BE5 B

52 180

25 51 181 223

# 2BE5 K

52 180

25 51 181 223

# 2BE5 L

52 180

25 51 181 223

# 2BE5 A

52 180

25 51 181 223

# 2A6H A

52 180

25 51 181 223

# 2A6H B

52 180

25 51 181 223

# 2A6H K

52 180

25 51 181 223

# 2A6H L

52 180

25 51 181 223

# 2CW0 A

52 180

25 51 181 223

# 2CW0 L

52 180

25 51 181 223

# 2CW0 K

52 180

25 51 181 223

# 2CW0 B

52 180

25 51 181 223

# 3AOI L

52 180

25 51 181 223

# 3AOI B

52 180

25 51 181 223

# 3AOI F

52 180

25 51 181 223

# 3AOI G

52 180

25 51 181 223

# 3AOI K

52 180

25 51 181 223

# 3AOI A

52 180

25 51 181 223

# 3DXJ K

52 180

25 51 181 223

# 3DXJ B

52 180

25 51 181 223

# 3DXJ A

52 180

25 51 181 223

# 3DXJ L

52 180

25 51 181 223

# 1ZYR L

52 180

25 51 181 223

# 1ZYR K

52 180

25 51 181 223

# 1ZYR A

52 180

25 51 181 223

# 1ZYR B

52 180

25 51 181 223

# 2O5I A

52 180

25 51 181 223

# 2O5I L

52 180

25 51 181 223

# 2O5I K

52 180

25 51 181 223

# 2O5I B

52 180

25 51 181 223

# 3HKZ D

44 157

13 43 158 254

# 3HKZ O

44 157

13 43 158 254

# 2PMZ S

44 157

13 43 158 254

# 2PMZ D

44 157

13 43 158 254

# 2PA8 D

44 157

13 43 158 254

# 1SMY A

52 180

25 51 181 223

# 1SMY B

52 180

25 51 181 223

# 1SMY K

52 180

25 51 181 223

# 1SMY L

52 180

25 51 181 223

# 2A69 K

52 180

25 51 181 223

# 2A69 L

52 180

25 51 181 223

# 2A69 B

52 180

25 51 181 223

# 2A69 A

52 180

25 51 181 223

# 2PPB K

52 180

25 51 181 223

# 2PPB L

52 180

25 51 181 223

# 2PPB B

52 180

25 51 181 223

# 2PPB A

52 180

25 51 181 223

# 2A68 L

52 180

25 51 181 223

# 2A68 A

52 180

25 51 181 223

# 2A68 B

52 180

25 51 181 223

# 2A68 K

52 180

25 51 181 223

# 1IW7 B

52 180

25 51 181 223

# 1IW7 L

52 180

25 51 181 223

# 1IW7 K

52 180

25 51 181 223

# 1IW7 A

52 180

25 51 181 223

# 3EQL B

52 180

25 51 181 223

# 3EQL L

52 180

25 51 181 223

# 3EQL K

52 180

25 51 181 223

# 3EQL A

52 180

25 51 181 223

# 2Y0S S

44 157

13 43 158 254

# 2Y0S D

44 157

13 43 158 254

# 1HQM B

52 178

25 51 179 222

# 1HQM A

52 178

25 51 179 222

# 1YNJ A

52 179

25 51 180 223

# 1YNJ B

52 179

25 51 180 223

# 1YNN A

52 179

25 51 180 223

# 1YNN B

52 179

25 51 180 223

# 1I6V A

52 179

25 51 180 223

# 1I6V B

52 179

25 51 180 223

# 2GHO A

52 179

25 51 180 223

# 2GHO B

52 179

25 51 180 223

# 3GTK C

49 171

18 48 172 256

# 1TWH C

49 171

18 48 172 256

# 3HOV C

49 171

18 48 172 256

# 3S17 C

49 171

18 48 172 256

# 3I4N C

49 171

18 48 172 256

# 1Y1W C

49 171

18 48 172 256

# 3PO2 C

49 171

18 48 172 256

# 3S2D C

49 171

18 48 172 256

# 2JA8 C

49 171

18 48 172 256

# 3RZD C

49 171

18 48 172 256

# 3S14 C

49 171

18 48 172 256

# 2JA6 C

49 171

18 48 172 256

# 3HOX C

49 171

18 48 172 256

# 4A3I C

49 171

18 48 172 256

# 2NVY C

49 171

18 48 172 256

# 3HOZ C

49 171

18 48 172 256

# 1Y77 C

49 171

18 48 172 256

# 3HOU O

49 171

18 48 172 256

# 3HOU C

49 171

18 48 172 256

# 2B8K C

49 171

18 48 172 256

# 4A3F C

49 171

18 48 172 256

# 1SFO C

49 171

18 48 172 256

# 2JA7 O

49 171

18 48 172 256

# 2JA7 C

49 171

18 48 172 256

# 1TWG C

49 171

18 48 172 256

# 3GTL C

49 171

18 48 172 256

# 3QT1 C

49 171

18 48 172 256

# 3H3V D

49 171

18 48 172 256

# 4A3K C

49 171

18 48 172 256

# 1NIK C

49 171

18 48 172 256

# 3FKI C

49 171

18 48 172 256

# 3S1Q C

49 171

18 48 172 256

# 3S1M C

49 171

18 48 172 256

# 3S2H C

49 171

18 48 172 256

# 2JA5 C

49 171

18 48 172 256

# 2NVT C

49 171

18 48 172 256

# 3K7A C

49 171

18 48 172 256

# 3GTP C

49 171

18 48 172 256

# 3M4O C

49 171

18 48 172 256

# 3HOY C

49 171

18 48 172 256

# 3GTG C

49 171

18 48 172 256

# 2R92 C

49 171

18 48 172 256

# 2B63 C

49 171

18 48 172 256

# 2E2J C

49 171

18 48 172 256

# 3S16 C

49 171

18 48 172 256

# 3S1R C

49 171

18 48 172 256

# 1TWA C

49 171

18 48 172 256

# 2YU9 C

49 171

18 48 172 256

# 4A3J C

49 171

18 48 172 256

# 2NVZ C

49 171

18 48 172 256

# 2R93 C

49 171

18 48 172 256

# 3GTJ C

49 171

18 48 172 256

# 4A3M C

49 171

18 48 172 256

# 2NVQ C

49 171

18 48 172 256

# 4A3C C

49 171

18 48 172 256

# 4A3E C

49 171

18 48 172 256

# 2E2I C

49 171

18 48 172 256

# 3PO3 C

49 171

18 48 172 256

# 2VUM C

49 171

18 48 172 256

# 3S15 C

49 171

18 48 172 256

# 4A3B C

49 171

18 48 172 256

# 1R9T C

49 171

18 48 172 256

# 3CQZ C

49 171

18 48 172 256

# 2R7Z C

49 171

18 48 172 256

# 1TWF C

49 171

18 48 172 256

# 3GTO C

49 171

18 48 172 256

# 1TWC C

49 171

18 48 172 256

# 3S1N C

49 171

18 48 172 256

# 1I6H C

49 171

18 48 172 256

# 1WCM C

49 171

18 48 172 256

# 2NVX C

49 171

18 48 172 256

# 3GTM C

49 171

18 48 172 256

# 4A3G C

49 171

18 48 172 256

# 1Y1V C

49 171

18 48 172 256

# 3K1F C

49 171

18 48 172 256

# 4A93 C

49 171

18 48 172 256

# 1I50 C

49 171

18 48 172 256

# 1K83 C

49 171

18 48 172 256

# 1R9S C

49 171

18 48 172 256

# 4A3L C

49 171

18 48 172 256

# 3GTQ C

49 171

18 48 172 256

# 4A3D C

49 171

18 48 172 256

# 3I4M C

49 171

18 48 172 256

# 2E2H C

49 171

18 48 172 256

# 1R5U C

49 171

18 48 172 256

# 1I3Q C

49 171

18 48 172 256

# 3M3Y C

49 171

18 48 172 256

# 3HOW C

49 171

18 48 172 256

# 3H0G O

48 172

17 47 173 257

# 3H0G C

48 172

17 47 173 257

# 1BDF B

58 186

28 57 187 229

# 1BDF C

58 186

28 57 187 229

# 1BDF D

58 186

28 57 187 229

# 1BDF A

58 186

28 57 187 229

# 3IYD A

58 186

28 57 187 229

# 3IYD B

58 186

28 57 187 229

# 3LU0 A

58 186

28 57 187 229

# 3LU0 B

58 186

28 57 187 229

### PfamID PF03489 ###

# 1QDM C

276 310

43 275 311 338 378 467

# 1QDM B

276 310

43 275 311 338 378 467

# 1QDM A

276 310

43 275 311 338 378 467

### PfamID PF05184 ###

# 1QDM C

339 377

43 275 311 338 378 467

# 1QDM B

339 377

43 275 311 338 378 467

# 1QDM A

339 377

43 275 311 338 378 467

### PfamID PF01909 ###

# 1FA0 A

72 161

4 71 162 352

# 1FA0 B

72 161

4 71 162 352

# 2O1P A

72 161

4 71 162 352

# 2O1P B

72 161

4 71 162 352

# 2HHP A

72 161

4 71 162 352

# 3C66 A

72 161

4 71 162 352

# 3C66 B

72 161

4 71 162 352

# 2Q66 A

72 161

5 71 162 352

# 1F5A A

75 175

20 74 176 365

### PfamID PF05204 ###

# 1LWT A

302 413

1 194 297 301 414 454

# 1LWT A

195 296

1 194 297 301 414 454

# 1UM2 B

585 696

285 477 580 584 697 737

# 1UM2 B

478 579

285 477 580 584 697 737

# 1UM2 A

585 696

285 477 580 584 697 737

# 1UM2 A

478 579

285 477 580 584 697 737

# 1JVA B

585 696

284 477 580 584 697 737

# 1JVA B

478 579

284 477 580 584 697 737

# 1JVA A

585 696

284 477 580 584 697 737

# 1JVA A

478 579

284 477 580 584 697 737

# 1VDE A

302 413

1 194 297 301 414 454

# 1VDE A

195 296

1 194 297 301 414 454

# 1VDE B

302 413

1 194 297 301 414 454

# 1VDE B

195 296

1 194 297 301 414 454

# 1EF0 B

302 413

2 205 297 301 414 454

# 1EF0 B

206 296

2 205 297 301 414 454

# 1EF0 A

302 413

2 194 297 301 414 454

# 1EF0 A

195 296

2 194 297 301 414 454

# 1DFA A

302 413

1 199 297 301 414 454

# 1DFA A

200 296

1 199 297 301 414 454

# 1LWS A

302 413

1 194 297 301 414 454

# 1LWS A

195 296

1 194 297 301 414 454

### PfamID PF00670 ###

# 3GVP D

370 531

184 369 532 607

# 3GVP B

370 531

184 369 532 606

# 3GVP C

370 531

184 369 532 607

# 3GVP A

370 531

184 369 532 607

# 1K0U F

190 351

4 189 352 430

# 1K0U E

190 351

4 189 352 430

# 1K0U H

190 351

4 189 352 430

# 1K0U C

190 351

4 189 352 430

# 1K0U A

190 351

4 189 352 430

# 1K0U B

190 351

4 189 352 430

# 1K0U G

190 351

4 189 352 430

# 1K0U D

190 351

4 189 352 430

# 1B3R A

190 351

4 189 352 430

# 1B3R B

190 351

4 189 352 430

# 1B3R C

190 351

4 189 352 430

# 1B3R D

190 351

4 189 352 430

# 1XWF D

190 351

4 189 352 430

# 1XWF B

190 351

4 189 352 430

# 1XWF C

190 351

4 189 352 430

# 1XWF A

190 351

4 189 352 430

# 1KY4 A

190 351

4 189 352 430

# 1KY4 D

3190 3351

3004 3189 3352 3430

# 1KY4 C

2190 2351

2004 2189 2352 2430

# 1KY4 B

1190 1351

1004 1189 1352 1430

# 2H5L D

190 351

4 189 352 430

# 2H5L E

190 351

4 189 352 430

# 2H5L F

190 351

4 189 352 430

# 2H5L H

190 351

4 189 352 430

# 2H5L B

190 351

4 189 352 430

# 2H5L C

190 351

4 189 352 430

# 2H5L A

190 351

4 189 352 430

# 2H5L G

190 351

4 189 352 430

# 1KY5 D

3190 3351

3004 3189 3352 3430

# 1KY5 B

1190 1351

1004 1189 1352 1430

# 1KY5 A

190 351

4 189 352 430

# 1KY5 C

2190 2351

2004 2189 2352 2430

# 1D4F B

190 351

4 189 352 430

# 1D4F C

190 351

4 189 352 430

# 1D4F D

190 351

4 189 352 430

# 1D4F A

190 351

4 189 352 430

# 3N58 A

227 386

8 226 387 465

# 3N58 B

227 386

8 226 387 465

# 3N58 D

227 386

8 226 387 465

# 3N58 C

227 386

8 226 387 465

# 3ONF A

240 403

12 239 404 484

# 3ONF B

240 403

12 239 404 484

# 3ONE B

240 403

12 239 404 484

# 3ONE A

240 403

12 239 404 484

# 3OND B

240 403

12 239 404 484

# 3OND A

240 403

12 239 404 484

# 2ZJ1 C

253 415

19 252 416 494

# 2ZJ1 A

253 415

19 252 416 494

# 2ZJ1 B

253 415

19 252 416 494

# 2ZJ1 D

253 415

19 252 416 494

# 3DHY B

253 415

19 252 416 494

# 3DHY D

253 415

19 252 416 494

# 3DHY A

253 415

19 252 416 494

# 3DHY C

253 415

19 252 416 494

# 2ZIZ C

253 415

19 252 416 494

# 2ZIZ A

253 415

19 252 416 494

# 2ZIZ B

253 415

19 252 416 494

# 2ZIZ D

253 415

19 252 416 494

# 2ZJ0 D

253 415

19 252 416 494

# 2ZJ0 B

253 415

19 252 416 494

# 2ZJ0 A

253 415

19 252 416 494

# 2ZJ0 C

253 415

19 252 416 494

# 3CE6 B

253 415

19 252 416 494

# 3CE6 C

253 415

19 252 416 494

# 3CE6 A

253 415

19 252 416 494

# 3CE6 D

253 415

19 252 416 494

# 3H9U D

190 351

4 189 352 436

# 3H9U A

190 351

4 189 352 436

# 3H9U C

190 351

4 189 352 436

# 3H9U B

190 351

4 189 352 436

# 3MTG B

289 450

103 288 451 529

# 3MTG A

289 450

103 288 451 529

# 3GLQ B

234 393

12 233 394 472

# 3GLQ A

234 393

12 233 394 472

# 3D64 A

234 393

12 233 394 472

# 3D64 B

234 393

12 233 394 472

# 1A7A A

191 352

5 190 353 431

# 1A7A B

191 352

5 190 353 431

# 3NJ4 C

191 352

5 190 353 431

# 3NJ4 B

191 352

5 190 353 431

# 3NJ4 A

191 352

5 190 353 431

# 3NJ4 D

191 352

5 190 353 431

# 1LI4 A

191 352

5 190 353 431

# 1V8B B

235 397

4 234 398 478

# 1V8B A

235 397

4 234 398 478

# 1V8B C

235 397

4 234 398 478

# 1V8B D

235 397

4 234 398 478

# 3G1U C

190 351

3 189 352 431

# 3G1U B

190 351

3 189 352 420

# 3G1U D

190 351

3 189 352 423

# 3G1U A

190 351

3 189 352 420

### PfamID PF03199 ###

# 3KAJ A

229 331

29 228 332 498

# 3KAJ B

229 331

29 228 332 498

# 3KAL A

229 331

29 228 332 498

# 3KAL B

229 331

29 228 332 498

# 3KAK A

229 331

29 228 332 498

# 3KAK B

229 331

30 228 332 498

# 2WYO A

252 360

8 251 361 553

# 2WYO C

252 360

8 251 361 553

# 2WYO D

252 360

8 251 361 553

# 2WYO B

252 360

8 251 361 553

# 2HGS A

202 302

5 201 303 473

# 1M0W B

1217 1321

1005 1216 1322 1490

# 1M0W A

216 321

6 215 322 490

# 1M0T A

216 321

5 215 322 490

# 1M0T B

1216 1321

1005 1215 1322 1490

### PfamID PF02826 ###

# 3DC2 A

108 282

7 107 283 314

# 3DC2 B

108 282

7 107 283 314

# 1YGY A

108 282

7 107 283 314

# 1YGY B

108 282

7 107 283 314

# 3DDN A

108 282

7 107 283 314

# 3DDN B

108 282

7 107 283 314

# 3K5P A

118 295

14 117 296 327

# 2OME H

139 323

36 138 324 358

# 2OME C

139 323

36 138 324 358

# 2OME B

139 323

36 138 324 358

# 2OME E

139 323

36 138 324 358

# 2OME F

139 323

36 138 324 358

# 2OME A

139 323

36 138 324 358

# 2OME G

139 323

36 138 324 358

# 2OME D

139 323

36 138 324 358

# 2GO1 A

155 334

56 154 335 365

# 2NAC A

155 334

56 154 335 365

# 2NAC B

155 334

56 154 335 365

# 2NAD A

155 334

56 154 335 365

# 2NAD B

155 334

56 154 335 365

# 3OET D

104 256

3 103 257 285

# 3OET F

104 256

3 103 257 280

# 3OET C

104 256

3 103 257 285

# 3OET A

104 256

3 103 257 285

# 3OET G

104 256

3 103 257 285

# 3OET B

104 256

3 103 257 285

# 3OET E

104 256

3 103 257 285

# 3OET H

104 256

3 103 257 285

# 2CUK C

107 279

3 106 280 311

# 2CUK A

107 279

3 106 280 311

# 2CUK D

107 279

3 106 280 311

# 2CUK B

107 279

3 106 280 311

# 2W2L D

122 306

17 121 307 336

# 2W2L A

122 306

17 121 307 336

# 2W2L C

122 306

17 121 307 336

# 2W2L B

122 306

17 121 307 336

# 2W2K A

122 306

17 121 307 336

# 2W2K B

122 306

17 121 307 336

# 2DLD A

112 299

4 111 300 331

# 2DLD B

112 299

4 111 300 331

# 1MX3 A

133 317

30 132 318 352

# 2G76 B

110 284

8 109 285 306

# 2G76 A

110 284

8 109 285 306

# 1QP8 B

91 262

25 90 263 296

# 1QP8 A

91 262

25 90 263 296

# 3GVX B

90 257

17 89 258 282

# 3GVX A

90 257

17 89 258 281

# 4DGS A

112 285

10 111 286 316

# 3KB6 B

106 296

3 105 297 328

# 3KB6 A

106 296

3 105 297 328

# 3KB6 D

106 296

3 105 297 328

# 3KB6 C

106 296

3 105 297 328

# 3BA1 A

109 281

6 108 282 313

# 3BAZ A

109 281

6 108 282 313

# 3FN4 A

155 334

53 154 335 365

# 2GSD A

155 334

53 154 335 365

# 3GA0 A

122 306

19 121 307 341

# 2HU2 A

122 306

19 121 307 341

# 1HL3 A

122 306

19 121 307 341

# 2Q50 C

116 295

9 115 296 326

# 2Q50 A

116 295

9 115 296 326

# 2Q50 D

116 295

9 115 296 326

# 2Q50 B

116 295

9 115 296 326

# 2GCG D

116 295

9 115 296 327

# 2GCG C

116 295

9 115 296 327

# 2GCG A

116 295

9 115 296 327

# 2GCG B

116 295

9 115 296 327

# 2WWR D

116 295

9 115 296 325

# 2WWR A

116 295

9 115 296 327

# 2WWR C

116 295

9 115 296 327

# 2WWR B

116 295

9 115 296 327

# 2H1S C

116 295

9 115 296 326

# 2H1S D

116 295

9 115 296 326

# 2H1S A

116 295

9 115 296 326

# 2H1S B

116 295

9 115 296 326

# 1WWK A

105 280

3 104 281 304

# 1WWK B

105 280

3 104 281 302

# 2D0I B

106 286

5 105 287 318

# 2D0I C

106 286

5 105 287 318

# 2D0I D

106 286

5 105 287 318

# 2D0I A

106 286

5 105 287 318

# 3JTM A

155 334

56 154 335 365

# 3NAQ B

155 334

56 154 335 365

# 3NAQ A

155 334

56 154 335 365

# 3N7U J

155 334

56 154 335 365

# 3N7U G

155 334

56 154 335 365

# 3N7U E

155 334

56 154 335 365

# 3N7U H

155 334

56 154 335 365

# 3N7U D

155 334

56 154 335 365

# 3N7U C

155 334

56 154 335 365

# 3N7U I

155 334

56 154 335 365

# 3N7U L

155 334

56 154 335 365

# 3N7U A

155 334

56 154 335 365

# 3N7U F

155 334

56 154 335 365

# 3N7U K

155 334

56 154 335 365

# 3N7U B

155 334

56 154 335 365

# 2DBZ A

109 290

5 108 291 322

# 2DBZ B

109 290

5 108 291 322

# 2DBR D

109 290

5 108 291 322

# 2DBR F

109 290

5 108 291 322

# 2DBR A

109 290

5 108 291 322

# 2DBR B

109 290

5 108 291 322

# 2DBR C

109 290

5 108 291 322

# 2DBR E

109 290

5 108 291 322

# 2DBQ A

109 290

5 108 291 322

# 2EKL A

110 285

8 109 286 312

# 3EVT A

103 278

4 102 279 309

# 1J4A B

112 299

4 111 300 331

# 1J4A A

112 299

4 111 300 331

# 1J4A D

112 299

4 111 300 331

# 1J4A C

112 299

4 111 300 331

# 1J49 A

112 299

4 111 300 331

# 1J49 B

112 299

4 111 300 331

# 2P9E B

117 294

13 116 295 326

# 2P9E D

117 294

13 116 295 326

# 2P9E C

117 294

13 116 295 326

# 2P9E A

117 294

13 116 295 326

# 1SC6 B

117 294

13 116 295 326

# 1SC6 A

117 294

13 116 295 326

# 1SC6 C

117 291

13 116 292 326

# 1SC6 D

117 291

13 116 292 326

# 2P9G A

117 294

13 116 295 326

# 2P9G B

117 294

13 116 295 326

# 2P9C A

117 294

13 116 295 326

# 2P9C B

117 294

13 116 295 326

# 1YBA D

117 294

13 116 295 326

# 1YBA B

117 294

13 116 295 326

# 1YBA A

117 294

13 116 295 326

# 1YBA C

117 294

13 116 295 326

# 2PA3 A

117 294

13 116 295 326

# 1PSD A

117 294

13 116 295 326

# 1PSD B

117 294

13 116 295 326

# 3GG9 A

125 311

14 124 312 343

# 3GG9 B

125 311

14 124 312 343

# 3GG9 D

125 311

14 124 312 343

# 3GG9 C

125 311

14 124 312 343

# 2FSS A

128 313

25 127 314 344

# 2FSS C

128 313

25 127 314 344

# 2FSS B

128 313

25 127 314 344

# 2FSS D

128 313

25 127 314 344

# 2J6I D

128 313

25 127 314 344

# 2J6I A

128 313

25 127 314 344

# 2J6I B

128 313

25 127 314 344

# 2J6I C

128 313

25 127 314 344

# 4E5N C

109 294

5 108 295 326

# 4E5N G

109 294

5 108 295 326

# 4E5N D

109 294

5 108 295 326

# 4E5N B

109 294

5 108 295 326

# 4E5N F

109 294

5 108 295 326

# 4E5N E

109 294

5 108 295 326

# 4E5N H

109 294

5 108 295 326

# 4E5N A

109 294

5 108 295 326

# 4EBF F

109 294

5 108 295 326

# 4EBF C

109 294

5 108 295 326

# 4EBF D

109 294

5 108 295 326

# 4EBF A

109 294

5 108 295 326

# 4EBF E

109 294

5 108 295 322

# 4EBF B

109 294

5 108 295 326

# 4E5K B

109 294

5 108 295 326

# 4E5K C

109 294

5 108 295 326

# 4E5K A

109 294

5 108 295 326

# 4E5K D

109 294

5 108 295 326

# 4E5P E

109 294

5 108 295 326

# 4E5P C

109 294

5 108 295 326

# 4E5P B

109 294

5 108 295 326

# 4E5P F

109 294

5 108 295 326

# 4E5P A

109 294

5 108 295 326

# 4E5P D

109 294

5 108 295 326

# 4E5M B

109 294

5 108 295 326

# 4E5M A

109 294

5 108 295 326

# 1GDH A

110 289

5 109 290 321

# 1GDH B

110 289

5 109 290 321

# 2O4C B

101 256

4 100 257 341

# 2O4C A

101 256

4 100 257 341

# 1DXY A

110 297

3 109 298 329

### PfamID PF04561 ###

# 3H0G B

204 393

29 203 394 450

# 3H0G N

204 393

29 203 394 450

# 3S17 B

219 407

42 218 408 464

# 1I6H B

219 407

42 218 408 464

# 4A3B B

219 407

42 218 408 464

# 3K1F B

219 407

42 218 408 464

# 4A3I B

219 407

42 218 408 464

# 1TWA B

219 407

42 218 408 464

# 3S14 B

219 407

42 218 408 464

# 3S15 B

219 407

42 218 408 464

# 2NVX B

219 407

42 218 408 464

# 3M3Y B

219 407

42 218 408 464

# 3K7A B

219 407

42 218 408 464

# 1R9T B

219 407

42 218 408 464

# 2JA8 B

219 407

42 218 408 464

# 4A3M B

219 407

42 218 408 464

# 2JA5 B

219 407

42 218 408 464

# 3HOY B

219 407

42 218 408 464

# 2JA7 B

219 407

42 218 408 464

# 2JA7 N

219 407

42 218 408 464

# 3I4M B

219 407

42 218 408 464

# 3S1M B

219 407

42 218 408 464

# 3HOU N

219 407

42 218 408 464

# 3HOU B

219 407

42 218 408 464

# 2R92 B

219 407

42 218 408 464

# 2B63 B

219 407

42 218 408 464

# 3HOW B

219 407

42 218 408 464

# 3HOV B

219 407

42 218 408 464

# 2VUM B

219 407

42 218 408 464

# 4A3F B

219 407

42 218 408 464

# 3S2H B

219 407

42 218 408 464

# 3GTP B

219 407

42 218 408 464

# 3FKI B

219 407

42 218 408 464

# 4A93 B

219 407

42 218 408 464

# 2E2H B

219 407

42 218 408 464

# 4A3D B

219 407

42 218 408 464

# 2E2J B

219 407

42 218 408 464

# 1Y77 B

219 407

42 218 408 464

# 1TWF B

219 407

42 218 408 464

# 2NVT B

219 407

42 218 408 464

# 1I50 B

219 407

42 218 408 464

# 1TWG B

219 407

42 218 408 464

# 3S1R B

219 407

42 218 408 464

# 1K83 B

219 407

42 218 408 464

# 1Y1V B

219 407

42 218 408 464

# 2R93 B

219 407

42 218 408 464

# 2B8K B

219 407

42 218 408 464

# 3QT1 B

219 407

42 218 408 464

# 3PO3 B

219 407

42 218 408 464

# 3S16 B

219 407

42 218 408 464

# 3M4O B

219 407

42 218 408 464

# 3GTL B

219 407

42 218 408 464

# 3GTG B

219 407

42 218 408 464

# 4A3G B

219 407

42 218 408 464

# 1NIK B

219 407

42 218 408 464

# 3GTK B

219 407

42 218 408 464

# 1I3Q B

219 407

42 218 408 464

# 3S1Q B

219 407

42 218 408 464

# 3S2D B

219 407

42 218 408 464

# 1Y1W B

219 407

42 218 408 464

# 2YU9 B

219 407

42 218 408 464

# 4A3L B

219 407

42 218 408 464

# 4A3E B

219 407

42 218 408 464

# 1R5U B

219 407

42 218 408 464

# 4A3K B

219 407

42 218 408 464

# 3RZD B

219 407

42 218 408 464

# 3I4N B

219 407

42 218 408 464

# 3S1N B

219 407

42 218 408 464

# 2NVY B

219 407

42 218 408 464

# 1TWH B

219 407

42 218 408 464

# 3CQZ B

219 407

42 218 408 464

# 1SFO B

219 407

42 218 408 464

# 3H3V C

219 407

42 218 408 464

# 3GTM B

219 407

42 218 408 464

# 2E2I B

219 407

42 218 408 464

# 3HOX B

219 407

42 218 408 464

# 3GTJ B

219 407

42 218 408 464

# 3HOZ B

219 407

42 218 408 464

# 1R9S B

219 407

42 218 408 464

# 1WCM B

219 407

42 218 408 464

# 3PO2 B

219 407

42 218 408 464

# 2NVQ B

219 407

42 218 408 464

# 4A3J B

219 407

42 218 408 464

# 3GTO B

219 407

42 218 408 464

# 2JA6 B

219 407

42 218 408 464

# 3GTQ B

219 407

42 218 408 464

# 2R7Z B

219 407

42 218 408 464

# 1TWC B

219 407

42 218 408 464

# 4A3C B

219 407

42 218 408 464

# 2NVZ B

219 407

42 218 408 464

# 2Y0S R

171 347

25 170 348 404

# 2Y0S B

171 347

25 170 348 404

# 3IYD C

151 228

26 150 229 328 455 509

# 3IYD C

329 454

26 150 229 328 455 509

# 3LU0 C

151 276

26 150 277 328 455 509

# 3LU0 C

329 454

26 150 277 328 455 509

# 1SMY M

142 334

17 141 335 389

# 1SMY C

142 334

17 141 335 389

# 3DXJ M

142 334

17 141 335 389

# 3DXJ C

142 334

17 141 335 389

# 3AOI C

142 334

17 141 335 389

# 3AOI H

142 334

17 141 335 389

# 3AOI M

142 334

17 141 335 389

# 2A68 M

142 334

17 141 335 389

# 2A68 C

142 334

17 141 335 389

# 1ZYR C

142 334

17 141 335 389

# 1ZYR M

142 334

17 141 335 389

# 3AOH H

142 334

17 141 335 389

# 3AOH C

142 334

17 141 335 389

# 3AOH M

142 334

17 141 335 389

# 1IW7 M

142 334

17 141 335 389

# 1IW7 C

142 334

17 141 335 389

# 2O5J C

142 334

17 141 335 389

# 2O5J M

142 334

17 141 335 389

# 2CW0 M

142 334

17 141 335 389

# 2CW0 C

142 334

17 141 335 389

# 2O5I M

142 334

17 141 335 389

# 2O5I C

142 334

17 141 335 389

# 2BE5 C

142 334

17 141 335 389

# 2BE5 M

142 334

17 141 335 389

# 2A6H C

142 334

17 141 335 389

# 2A6H M

142 334

17 141 335 389

# 3EQL M

142 334

17 141 335 389

# 3EQL C

142 334

17 141 335 389

# 2A6E C

142 334

17 141 335 389

# 2A6E M

142 334

17 141 335 389

# 2A69 C

142 334

17 141 335 389

# 2A69 M

142 334

17 141 335 389

# 2PPB C

142 334

17 141 335 389

# 2PPB M

142 334

17 141 335 389

# 1YNN C

142 334

17 141 335 389

# 1I6V C

142 334

17 141 335 389

# 1HQM C

142 334

17 141 335 389

# 1YNJ C

142 334

17 141 335 389

# 2GHO C

142 334

17 141 335 389
