# Supplementary material for: In the Multi-domain Protein Adenylate Kinase, Domain Insertion Facilitates Cooperative Folding while Accommodating Function at Domain Interfaces
Source: PLoS Comput Biol. 2014 Nov 13;10(11):e1003938. doi: 10.1371/journal.pcbi.1003938 (PMC4230728; doi:10.1371/journal.pcbi.1003938)
Supplement: Text S5 — Supporting references. (PDF) [file pcbi.1003938.s013.pdf]

SUPPORTING REFERENCES

1. Noel JK, Whitford PC, Sanbonmatsu KY, Onuchic JN (2010) SMOG@ctbp: simplified deployment of structure-based models in GROMACS. Nucleic Acids Res 38: W657–61.

2. Clementi C, Nymeyer H, Onuchic JN (2000) Topological and energetic factors: what determines the structural details of the transition state ensemble and “en-route” intermediates for protein folding? An investigation for small globular proteins. J Mol Biol 298: 937–53.

3. Sobolev V, Sorokine A, Prilusky J, Abola E, Edelman M (1999) Automated analysis of interatomic contacts in proteins. Bioinformatics 15: 327–332.

4. Whitford PC, Miyashita O, Levy Y, Onuchic JN (2007) Conformational transitions of adenylate kinase: switching by cracking. J Mol Biol 366: 1661–71.

5. Fiser A, Do RK, Sali A (2000) Modeling of loops in protein structures. Protein Sci 9: 1753–73.

6. Sali A, Blundell TL (1993) Comparative protein modelling by satisfaction of spatial restraints. J Mol Biol 234: 779–815.

7. Hess B, Kutzner C, van der Spoel D, Lindahl E (2008) GROMACS 4: Algorithms for Highly Efficient, Load-Balanced, and Scalable Molecular Simulation. J Chem Theory Comput 4: 435–447.

8. Best RB, Hummer G, Eaton WA (2013) Native contacts determine protein folding mechanisms in atomistic simulations. Proc Natl Acad Sci USA 110: 17874-79.

9. Frickey T, Lupas A (2004) CLANS: a Java application for visualizing protein families based on pairwise similarity. Bioinformatics 20: 3702–4.

10. Rundqvist L, Adén J, Sparrman T, Wallgren M, Olsson U, et al. (2009) Noncooperative folding of subdomains in adenylate kinase. Biochemistry. 48: 1911–27.

11. Miller RG (1974) The Jackknife - a review. Biometrika 61:1-15

12. Efron B, Stein C (1981) The Jackknife estimate of variance. Ann. Stat. 9:586-96.

13. Knott M, Kaya H, Chan HS (2004) Energetics of protein thermodynamic cooperativity : contributions of local and non-local interactions. Polymer 45:623-32

14. Finn RD, Bateman A, Clements J, Coggill P, Eberhardt RY et al (2014) Pfam: the protein families database. Nucleic Acids Res. 42:D222-D230.

15. Lopez G, Valencia A, Tress M (2006) FireDB-a database of functionally important residues from proteins of known structure. Nucleic Acids Res. 35: D219-D223.

16. Bae E, Phillips GN (2006) Roles of static and dynamic domains in stability and catalysis of adenylate kinase. Proc Natl Acad Sci USA 103: 2132–7.

17. Ruan Q, Ruan K, Balny C, Glaser M, Mantulin WW (2001) Protein folding pathways of adenylate kinase from E. coli: hydrostatic pressure and stopped-flow studies. Biochemistry 40: 14706–14.

18. Orevi T, Ben Ishay E, Pirchi M, Jacob MH, Amir D, et al. (2009) Early closure of a long loop in the refolding of adenylate kinase: a possible key role of non-local interactions in the initial folding steps. J Mol Biol 385: 1230–42.

19. Ben Ishay E, Rahamim G, Orevi T, Hazan G, Amir D, et al. (2012) Fast subdomain folding prior to the global refolding transition of E. coli adenylate kinase: a double kinetics study. J Mol Biol 423: 613–23.

20. Ratner V, Sinev M, Haas E (2000) Determination of intramolecular distance distribution during protein folding on the millisecond timescale. J Mol Biol 299: 1363–71.

21. Ratner V, Amir D, Kahana E, Haas E (2005) Fast collapse but slow formation of secondary structure elements in the refolding transition of E. coli adenylate kinase. J Mol Biol 352: 683– 99.

22. Ratner V, Kahana E, Haas E (2002) The natively helical chain segment 169-188 of Escherichia coli adenylate kinase is formed in the latest phase of the refolding transition. J Mol Biol 320: 1135–45.
